# Beyond |**E**|^2^: Tensorial Electrostatic Reorganization in Hydrated Eu^3+^–Nitrate Complexes and Its Implications for Hypersensitive Luminescence

**DOI:** 10.1021/acs.jpcb.6c05370

**Published:** 2026-10-13

**Authors:** Murilo Sodré Marques

**Affiliations:** Programa de Pós—Graduação em Ciência dos Materiais (PROMAT) Centro das Ciências Exatas e das Tecnologias (CCET), 423876 Universidade Federal do Oeste da Bahia (UFOB), Rua da Prainha, 1326, Morada Nobre, CEP 47810-047 Barreiras, Bahia, Brazil

## Abstract

The microscopic origin of the hypersensitive luminescence of Eu^3+^ remains closely connected to how the local coordination environment perturbs the 4f electronic manifold. Here, we combine molecular dynamics, free-energy calculations, a multipolar analysis of the local electrostatic environment, and multireference electronic-structure calculations to examine hydrated Eu^3+^–nitrate complexes beyond a scalar static-field description. A 12–6–4 Eu^3+^ model combined with TIP4P-Ew water yields an approximately nine-coordinate hydration shell and predicts the local outer-sphere nitrate basin to be more stable than the inner-sphere basin by about 2.6 kcal mol^–1^. Despite large changes in nitrate proximity, the total electric-field magnitude at Eu^3+^ remains essentially invariant: the inner–far contrast is only 0.0022 V Å^–1^, with a negligible standardized effect (*d* = 0.02). The same near invariance holds for the inner–far contrasts of the total rank-2 and rank-3 electrostatic tensor norms. In contrast, the nitrate contribution is amplified by factors of approximately 4.5, 9.8, and 23.7 from electrostatic ranks *k* = 1 to 3, respectively, but is masked by progressively stronger solvent–anion cancellation. The odd-rank *k* = 3 tensor nevertheless retains a clear coordination signature in its angular composition: the broad interconversion-region conditional ensemble exhibits an enhanced axial *q* = 0 fraction relative to the far-window reference (Δ*p*_0_ = 0.181, source-cluster 95% CI [0.086, 0.275], *d* = 0.83). This qualitative *q* = 0 enrichment persists in matched explicit-MD stress tests in which the transferred Eu–nitrate *C*_4_ coefficients are coherently scaled by ±20%. CASSCF/SC-NEVPT2 calculations including spin–orbit coupling provide a consistent description of the low-lying 4f^6^ manifolds, whereas an explicit quantitative treatment of 4f^6^–4f^5^5d^1^ opposite-parity mixing remains beyond the balanced active-space description achieved here. These results show that hydrated Eu^3+^–nitrate speciation is poorly encoded in |**E**|^2^ alone and is instead expressed through rank-dependent cancellation and the angular structure of the local electrostatic potential. The resulting tensorial descriptors provide a symmetry-resolved electrostatic framework that may inform microscopic interpretations of Eu^3+^ hypersensitive luminescence, while a quantitative connection to transition intensities requires an explicit and balanced treatment of the relevant opposite-parity electronic configurations.

## Graphical abstract

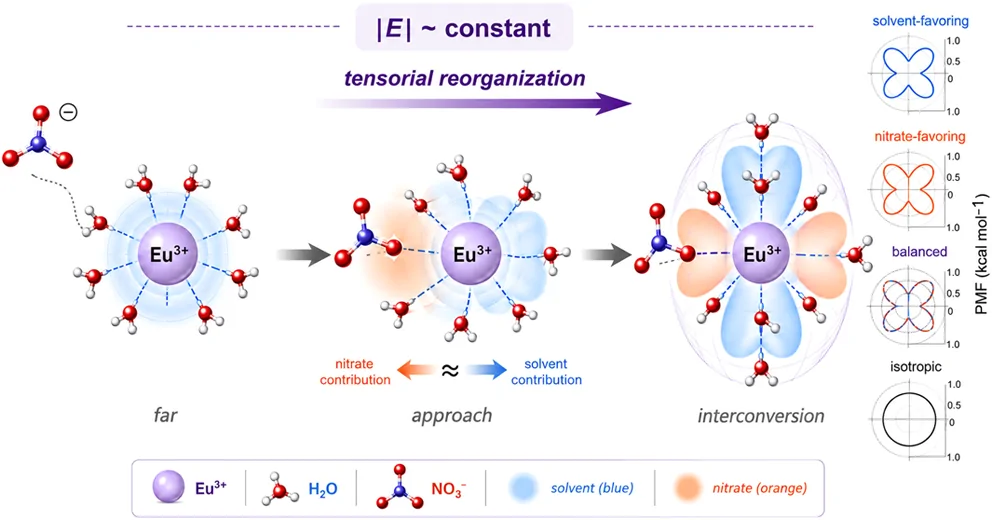

## Introduction

The optical spectroscopy of trivalent lanthanide ions occupies a unique position at the interface between coordination chemistry, electronic structure, and photophysics. The shielding of the 4*f* shell by the filled 5*s* and 5*p* subshells gives rise to relatively sharp intraconfigurational transitions whose energies are only moderately perturbed by the surrounding chemical environment, while their intensities and fine structures may remain remarkably sensitive to local coordination.^1–3^ Among the trivalent lanthanides, Eu^3+^ is especially useful as a spectroscopic probe because its 4f^6^ configuration gives rise to a comparatively simple visible emission spectrum dominated by transitions from the nondegenerate ^5^ *D*_0_ emitting level to the ^7^ *F*_*J*_ manifold.^2,4^

The ^5^ *D*_0_ → ^7^ *F*_1_ transition is predominantly magnetic-dipole allowed, whereas the ^5^ *D*_0_ → ^7^ *F*_2_ transition acquires forced electric-dipole character and is particularly sensitive to the chemical environment. The ratio

1 R2=I(D05→F27)I(D05→F17)

is consequently used extensively as an empirical descriptor of changes in Eu^3+^ coordination. Although this quantity is frequently called an “asymmetry ratio”, such terminology can be misleading if interpreted literally. The intensity of the hypersensitive transition depends not only on geometrical site symmetry but also on ligand polarizability, covalency, metal–ligand overlap, coordination geometry, and the electronic structure of the surrounding ligands.^2,4^ Consequently, *R*_2_ is more appropriately viewed as a multidimensional probe of the Eu^3+^ chemical environment than as a direct measure of symmetry alone.

The theoretical foundation for understanding the intensities of intraconfigurational rare-earth transitions was established independently by Judd and Ofelt.^5,6^ Within the Judd–Ofelt framework, the electric-dipole line strength between two 4*f* ^*N*^ states is expressed as

2 $$S_{\mathrm{ED}}=\sum_{\lambda =2,4,6}\Omega_{\lambda }{\left|\left\langle \psi J∥U^{\left(\lambda \right)}∥\psi^{\prime }J^{\prime }\right\rangle \right|}^{2}$$

Here, λ = 2, 4, 6 denotes the rank of the irreducible unit-tensor operator *U* ^(λ)^ entering the Judd–Ofelt expansion. The reduced matrix elements describe the lanthanide-ion electronic states, whereas the phenomenological intensity parameters Ω_λ_ incorporate the effect of the surrounding chemical environment. For Eu^3+^, the parameter Ω_2_ is particularly important because of its connection with the environmentally sensitive ^5^ *D*_0_ → ^7^ *F*_2_ transition.^2,3^

The microscopic interpretation of the Judd–Ofelt intensity parameters, however, is considerably richer than a simple measure of local symmetry. Forced electric-dipole intensity requires the admixture of electronic states of opposite parity into the nominal 4f^*N*^ manifold. In the original phenomenological picture, this occurs through odd-parity components of the crystal field that couple 4f^*N*^ states to opposite-parity configurations such as 4f^*N*–1^5d.^5,6^ Subsequent theoretical developments have emphasized additional mechanisms involving ligand polarization, metal–ligand overlap, and dynamic coupling.^7–11^

The hypersensitive character of selected rare-earth transitions therefore cannot, in general, be associated with a single static geometric variable. Judd already emphasized that hypersensitivity reflects particular properties of the ligand-field interaction rather than merely a generic reduction in site symmetry.^12^ Modern microscopic treatments reinforce this view by showing that the intensity parameters depend on both the electronic properties of the ligands and the symmetry-resolved couplings generated by the surrounding potential.^3,10,11^

This issue has received renewed attention with the development of a quantum extension of Judd–Ofelt theory in which the lanthanide ion and its environment are treated in a unified electronic description.^13^ In hydrated Eu^3+^, this approach highlights the role of static molecular dipoles in generating electric-dipole intensity and indicates that nitrate may influence the calculated Ω_2_ parameter even when the anion is not directly coordinated within the first hydration shell. Such results motivate a direct connection between the instantaneous molecular environment obtained from solution simulations and the electrostatic perturbations acting on the Eu^3+^ center.

A particularly appealing simplification is to characterize this environment through the magnitude of the static electric field, suggesting a relationship of the general form

3 $$\Omega_{2}\propto |E_{\mathrm{stat}}{|}^{2}$$

At a qualitative level, this hypothesis is intuitively attractive: a larger electrostatic perturbation might be expected to increase opposite-parity admixture and, hence, forced electric-dipole intensity. Moreover, the electric field provides a straightforward observable that can be extracted from molecular simulations and compared to different coordination environments.

Equation 3, however, involves a substantial reduction of the microscopic electrostatic problem. The potential generated by a molecular environment is intrinsically a spatially varying tensor quantity. Replacing it with |**E**|^2^ eliminates not only directional information but also the possibility that large contributions from different molecular subsystems cancel vectorially at the metal site. This distinction becomes particularly important in condensed phases, where local electric fields result from collective and fluctuating solvent configurations rather than from an isolated ligand.^14^

Hydrated Eu^3+^ provides a stringent test of such a scalar description. Trivalent lanthanide ions possess strongly structured hydration shells, with coordination numbers and water-exchange behavior that vary systematically across the series.^15–17^ The electric potential at Eu^3+^ therefore results from the combined contributions of directly coordinated water molecules, counterions, more distant solvent molecules, and their collective rearrangement. A nearby nitrate may generate a large direct contribution while inducing an opposing response from the hydration shell. Under such conditions, a nearly unchanged net field need not imply an unchanged local electrostatic environment.

Accurate simulation of this competition also requires an adequate description of the Eu^3+^–water interaction. Conventional 12–6 Lennard–Jones models can be problematic for highly charged metal ions because polarization effects are incorporated only implicitly.^18^ The 12–6–4 nonbonded model introduces an additional *r* ^–4^ contribution designed to represent ion-induced dipole interactions effectively and has been parametrized for highly charged metal ions in explicit water.^19^ More recent force-field developments have pursued transferable descriptions of lanthanide ions across aqueous and organic environments.^20^

For nitrate-containing solutions, the structural problem is especially relevant, because inner-sphere association cannot be inferred merely from anion proximity. Direct nitrate binding competes with the energetic cost of disrupting the highly organized Eu^3+^ hydration shell, and both inner- and outer-sphere species may participate in the solution speciation.^20,21^ Consequently, establishing a physically credible molecular picture of Eu^3+^ hydration and nitrate association is a prerequisite for assigning spectroscopic changes to a specific electrostatic mechanism.

A natural way to retain information discarded by eq 3 is to consider the spatial expansion of the electrostatic potential around the Eu^3+^ center. For a displacement **r** from the metal position

4 $$\Phi \left(r\right)=\Phi_{0}-E_{i}r_{i}-\frac{1}{2}G_{\mathrm{ij}}r_{i}r_{j}-\frac{1}{6}H_{\mathrm{ijk}}r_{i}r_{j}r_{k}+...$$

where *E*_*i*_ denotes the electric field, *G*_*ij*_ the field-gradient tensor, and *H*_*ijk*_ the next spatial derivative of the potential. Symmetrization and removal of the appropriate traces provide irreducible tensorial descriptors of successively higher rank. Whereas the first-rank field measures the net local electrostatic force per unit charge, higher spatial derivatives increasingly emphasize the local geometry and anisotropy of nearby charge distributions.

This hierarchy also has a direct symmetry significance. Under spatial inversion, an electrostatic multipole of rank *k* transforms according to (−1)^*k*^. Odd-rank components therefore possess the parity required to couple electronic configurations of opposite parity. Within a first-order perturbative treatment of the environmental electrostatic interaction, the rank-1 and rank-3 terms are therefore the odd-parity components of direct interest to the present analysis. This restriction should not be interpreted as a complete description at higher perturbative order: products of different tensor components may recouple into effective odd-rank channels; for example, a rank-1 ⊗ rank-2 contribution contains effective *K* = 1 and *K* = 3 components. Such higher-order mixed-rank contributions are not evaluated here and are not assumed to be negligible *a priori*.

The rank-3 contribution is consequently examined here as a first-order symmetry-resolved descriptor of the odd-parity part of the local electrostatic environment. Its scalar norm alone, however, still removes the angular information. A further decomposition into spherical or tesseral components is needed to determine whether two environments with similar total rank 3 strengths nevertheless possess distinct angular structures.

This observation suggests a more general question than whether nitrate simply strengthens the static field. Nitrate association could instead produce substantial rearrangements of the individual solvent and anion contributions while their resultant remains nearly unchanged. In such a scenario, the spectroscopically relevant signal would be encoded in *how* the electrostatic environment is assembled—through cancellation, spatial gradients, and angular components—rather than solely in the magnitude of the resulting field.

Despite the extensive literature on Eu^3+^ spectroscopy, Judd–Ofelt intensity mechanisms, and lanthanide hydration,^2,3,9,18^ these aspects are rarely examined simultaneously. In particular, a direct molecular-level comparison among the net electrostatic field, successive spatial derivatives of the potential, solvent–anion cancellation, and the angular decomposition of odd-rank components has not been established for hydrated Eu^3+^–nitrate speciation.

In the present work, we address this problem by combining molecular dynamics, free-energy sampling, periodic electrostatic calculations, irreducible tensor analysis, and multireference electronic-structure calculations. We first reassess the molecular structure of hydrated Eu^3+^–nitrate using a 12–6–4 description of Eu^3+^ and determine the free-energy profile for nitrate association. Conditional ensembles representative of inner-sphere, a broad interconversion region, outer-sphere, and far-window environments are then used to analyze the electrostatic perturbation acting at the Eu^3+^ center.

Rather than adopting eq 3 as a working molecular descriptor, we test its central microscopic premise: whether nitrate coordination produces a systematic increase in the resultant static electric-field magnitude. The periodic total electric field is decomposed into water and nitrate contributions to determine the extent of solvent–anion cancellation. The analysis is subsequently extended to the symmetric traceless rank-2 field-gradient tensor and to the odd-rank symmetric traceless rank-3 term. Finally, the rank-3 tensor is expressed in a Eu–N molecular frame and decomposed into angular-power components, allowing changes in the symmetry content of the local electrostatic environment to be distinguished from changes in its scalar magnitude.

Complementary CASSCF calculations are used to establish the Eu^3+^ 4f^6^ active space and the low-lying ^7^ *F* and ^5^ *D* manifolds. Strongly contracted NEVPT2 and spin–orbit coupling are subsequently employed to assess the energetic structure and spin mixing of these states. These electronic-structure calculations are used as an independent framework for interpreting the symmetry requirements of opposite-parity intensity mechanisms rather than as an empirical fit to the molecular-dynamics observables.

The central hypothesis tested in this work is therefore more stringent than the usual question of whether a coordinating anion produces a stronger electric field. We ask whether Eu^3+^–nitrate speciation is encoded primarily in the magnitude of the local field or in the *tensorial organization* of the surrounding electrostatic potential. As shown below, nitrate approach produces a strong, rank-dependent amplification of individual ligand contributions while the total scalar electrostatic magnitudes remain largely unchanged because of increasingly efficient solvent–anion cancellation. Coordination is instead resolved by the higher-rank tensorial structure and, in particular, by a pronounced redistribution of the angular components of the odd-rank local potential. These results provide a molecular-level basis for moving beyond |**E**|^2^ when characterizing Eu^3+^ coordination environments.

## Computational Methods

### Molecular Model and Force Field

All classical molecular dynamics (MD) simulations were performed with LAMMPS^22,23^ using periodic boundary conditions and real units. The simulated system contained one Eu^3+^ ion, three nitrate anions, and 500 explicit water molecules, yielding an overall electrically neutral simulation cell. Water was represented by the rigid four-site TIP4P-Ew model,^24^ using the published parameters without modification. Water geometry was constrained using the SHAKE algorithm.^25^

The Eu^3+^–water interaction was described using the 12–6–4 nonbonded model parametrized by Li, Song, and Merz for highly charged metal ions in TIP4P-Ew water.^19^ The general nonbonded interaction between Eu^3+^ and a site *j* was written as

5 $$U_{\mathrm{Eu},j}\left(r\right)=4\epsilon_{\mathrm{Eu},j}\left[{\left(\frac{\sigma_{\mathrm{Eu},j}}{r}\right)}^{12}-{\left(\frac{\sigma_{\mathrm{Eu},j}}{r}\right)}^{6}\right]-\frac{C_{4,\mathrm{Eu},j}}{r^{4}}+U_{\mathrm{Coul}}\left(r\right)$$

where the *r* ^–4^ contribution represents the ion-induced-dipole interaction effectively. The Eu^3+^–water Lennard–Jones and induction parameters were taken directly from the 12–6–4 parametrization of Li, Song, and Merz for TIP4P-Ew water^19^ and were used without modification. The published Eu^3+^–water induction coefficient

6 $$C_{4,\mathrm{Eu}-\mathrm{Ow}}=345.0\;\mathrm{kcal}\;{\mathrm{mol}}^{-1}\;{Å}^{4}$$

is reported explicitly here because it serves as the reference value for the Eu–nitrate induction-term transfer described below.

For nitrate, the partial charges and Lennard–Jones parameters were taken from the transferable lanthanide force-field framework of Duvail et al.^20^ and were used without modification.

Because a complete published Eu^3+^–nitrate 12–6–4 parametrization directly matched to the present TIP4P-Ew model was not available, the Eu–nitrate induction terms were generated by polarizability transfer from the published Eu–water coefficient. Specifically,

7 $$C_{4,\mathrm{Eu}-X}=C_{4,\mathrm{Eu}-\mathrm{Ow}}\frac{\alpha_{X}}{\alpha_{\mathrm{Ow}}}$$

using α_Ow_ = 1.444 Å^3^, α_N_ = 1.090 Å^3^, and α_O_ = 0.569 Å^3^. This procedure gives

8 $$\begin{array}{l} C_{4,\mathrm{Eu}-N}=260.4224\;\mathrm{kcal}\;{\mathrm{mol}}^{-1}\;{Å}^{4}\\ C_{4,\mathrm{Eu}-O_{NO_{3}}}=135.9453\;\mathrm{kcal}\;{\mathrm{mol}}^{-1}\;{Å}^{4} \end{array}$$

The nitrate intramolecular harmonic terms of the initial molecular topology were retained, with an equilibrium N–O distance of 1.250 Å and an equilibrium O–N–O angle of 120°. Thus, the Eu^3+^–nitrate model employed here should be regarded as a *hybrid transferable force field*, rather than as an exact reproduction of the complete force field of ref 20. This distinction is retained explicitly when interpreting quantitative association free energies.

The 12–6 and 12–6–4 contributions were implemented through the LAMMPS hybrid/overlay formalism, combining lj/cut/tip4p/long with tabulated −*C*_4_/*r* ^4^ contributions. A real-space cutoff of 10 Å was employed. Long-range electrostatic interactions were evaluated with the TIP4P-aware particle–particle particle–mesh (PPPM) method.

### Equilibration and Structural Characterization

After replacement of the initial nonbonded description by the 12–6–4/TIP4P-Ew model, the system was gently relaxed for 5 ps in the canonical ensemble at 298.15 K using a 0.25 fs integration time step. This short stabilization stage was introduced to avoid impulsive relaxation following the force-field replacement.

The integration time step was subsequently increased to 2 fs and the system was equilibrated for 100 ps in the isothermal–isobaric ensemble at

9 T=298.15K,⁣P=1atm

Nosé–Hoover temperature and isotropic pressure coupling times of 100 and 1000 fs, respectively, were employed. An additional 300 ps NPT trajectory was generated for structural screening, with coordinates stored every 1 ps. The PPPM relative accuracy during the structural and free-energy simulations was set to 10^–4^.

Eu–O_w_, Eu–O_NO_3__, and Eu–N_NO_3__ radial distribution functions were calculated from these trajectories. Water coordination was defined using *r*_Eu–O_w__ ≤ 2.80 Å, corresponding to the first hydration shell in the adopted model. The denticity of the selected nitrate was defined by counting nitrate oxygen atoms satisfying

10 $$r_{\mathrm{Eu}-O_{{\mathrm{NO}}_{3}}}\leq 3.20\;Å$$

These geometrical definitions were subsequently retained without change when constructing the conditional ensembles used for electrostatic analysis.

### Eu^3+^–Nitrate Potential of Mean Force

The free-energy landscape for nitrate association was determined using umbrella sampling along the Eu–N distance of one selected nitrate,

11 $$\xi =r_{\mathrm{Eu}-N}$$

The nitrate nitrogen, rather than an individual oxygen atom, was chosen as the reaction coordinate site so that mono- or bidentate coordination could emerge from the simulation rather than being imposed by the biasing coordinate.

A harmonic umbrella potential

12 $$U_{i}^{\mathrm{bias}}\left(\xi \right)=\frac{1}{2}k_{\mathrm{umb}}{\left(\xi -\xi_{i}\right)}^{2}$$

was used with

13 $$k_{\mathrm{umb}}=20\;\mathrm{kcal}\;{\mathrm{mol}}^{-1}\;{Å}^{-2}$$

Umbrella centers were separated by 0.25 Å. The biased trajectories were propagated in the NVT ensemble at 298.15 K with a 2 fs time step, retaining the periodic box associated with the equilibrated source configuration. Forward and reverse branches were propagated separately with independently randomized velocities to probe path dependence and incomplete relaxation. They should not, however, be interpreted as independent configurational replicates: several later branches were seeded from configurations extracted from earlier umbrella trajectories and thus retain shared configurational ancestry.

The production PMF combined the converged portions of several overlapping sets of umbrella windows, summarized in Table 1. The genealogies of these branches were tracked explicitly. Temporal block bootstrap therefore quantifies sampling uncertainty within the retained logical trajectories but does not, by itself, remove uncertainty associated with their shared ancestry; a separate parent/child genealogy sensitivity analysis was used to assess that issue.

**Table 1:** Sampling Retained in the Final Eu–N Potential of Mean Force^a^

Eu–N range (Å)	Initialization	Retained sampling/window
3.50–5.00	separately propagated forward/reverse branches	150 ps
3.50–4.25	nearest-*r* reseeded forward/reverse branches	250 ps
5.25–6.75	forward-derived extension	40 ps
5.25–6.00	reverse-derived extension	80 ps
7.00–9.00	staged forward-derived windows	150 ps

a The listed times correspond to the production interval retained after discarding the initial relaxation portion of each umbrella trajectory.

The biased distributions were combined with the weighted histogram analysis method (WHAM).^26^ A bin width of 0.05 Å was used over 3.20 ≤ *r* ≤ 9.30 Å. Because the reaction coordinate is a three-dimensional radial distance, the raw distance free energy was corrected for the radial Jacobian according to

14 $$W\left(r\right)=F_{\mathrm{dist}}\left(r\right)+2k_{B}T\ln r$$

The arbitrary free-energy zero was defined by imposing

15 $${\left\langle W\left(r\right)\right\rangle }_{7.5\leq r\leq 9.0Å}=0$$

Statistical uncertainty in the production PMF was estimated by 500 temporal block bootstrap reconstructions. Within each logical umbrella trajectory, the production data were divided into 10 ps blocks, and these blocks were resampled with replacement before repeating the complete WHAM reconstruction.

The large-distance PMF retains a weak residual slope associated with the finite periodic cell. Accordingly, the long-distance reference is used only to define an arbitrary free-energy zero. Quantitative mechanistic interpretation is restricted to locally converged free-energy differences, such as the inner-to-outer difference and the free-energy rises from each local minimum to the intervening barrier-like maximum along *r*_Eu–N_. No outer-to-far difference is interpreted as a standard association free energy, and the long-distance tail is not claimed to be a rigorously converged asymptotic bulk plateau.

### Sensitivity to the Transferred Eu–Nitrate *C*_4_ Terms

Because the Eu–nitrate *C*_4_ coefficients were obtained by transfer rather than by a direct fit for the present Eu^3+^/TIP4P-Ew/nitrate combination, two complementary stress tests were carried out. The published Eu–O_w_ coefficient was kept fixed throughout.

First, the nitrate induction coefficients were perturbed at fixed sampled geometries. Coherent scale factors λ = 0.8, 0.9, 1.0, 1.1, 1.2 were applied simultaneously to *C*_4,Eu–N_ and $C_{4,\mathrm{Eu}-O_{{\mathrm{NO}}_{3}}}$ , and additional ±20% one-at-a-time perturbations were evaluated for the N and O terms. Paired free-energy shifts were obtained from the corresponding changes in the −*C*_4_/*r* ^4^ energy and propagated with temporal-block resampling. The sparse configurations used for this perturbative analysis were not interpreted as independent production PMFs; only paired changes relative to the dense production PMF were used.

Second, configurational sensitivity of the rank-3 angular signature was probed by explicit matched MD branches at coherent λ = 0.8, 1.0, 1.2. For each source configuration, the same newly randomized velocity seed was used across the three λ values, so that the branches were matched across Hamiltonians while retaining the shared configurational ancestry of their parent umbrella sources. Each branch was propagated for 200 ps at 298.15 K; the first 50 ps were discarded, and the final 150 ps were used for conditional selection. The historical interconversion selector, 3.85 ≤ *r*_Eu–N_ ≤ 4.30 Å with no water-coordination or denticity restriction, yielded 70, 58, and 40 configurations for λ = 0.8, 1.0, 1.2, respectively. The corresponding far ensembles were source-balanced to 119 configurations at each λ.

The total electrostatic rank-3 tensor for these configurations was recomputed at <i>h</i> = 0.04 Å with PPPM/TIP4P accuracy 10^–7^ and no LJ, *C*_4_, umbrella, or dynamics terms in the frozen-geometry rerun. Thus, *C*_4_ affects *p*_*q*_ only indirectly through the configurations generated by MD, not through the electrostatic rerun at fixed geometry. The predeclared control was the sign of Δ*p*_0_ = *p*_0_^interconversion^ – *p*_0_^far^ at λ = 1.0. Primary uncertainty estimates used equal source weights and temporal blocks of 5000 MD steps (approximately 10 ps), with 20 000 bootstrap replicates; source-resampling and leave-one-source-out analyses were used as complementary robustness checks. Because the interconversion samples did not meet the conservative target of 120 configurations per Hamiltonian, this calculation is treated as a targeted configurational-robustness diagnostic rather than as a new production characterization of the conditional state.

### Conditional Coordination Ensembles

Rather than averaging the electrostatic observables over the complete biased data set, structurally defined conditional ensembles were constructed to compare distinct regions of the Eu^3+^–nitrate association coordinate. Four conditional states were defined from three geometrical descriptors: the Eu–N distance, the water coordination number *N*_w_, and the denticity *d*_NO_3__ of the selected nitrate. Their definitions are summarized in Table 2.

**Table 2:** Geometrical Definitions of the Conditional Ensembles Used in the Electrostatic Analysis

State	*r*_Eu–N_ (Å)	*N*_w_	*d*_NO_3__
inner	3.55–3.80	8	1
interconversion	3.85–4.30	unrestricted	unrestricted
outer	5.35–5.80	9	0
far	7.50–9.00	9	0

Here, *N*_w_ was evaluated with the 2.80 Å Eu–O_w_ cutoff and *d*_NO_3__ with the 3.20 Å Eu–O_NO_3__ cutoff defined above. The final electrostatic data set contained 120 snapshots per state. Snapshots were distributed as evenly as possible among the available source trajectory segments rather than being selected exclusively from a single umbrella window. The inner, interconversion, outer, and far ensembles contained configurations originating from 8, 12, 2, and 7 source identifiers, respectively. These source identifiers are used as clustering units in the statistical analysis; because some umbrella branches retain shared configurational ancestry, they are not described as fully independent replicates.

These ensembles are deliberately *conditional structural ensembles*. They are not equilibrium populations and are not assigned equal thermodynamic weights. Their purpose is to determine how the electrostatic environment changes when a configuration satisfies a particular coordination condition. Likewise, the term “far” denotes a far-window conditional reference and not an infinite-dilution bulk state.

### Periodic Electrostatic Field and Component Decomposition

For each of the 480 selected structures, the electrostatic field at the Eu^3+^ position was recalculated under the same periodic boundary conditions as those for the MD simulation. These calculations were performed as frozen-geometry LAMMPS reruns in which LJ, *C*_4_/*r* ^4^, umbrella, thermostat, and integration forces were removed. Only Coulombic interactions were retained.

The lj/cut/tip4p/long infrastructure was preserved with zero LJ well depths rather than replaced by a generic point-charge Coulomb potential. This procedure ensures that the negative TIP4P-Ew water charge remains located on its implicit M site. Long-range Coulomb interactions were recalculated with PPPM/TIP4P using a tightened accuracy of 10^–5^.

The field was obtained from the Coulombic force on the Eu^3+^ probe

16 $$E=\frac{F_{\mathrm{Eu}}}{q_{\mathrm{Eu}}}$$

In LAMMPS real units, this becomes

17 $$E_{i}\left(V{Å}^{-1}\right)=\frac{F_{\mathrm{Eu},i}}{3\times 23.060548}$$

where *F*_Eu,*i*_ is expressed in kcal mol^–1^ Å^–1^ and the factor 3 is the Eu^3+^ charge.

A charge-mask rerun protocol was used to separate the periodic field into molecular contributions without changing the atomic geometry. Four electrostatic calculations were performed for every snapshot

18 $$\begin{array}{l} E_{\mathrm{tot}}:\;\mathrm{all}\;\mathrm{charges}\;\mathrm{active}\\ E_{{\mathrm{NO}}_{3}}:\;\mathrm{water}\;\mathrm{charges}\;\mathrm{set}\;\mathrm{to}\;\mathrm{zero}\\ E_{\mathrm{sel}}:\;\mathrm{water}\;\mathrm{and}\;\mathrm{nonselected}‐\mathrm{nitrate}\;\mathrm{charges}\;\mathrm{set}\;\mathrm{to}\;\mathrm{zero}\\ E_{w,\mathrm{direct}}:\;\mathrm{all}\;\mathrm{nitrate}\;\mathrm{charges}\;\mathrm{set}\;\mathrm{to}\;\mathrm{zero} \end{array}$$

The water contribution was independently reconstructed as

19 $$E_{w,\mathrm{diff}}=E_{\mathrm{tot}}-E_{{\mathrm{NO}}_{3}}$$

and agreement between **E**_w,direct_ and **E**_w,diff_ provided an internal linearity check.

To quantify destructive interference between the water and nitrate fields, a first-rank cancellation index was defined as

20 $$C_{1}=1-\frac{∥E_{\mathrm{tot}}∥}{∥E_{w}∥+∥E_{{\mathrm{NO}}_{3}}∥}$$

Thus, *C*_1_ = 0 corresponds to no reduction of the resultant magnitude, whereas values approaching unity indicate a strong cancellation. The relative orientation of the water and nitrate components was additionally quantified through the vector cosine.

### Rank-2 Field-Gradient Tensor

The spatial variation of the electric field was characterized by the Cartesian field-gradient tensor

21 $$G_{\mathrm{ij}}=\frac{\partial E_{i}}{\partial x_{j}}$$

For each frozen environmental configuration, the Eu probe was displaced along the three Cartesian directions while all solvent and nitrate coordinates were kept fixed. Central finite differences were evaluated as

22 $$G_{\mathrm{ij}}≃\frac{E_{i}\left(r_{\mathrm{Eu}}+\delta e_{j}\right)-E_{i}\left(r_{\mathrm{Eu}}-\delta e_{j}\right)}{2\delta }$$

Three displacements were tested,

23 δ=0.01,0.02,0.04Å

and δ = 0.02 Å was adopted for the production analysis after numerical convergence tests. Because finite differentiation amplifies noise in the underlying electric field, the PPPM accuracy was tightened further to 10^–6^.

The physical rank-2 descriptor was taken as the symmetric traceless (STF) part of the gradient

24 $$T^{\left(2\right)}=\frac{1}{2}\left(G+G^{T}\right)-\frac{\mathrm{Tr}\left[\left(G+G^{T}\right)/2\right]}{3}I$$

Its scalar magnitude was quantified by the Frobenius norm,

25 $${∥T^{\left(2\right)}∥}_{F}={\left[\sum_{\mathrm{ij}}{\left(T_{\mathrm{ij}}^{\left(2\right)}\right)}^{2}\right]}^{1/2}$$

The same charge-mask decomposition used at rank 1 was repeated for the rank-2 tensor. A rank-2 cancellation index was consequently defined as

26 $$C_{2}=1-\frac{{∥T_{\mathrm{tot}}^{\left(2\right)}∥}_{F}}{{∥T_{w}^{\left(2\right)}∥}_{F}+{∥T_{{\mathrm{NO}}_{3}}^{\left(2\right)}∥}_{F}}$$

The projection along the instantaneous Eu–N axis,

27 $$T_{\mathrm{EuN}}={\mathrm{r̂}}_{\mathrm{EuN}}^{T}T^{\left(2\right)}{\mathrm{r̂}}_{\mathrm{EuN}}$$

and the alignment of the principal tensor axis with **r̂**_EuN_ were also monitored.

Numerical validation included the antisymmetric residual of G , the trace of the electrostatic gradient, convergence among the three displacement magnitudes, and agreement between the directly computed water tensor and $T_{\mathrm{tot}}^{\left(2\right)}-T_{{\mathrm{NO}}_{3}}^{\left(2\right)}$ . The complete convergence diagnostics are reported in the Supporting Information.

### Odd-Parity Rank-3 Electrostatic Tensor

To probe higher-order and parity-sensitive structure in the local electrostatic environment, the analysis was extended to the second spatial derivative of the electric field

28 $$H_{\mathrm{ijk}}=\frac{\partial^{2}E_{i}}{\partial x_{j}\partial x_{k}}$$

For repeated derivative directions

29 $$H_{\mathrm{ijj}}≃\frac{E_{i}\left(+he_{j}\right)-2E_{i}\left(0\right)+E_{i}\left(-he_{j}\right)}{h^{2}}$$

whereas mixed derivatives were obtained from

30 $$H_{\mathrm{ijk}}≃\frac{1}{4h^{2}}\left[E_{i}\left(+he_{j},+he_{k}\right)-E_{i}\left(+he_{j},-he_{k}\right)-E_{i}\left(-he_{j},+he_{k}\right)+E_{i}\left(-he_{j},-he_{k}\right)\right],j\neq k$$

Finite-difference steps of

31 h=0.03,⁡0.04,⁡0.06Å

were evaluated, with *h* = 0.04 Å adopted for production. The electrostatic PPPM accuracy was tightened to 10^–7^ for these calculations. The raw third-rank tensor was fully symmetrized over Cartesian index permutations to give $S^{\left(3\right)}$ . Its trace vector was defined as

32 $$v_{k}=\sum_{i}S_{\mathrm{iik}}^{\left(3\right)}$$

and the rank-3 symmetric traceless tensor was constructed as

33 $$O_{\mathrm{ijk}}^{\left(3\right)}=S_{\mathrm{ijk}}^{\left(3\right)}-\frac{1}{5}\left(\delta_{\mathrm{ij}}v_{k}+\delta_{\mathrm{ik}}v_{j}+\delta_{\mathrm{jk}}v_{i}\right)$$

With the sign convention adopted for the Taylor expansion of the electrostatic potential, the cubic coefficient was defined as

34 $$A_{\mathrm{ijk}}^{\left(3\right)}=-\frac{1}{6}O_{\mathrm{ijk}}^{\left(3\right)}$$

The quantity $A^{\left(3\right)}$ is therefore an odd-rank coefficient of the local electrostatic potential; it is not a Judd–Ofelt parameter. Its scalar magnitude was evaluated from the Cartesian Frobenius norm. As for ranks 1 and 2, total, water, all-nitrate, and selected-nitrate components were calculated separately. The corresponding cancellation index was

35 $$C_{3}=1-\frac{{∥A_{\mathrm{tot}}^{\left(3\right)}∥}_{F}}{{∥A_{w}^{\left(3\right)}∥}_{F}+{∥A_{{\mathrm{NO}}_{3}}^{\left(3\right)}∥}_{F}}$$

Symmetry residuals, trace removal, water–nitrate additivity, and finite-difference convergence among the three values of *h* were used as numerical diagnostics. Their complete distributions are given in the Supporting Information.

### Molecular-Frame Decomposition of the Rank-3 Tensor

The Frobenius norm of $A^{\left(3\right)}$ contains no information about how the odd-rank electrostatic power is distributed angularly. Consequently, each total rank-3 tensor was transformed to an instantaneous molecular frame in which

36 $$ẑ={\mathrm{r̂}}_{\mathrm{Eu}\to N}$$

where N is the nitrogen atom of the selected nitrate. The Cartesian STF tensor was projected onto an orthonormal real tesseral rank-3 basis containing the seven components

37 $$q_{0},\;q_{1c},\;q_{1s},\;q_{2c},\;q_{2s},\;q_{3c},\;q_{3s}$$

The corresponding grouped angular-power fractions were defined as

38 $$p_{0}=\frac{q_{0}^{2}}{{∥A^{\left(3\right)}∥}_{F}^{2}}$$

39 $$p_{1}=\frac{q_{1c}^{2}+q_{1s}^{2}}{{∥A^{\left(3\right)}∥}_{F}^{2}}$$

40 $$p_{2}=\frac{q_{2c}^{2}+q_{2s}^{2}}{{∥A^{\left(3\right)}∥}_{F}^{2}}$$

and

41 $$p_{3}=\frac{q_{3c}^{2}+q_{3s}^{2}}{{∥A^{\left(3\right)}∥}_{F}^{2}}$$

such that

42 $$p_{0}+p_{1}+p_{2}+p_{3}=1$$

Because the sine and cosine components belonging to a given |*q*| are combined quadratically, each *p*_|*q*|_ is invariant to the arbitrary choice of the *x* and *y* axes under rotation about the molecular Eu–N axis. This gauge invariance was explicitly verified numerically. The complete Cartesian definitions of the orthonormal tesseral basis are provided in the Supporting Information.

### Statistical Analysis of the Conditional Ensembles

Statistical comparisons among the electrostatic observables were performed directly on the 120 snapshots retained for each conditional state. Because snapshots originating from the same umbrella/source trajectory may retain temporal correlation, two complementary bootstrap schemes were evaluated. The historical “transition” label used during data generation corresponds throughout this manuscript to the broad 3.85–4.30 Å interconversion-region conditional ensemble; it is not identified with a dynamical transition state.

An ordinary independent-and-identically distributed (IID) bootstrap resampled individual snapshots. A more conservative *source-cluster bootstrap* instead treated the complete source_id trajectory segment as the resampling unit, so that all snapshots associated with a selected source remained grouped during resampling. The latter was adopted as the primary uncertainty estimate reported in the main text. This procedure captures between-source heterogeneity while preserving the dependence among snapshots belonging to the same source. It does not independently resample temporal structure within a source and should not be interpreted as eliminating shared genealogical ancestry among source branches. For each contrast, 20,000 bootstrap replicates were generated and percentile-based 95% confidence intervals were calculated. The principal comparisons were

43 inner−far

and

44 interconversion−far

because they directly test the mechanistic questions of stable inner-sphere association and sampling of the broad interconversion region. The outer ensemble was retained in the descriptive state-resolved analysis, but its source-cluster uncertainty was interpreted cautiously because it was represented by only two distinct source identifiers.

Standardized effects were quantified using the unpaired pooled-standard-deviation Cohen effect size

45 $$d=\frac{{\mathrm{x̅}}_{A}-{\mathrm{x̅}}_{B}}{s_{p}}$$

with

46 $$s_{p}^{2}=\frac{\left(n_{A}-1\right)s_{A}^{2}+\left(n_{B}-1\right)s_{B}^{2}}{n_{A}+n_{B}-2}$$

The interpretation therefore emphasizes effect magnitudes and bootstrap confidence intervals rather than binary significance tests. No equilibrium weighting among the inner, interconversion, outer, and far ensembles was applied: all reported state contrasts answer how the *conditional distributions* differ and do not answer how frequently those states occur at equilibrium.

### QM-Cluster Construction and Periodic Electrostatic Embedding

A subset of the classical configurations was used to establish a quantum-mechanical description of the Eu^3+^ electronic manifold and to assess whether the periodic electrostatic environment could be represented adequately in finite-embedded-cluster calculations. An initial mechanistic design set contained eight representative configurations from each conditional state for a total of 32 structures. The selection sampled both central configurations and the variation in the rank 3 angular descriptors rather than attempting to reproduce an equilibrium statistical average.

For each configuration, the quantum-mechanical region contained

47 $${\mathrm{Eu}}^{3+}+{\mathrm{NO}}_{3\mathrm{selected}}^{-}+N_{w}H_{2}O$$

where the *N*_w_ nearest *complete* water molecules were retained. The selected nitrate was included as a complete molecule in every state, and the formal charge of the quantum region was, therefore, + 2. All cluster geometries were taken directly from the MD snapshots; no geometry optimization was performed. The remainder of the central simulation cell was represented initially by a full point-charge embedding. The two nonselected nitrate anions retained their classical charges *q*_N_ = +0.9934*e* and *q*_O_ = −0.6645e. External TIP4P-Ew waters were represented by the two H-site charges and the negative M-site charge; no charge was placed on the TIP4P oxygen LJ site.

A finite central-cell embedding cannot reproduce exactly the low-rank electrostatic field of an infinite periodic Ewald/PPPM system. Consequently, the full point-charge embedding was augmented by a compact neutral auxiliary shell designed to restore the missing periodic rank-1 and rank-2 contributions without modifying the already well-reproduced local rank-3 field. Twenty-six fixed auxiliary sites were placed on a sphere of radius 15 Å around Eu^3+^. Their charges were obtained from the minimum-*L*_2_ solution satisfying

48 $$\sum_{a}q_{a}=0$$

49 $$E_{\mathrm{aux}}=E_{\mathrm{PPPM}}-E_{\mathrm{FULL}}$$

and

50 $$T_{\mathrm{aux}}^{\left(2\right)}=T_{\mathrm{PPPM}}^{\left(2\right)}-T_{\mathrm{FULL}}^{\left(2\right)}$$

while imposing

51 $$A_{\mathrm{aux}}^{\left(3\right)}=0$$

Thus, the auxiliary shell repairs the missing periodic homogeneous field and field gradient but does not introduce an artificial rank 3 perturbation. The resulting embedding is denoted as FULL+AUX. Its electrostatic fidelity was verified for the full 32-configuration design set before any electronic structure interpretation was attempted. Details of the finite-embedding convergence tests and auxiliary-charge diagnostics are given in the Supporting Information.

### Multireference Electronic-Structure Calculations

The multireference calculations reported in the main text were performed with OpenMolcas v26.06.^27^ A representative central configuration from the inner conditional ensemble was used as the spectroscopic benchmark. The quantum cluster contained Eu^3+^, the selected nitrate, and eight complete first-shell water molecules, corresponding to 29 atoms, formal charge +2, and 172 electrons. The calculation was performed directly on the MD geometry using the validated FULL+AUX electrostatic embedding described above. ANO-RCC basis sets were employed throughout. H, N, and O were described at the ANO-RCC-VDZP level,^28^ whereas Eu was described using ANO-RCC-VTZP.^29^ Scalar relativistic effects were included with the Douglas–Kroll–Hess Hamiltonian,^30,31^ and two-electron integrals were handled using Cholesky decomposition. Molecular symmetry was disabled (*C*_1_) to avoid imposing symmetry constraints inherited neither from the instantaneous solution geometry nor from the embedding.

The Eu^3+^ 4f^6^ manifold was described with a CAS­(6,7) active space containing the six 4*f* electrons distributed among seven predominantly Eu-4*f* orbitals

52 $$\mathrm{CAS}\left(6,7\right)={\left(4f\right)}^{6}$$

The remaining 166 electrons occupied 83 inactive orbitals. Equal-weight state-averaged CASSCF calculations were carried out independently for seven septet roots and five quintet roots. The seven septet states span the spin-free parent space required to construct the ^7^ *F*_*J*_ manifold, whereas the five lowest quintet roots provide the corresponding spin-free ^5^ *D* parent spaces.

Dynamic correlation was added through state-specific strongly contracted *N*-electron valence-state second-order perturbation theory (SC-NEVPT2).^32^ Separate SC-NEVPT2 corrections were calculated for all seven septet and five quintet roots. Spin–orbit coupling was subsequently introduced with the restricted active-space state-interaction (RASSI) approach using atomic mean-field-integral (AMFI) spin–orbit matrix elements.^33^ The interaction space therefore contained the seven septet and five quintet spin-free CASSCF states, generating 74 spin–orbit states.

To incorporate the differential dynamic-correlation correction while retaining the CASSCF/RASSI interstate matrix elements, the diagonal spin-free Hamiltonian energies entering RASSI were replaced by their corresponding state-specific SC-NEVPT2 energies. The resulting treatment is referred to here as a *diagonal-energy-corrected SC-NEVPT2/RASSI-SO* calculation. It should not be interpreted as a fully correlated spin–orbit wave function optimization. As a sensitivity check, quasi-degenerate SC-NEVPT2 calculations were also performed separately for the septet and quintet manifolds. The resulting corrections to the relative spin-free energies were small compared with the effects discussed in the main text; the complete comparison is provided in the Supporting Information.

The CAS­(6,7) space contains only the Eu-4*f* shell and therefore cannot represent explicit opposite-parity configurations such as

53 $$4f^{6}↔4f^{5}5d^{1}$$

An exploratory generalized-active-space calculation containing seven 4*f* and five Eu-*d*-like orbitals was consequently used only to assess the numerical accessibility of the 4f^5^5d^1^ sector. A balanced five-orbital d shell could not be stabilized under the state averaging required for the present embedded structure. The calculation was therefore not used to obtain transition energies, oscillator strengths, Judd–Ofelt parameters, or any other quantitative observable reported in this work. Hence, the multireference calculations are used here to establish the 4f^6^ manifold, its dynamic-correlation corrections, and spin–orbit mixing. They do not constitute a direct calculation of the forced electric-dipole intensity. The connection between the classically determined odd-rank electrostatic descriptors and explicit 4f^6^–opposite-parity intensity borrowing is therefore interpreted as a mechanistic hypothesis constrained by symmetry, rather than as a quantitatively fitted Judd–Ofelt mapping.

## Results and Discussion

### Hydration Structure and Nitrate-Association Free Energy

A prerequisite for interpreting the electrostatic response of hydrated Eu^3+^–nitrate complexes is a physically reliable description of the primary coordination environment. Figure 1a shows the Eu–O_w_ radial distribution function obtained from the unbiased nitrate-containing trajectory using the final 12–6–4/TIP4P-Ew description. The first hydration peak occurs at

54 $$r_{\mathrm{peak}}^{\mathrm{Eu}-O_{w}}=2.450Å$$

and integration to the 2.80 Å first-shell cutoff gives

55 $$N_{w}\left(2.80\;Å\right)=8.997$$

demonstrating an essentially nine-coordinate aqueous Eu^3+^ environment. This result is consistent with the established high-coordination character of hydrated Eu^3+^ and with the structural behavior expected from 12–6–4 ion models.^19,20^

**Figure 1:**
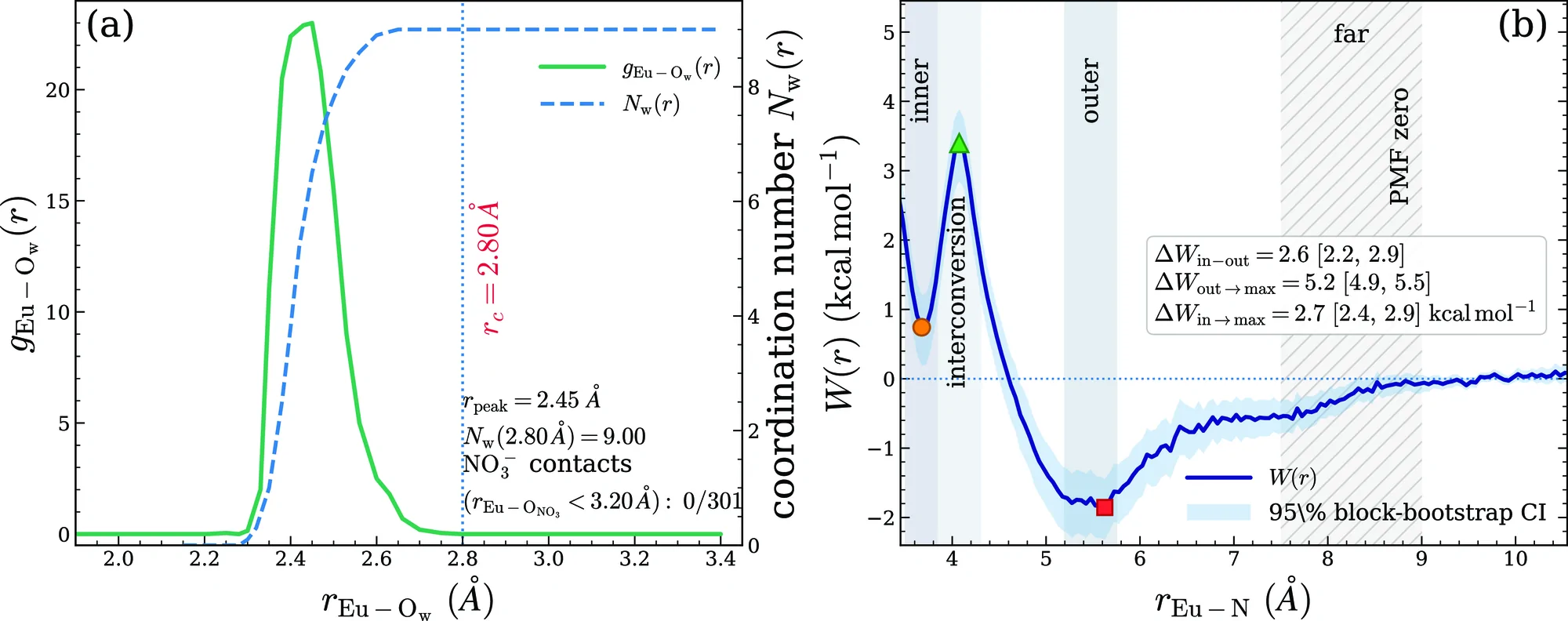
Structural hydration environment of Eu^3+^ and tagged-nitrate potential of mean force. (a) Eu–water radial distribution function, *g*_Eu–O_w__(*r*), together with the cumulative water coordination number, *N*_*w*_(*r*), obtained from the unbiased nitrate-containing production trajectory. The first-shell maximum occurs at *r*_peak_ = 2.45 Å, while integration up to *r*_*c*_ = 2.80 Å yields *N*_*w*_ = 9.00, consistent with an approximately nine-coordinate first hydration shell. No direct inner-sphere nitrate contact was detected in this unbiased structural ensemble: none of the 301 analyzed frames exhibited $r_{\mathrm{Eu}-O_{{\mathrm{NO}}_{3}}}<3.20$ Å. (b) One-dimensional tagged-nitrate potential of mean force, *W*(*r*_Eu–N_), from the final umbrella-sampling analysis. The solid curve denotes the PMF and the shaded envelope the 95% temporal block-bootstrap confidence interval. The local PMF topology comprises an inner-sphere minimum at *r*_Eu–N_ = 3.675 Å, a barrier-like maximum at 4.075 Å, and an outer-sphere minimum at 5.575 Å. The corresponding local free-energy differences are *W*_in_ – *W*_out_ = 2.6 kcal mol^–1^ [95% CI: 2.2–2.9 kcal mol^–1^], Δ*W*_out→max_ = 5.2 kcal mol^–1^ [95% CI: 4.9–5.5 kcal mol^–1^], and Δ*W*_in→max_ = 2.7 kcal mol^–1^ [95% CI: 2.4–2.9 kcal mol^–1^]. The shaded state windows identify the historical interconversion conditional ensemble (3.85 ≤ *r*_Eu–N_ ≤ 4.30 Å), the outer conditional ensemble, and the far conditional ensemble. The hatched 7.50–9.00 Å interval is also the operational region used to define the PMF zero. This far-window reference is a numerical convention for the one-dimensional PMF and is not interpreted as an infinite-dilution bulk state, a rigorously converged asymptotic plateau, or a standard-state association free energy.

Importantly, no spontaneous nitrate penetration into the primary coordination shell was observed during the unbiased structural trajectory. Across the 301 analyzed configurations, none displayed an Eu–O_NO_3__ separation below the 3.20 Å criterion used to identify direct nitrate coordination. The smallest observed Eu–O_NO_3__ distance was 3.953 Å, whereas the mean nearest Eu–O_NO_3__ separation was 6.491 Å. Thus, under unbiased sampling on this time scale, the hydrated ion retains its primary water coordination shell and nitrate remains predominantly outside it.

Absence of spontaneous inner-sphere association in a finite unbiased trajectory cannot, however, distinguish thermodynamic preference from kinetic trapping. We therefore determined the potential of mean force along the Eu–N distance of a selected nitrate ion. The Jacobian-corrected profile and its temporal block-bootstrap uncertainty are shown in Figure 1b. Three locally distinct features are resolved: an inner-sphere minimum at

56 $$r_{\mathrm{inner}}=3.675\;Å$$

a barrier-like free-energy maximum along the Eu–N coordinate at

57 $$r_{\max}=4.075\;Å$$

and an outer-sphere minimum at

58 $$r_{\mathrm{outer}}=5.575\;Å$$

Within the locally converged portion of the PMF, the inner-sphere minimum lies above the outer-sphere basin by

59 $$\Delta W_{\mathrm{inner}-\mathrm{out}}=2.6\;\mathrm{kcal}\;{\mathrm{mol}}^{-1}$$

with a 95% temporal block-bootstrap interval of [2.2, 2.9] kcal mol^–1^. The free-energy rise from the outer-sphere basin to the intervening maximum is

60 $$\Delta W_{\mathrm{out}\to \max}=5.2\;\mathrm{kcal}\;{\mathrm{mol}}^{-1}$$

with a 95% interval of [4.9, 5.5] kcal mol^–1^. From the inner-sphere minimum, the corresponding rise is

61 $$\Delta W_{\mathrm{in}\to \max}=2.7\;\mathrm{kcal}\;{\mathrm{mol}}^{-1}$$

with a 95% interval of [2.4, 2.9] kcal mol^–1^. These quantities are one-dimensional free-energy differences along *r*_Eu–N_; the intervening maximum is therefore described as barrier-like rather than as a uniquely identified dynamical transition state.

The local topology is also insensitive to the shared ancestry of the umbrella branches. Restricting the analysis to parent-only or to child-only/reseeded branch subsets preserved the positions and ordering of the inner minimum, intervening maximum, and outer minimum, with the inner–outer contrast spanning approximately 2.47–2.66 kcal mol^–1^. This genealogy test is distinct from the temporal bootstrap and is used only as a structural robustness check.

These results establish that nitrate association in the present model is not described by a persistent inner-sphere complex. Rather, the outer-sphere basin is locally favored, while inner-sphere configurations form a higher-free-energy metastable basin. Nitrate approach must therefore be considered together with reorganization and partial disruption of the strongly coordinated Eu^3+^ hydration environment. A targeted sensitivity analysis confirms that this local thermodynamic ordering does not depend on fine-tuning of the transferred nitrate induction coefficients. When both transferred Eu–nitrate *C*_4_ terms are scaled coherently by λ = 0.8, 0.9, 1.0, 1.1, 1.2 while the published Eu–water *C*_4_ is kept fixed, the propagated inner–outer differences are 3.517, 3.046, 2.575, 2.104, and 1.635 kcal mol^–1^, respectively. The corresponding 95% intervals remain wholly positive even at the two extremes: [3.141, 3.890] kcal mol^–1^ for λ = 0.8 and [1.278, 1.993] kcal mol^–1^ for λ = 1.2. Across the coherent stress test, the local slope is dΔ*W*_inner–out_/dλ = −4.70 kcal mol^–1^ (95% CI [−4.86, −4.47]). One-at-a-time perturbations further indicate that the Eu–O_NO_3__ induction term accounts for roughly 78% of this sensitivity and the Eu–N term for roughly 22%. Thus, the *magnitude* of the inner–outer free-energy separation is sensitive to the transferred *C*_4_ terms, but its sign and the local basin ordering remain robust throughout this ± 20% diagnostic range.

### Nitrate Amplification is Masked by Solvent–Anion Cancellation

The original mechanistic picture assumed that nitrate coordination primarily modifies Eu^3+^ luminescence by increasing the magnitude of the local static electric field. A direct periodic calculation of the electrostatic field at the Eu^3+^ site does not support this picture. For each conditional ensemble, the total electrostatic field was evaluated using the periodic particle–particle particle–mesh electrostatic description, including the complete water and nitrate environment. The mean field magnitudes are

62 $$\begin{array}{l} \left\langle \right|E|\rangle_{\mathrm{inner}}=0.2414\;V\;{Å}^{-1}\\ \left\langle \right|E|\rangle_{\mathrm{interconversion}}=0.2422\;V\;{Å}^{-1}\\ \left\langle \right|E|\rangle_{\mathrm{outer}}=0.2372\;V\;{Å}^{-1}\\ \left\langle \right|E|\rangle_{\mathrm{far}}=0.2392\;V\;{Å}^{-1} \end{array}$$

Thus, despite the large structural change associated with nitrate approach, the total field at Eu^3+^ remains essentially invariant. For the inner-minus-far contrast,

63 $$\Delta \left|E\right|=0.00216\;V\;{Å}^{-1}$$

with a source-cluster bootstrap 95% confidence interval of

64 $$\left[-0.01593,\;+0.02363\right]\;V\;{Å}^{-1}$$

and a standardized effect size of only

65 d=0.020

The interconversion-minus-far difference is similarly negligible (*d* = 0.029). These results demonstrate that coordination state is not encoded in the magnitude of the total first-rank electrostatic field. This near invariance does *not* imply that nitrate has little electrostatic effect. On the contrary, the magnitude of the nitrate component increases strongly as the ion approaches Eu^3+^

66 $$\frac{\left\langle \right|E_{{\mathrm{NO}}_{3}}|\rangle_{\mathrm{inner}}}{\left\langle \right|E_{{\mathrm{NO}}_{3}}|\rangle_{\mathrm{far}}}=4.52$$

with a source-cluster bootstrap 95% confidence interval of [3.42, 6.39]. The apparent paradox between a strongly increasing nitrate contribution and an almost invariant total field is resolved by examining the vectorial decomposition of the electrostatic environment. The water and nitrate fields become increasingly antiparallel upon nitrate approach. We quantify this compensation through the cancellation index

67 $$C_{1}=1-\frac{\left|E_{\mathrm{tot}}\right|}{\left|E_{\mathrm{water}}\right|+\left|E_{\mathrm{nitrate}}\right|}$$

Its ensemble average rises from approximately 0.617 in the far-window reference to 0.916 in the inner-sphere ensemble. The corresponding inner-minus-far contrast is

68 $$\Delta C_{1}=0.2993$$

with a 95% cluster-bootstrap interval of [0.2206, 0.3801] and Cohen’s *d* = 2.05. Therefore, the nitrate approach does not simply “turn up” a pre-existing static field. Instead, it generates large but mutually compensating electrostatic components. This distinction is crucial for interpreting hypersensitive Eu^3+^ emission, because a model based solely on the scalar quantity |**E**|^2^ discards the cancellation and angular information that actually changes with speciation.

### Higher Electrostatic Ranks Reveal Hidden Local Reorganization

The failure of the scalar electric-field magnitude to distinguish the conditional ensembles motivates extending the electrostatic description beyond first rank. We therefore evaluated the symmetric traceless rank-2 field-gradient tensor, *T* ^(2)^, and the symmetric traceless rank-3 coefficient, *A* ^(3)^, obtained from the local expansion of the electrostatic potential around Eu^3+^. The total rank-2 norms are

69 $$\begin{array}{l} \langle ∥T^{\left(2\right)}∥\rangle_{\mathrm{inner}}=0.4567\\ \langle ∥T^{\left(2\right)}∥\rangle_{\mathrm{interconversion}}=0.5270\\ \langle ∥T^{\left(2\right)}∥\rangle_{\mathrm{outer}}=0.4678\\ \langle ∥T^{\left(2\right)}∥\rangle_{\mathrm{far}}=0.4537 \end{array}$$

As for the electric field, the inner-minus-far difference is negligible

70 $$\Delta ∥T^{\left(2\right)}{∥}_{\mathrm{inner}-\mathrm{far}}=0.00294$$

with a 95% cluster-bootstrap interval of [−0.02209, 0.03092] and *d* = 0.019. The interconversion ensemble, however, exhibits a moderate enhancement:

71 $$\Delta ∥T^{\left(2\right)}{∥}_{\mathrm{interconversion}-\mathrm{far}}=0.07326$$

with 95% confidence interval [0.04646, 0.10304] and *d* = 0.454. This identifies the interconversion region as a locally more anisotropic environment at second rank, even though the stable inner and far ensembles possess nearly identical total rank-2 norms. The same qualitative conclusion emerges even more clearly at rank 3. The total odd-rank tensor norms are

72 $$\begin{array}{l} \langle ∥A^{\left(3\right)}∥\rangle_{\mathrm{inner}}=0.26634\\ \langle ∥A^{\left(3\right)}∥\rangle_{\mathrm{interconversion}}=0.25472\\ \langle ∥A^{\left(3\right)}∥\rangle_{\mathrm{outer}}=0.26927\\ \langle ∥A^{\left(3\right)}∥\rangle_{\mathrm{far}}=0.26368 \end{array}$$

For the inner-minus-far comparison,

73 $$\Delta ∥A^{\left(3\right)}∥=0.00266$$

with a 95% cluster-bootstrap interval of [−0.01255, 0.01725] and *d* = 0.043. The interconversion-minus-far effect also remains small (*d* = −0.129). Taken together, the stable inner and far ensembles exhibit nearly identical total scalar electrostatic magnitudes at all three ranks, whereas the interconversion ensemble displays a resolved rank-2 enhancement without a corresponding amplification at ranks 1 or 3, as summarized in Figure 2a. The comparatively modest state dependence of the *total* tensor norms conceals a remarkably strong rank-dependent amplification of the nitrate contribution. Using the far conditional ensemble as reference, we obtain
74

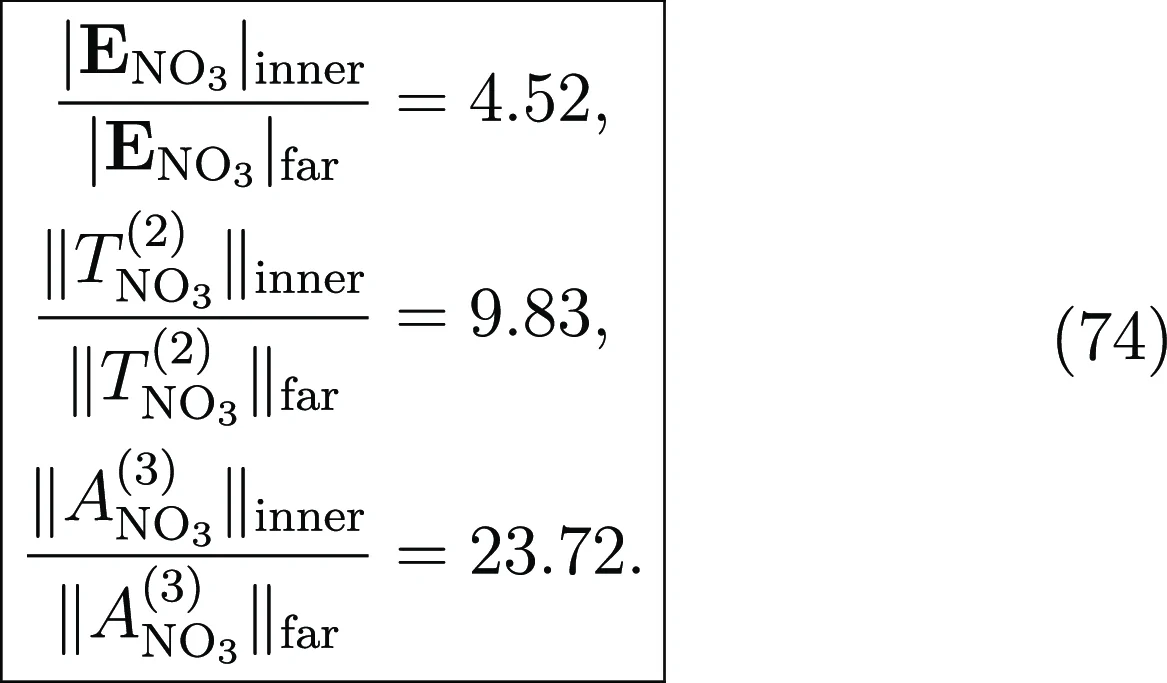




**Figure 2:**
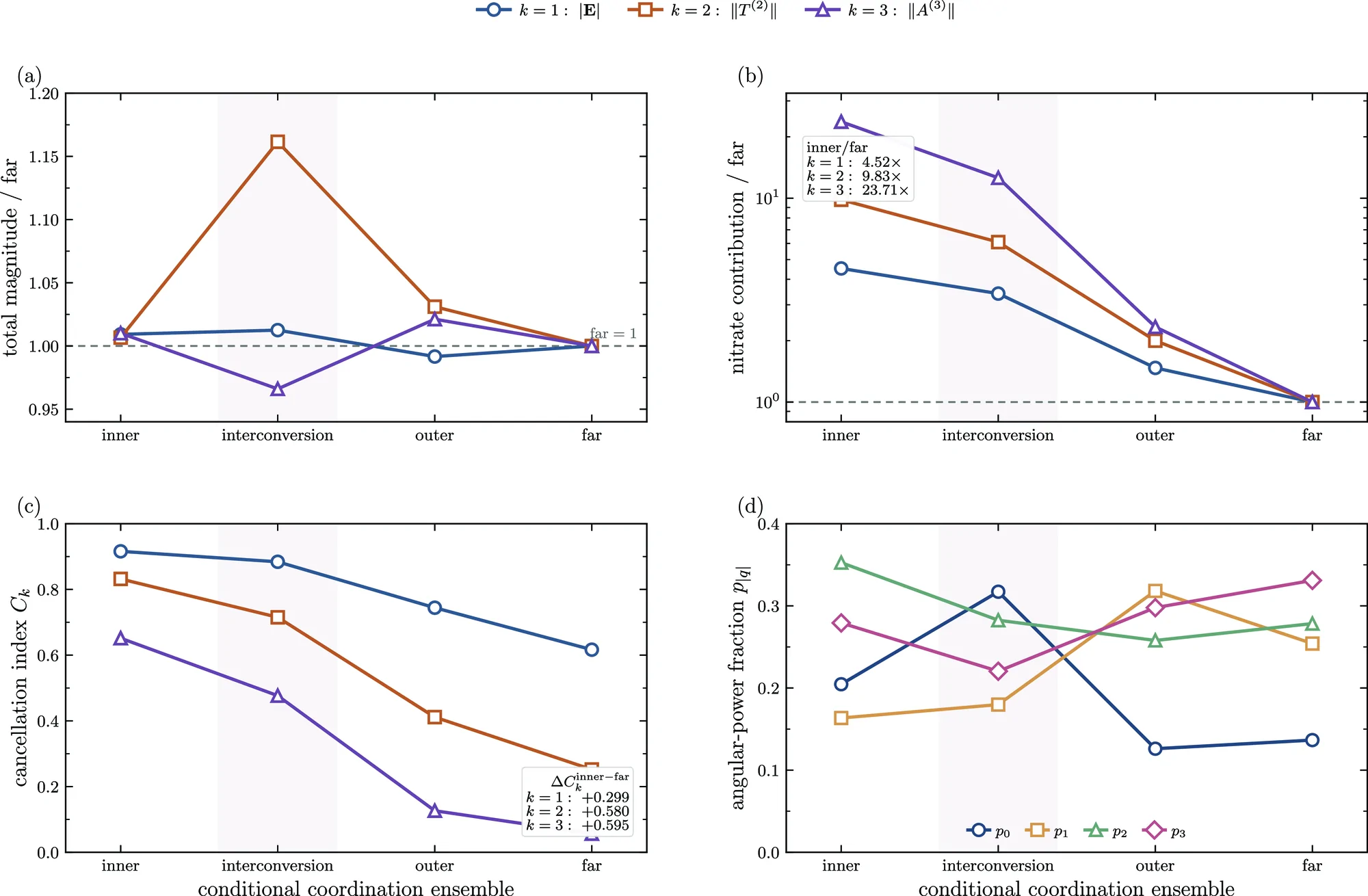
Electrostatic reorganization across the conditional Eu^3+^–nitrate coordination ensembles. (a) Total rank-resolved electrostatic magnitudes, normalized by the corresponding values in the far conditional ensemble. The rank-1 electric-field magnitude |**E**|, rank-2 STF field-gradient norm ∥*T* ^(2)^∥, and rank-3 odd-parity STF norm ∥*A* ^(3)^∥ remain comparatively weakly state-dependent overall, although the interconversion ensemble exhibits a resolved rank-2 enhancement. These comparatively modest changes in the total scalar magnitudes contrast with the pronounced state dependence of the individual solvent and nitrate contributions. (b) Nitrate contribution normalized by its corresponding far-ensemble value. Nitrate amplification increases strongly with electrostatic rank, reaching inner/far ratios of approximately 4.52, 9.83, and 23.71 for *k* = 1, 2, and 3, respectively; the same rank hierarchy persists in the interconversion ensemble. (c) Rank-dependent cancellation index *C*_*k*_ between solvent and nitrate contributions. Cancellation increases markedly upon nitrate approach, with inner–far differences Δ*C*_1_ = 0.299, Δ*C*_2_ = 0.580, and Δ*C*_3_ = 0.595, showing that the growing nitrate contribution at higher rank is largely compensated by the surrounding solvent response. (d) Molecular-frame decomposition of the total rank-3 tensor into angular-power fractions *p*_0_–*p*_3_, shown as separate line-and-marker series to facilitate comparison across the conditional coordination ensembles. The fractions satisfy *p*_0_ + *p*_1_ + *p*_2_ + *p*_3_ = 1 in every ensemble. The interconversion ensemble exhibits an enhanced *q* = 0 contribution, with *p*_0_ = 0.317, relative to the far conditional ensemble (*p*_0_ = 0.137), revealing angular reorganization that is not apparent from the total scalar norm alone. The lightly shaded column highlights the interconversion conditional ensemble.

The corresponding 95% source-cluster confidence intervals are [3.42, 6.39], [7.31, 13.98], and [15.96, 40.26], respectively. The interconversion ensemble exhibits the same hierarchy, 3.40 → 6.09 → 12.59 for ranks *k* = 1, 2, and 3. The corresponding amplification factors and their source-cluster bootstrap confidence intervals are compared directly in Figure 5. Importantly, the comparison is made within each electrostatic rank: because the rank-1, rank-2, and rank-3 quantities have different physical dimensions, the figure compares state-to-reference ratios rather than absolute magnitudes across ranks.

This systematic progression demonstrates that short-range nitrate coordination becomes increasingly visible at higher electrostatic rank. Higher spatial derivatives of the electrostatic potential naturally emphasize the nearest and most anisotropically distributed charges, and therefore provide a more local probe of coordination than the net electric field. Notably, the enhanced nitrate contribution does not propagate into a correspondingly larger total tensor norm because cancellation becomes progressively stronger. Defining rank-dependent cancellation indices analogously to eq 67, we find

75 $$\begin{array}{l} \Delta C_{1}^{\mathrm{inner}-\mathrm{far}}=0.2993\\ \Delta C_{2}^{\mathrm{inner}-\mathrm{far}}=0.5799\\ \Delta C_{3}^{\mathrm{inner}-\mathrm{far}}=0.5950 \end{array}$$

with standardized effect sizes

76 $$d\left(C_{1}\right)=2.05,,d\left(C_{2}\right)=4.62,,d\left(C_{3}\right)=8.69$$

The full state dependence of this cancellation hierarchy is shown in Figure 2c. The increasingly large cancellation upon nitrate approach explains why substantial changes in the individual water and nitrate contributions remain largely hidden in the corresponding total scalar norms. This contrast is central to the mechanistic interpretation
77

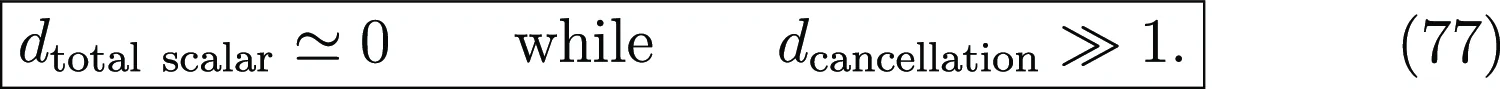




The spectroscopically relevant information introduced by nitrate association is therefore largely hidden from scalar norms and resides instead in the tensorial decomposition of the local environment.

The tensorial reorganization can be connected directly to the local molecular geometry of the conditional ensemble. In the far- and outer-sphere states, the selected nitrate remains outside the first coordination shell and the Eu^3+^ ion retains nine coordinated water molecules (*N*_w_ = 9, *d*_NO_3__ = 0). In contrast, the inner-sphere ensemble contains eight first-shell waters and one directly coordinated nitrate oxygen (*N*_w_ = 8, *d*_NO_3__ = 1). Inner-sphere nitrate association therefore involves first-shell water displacement and reorganization rather than the simple addition of an anion to an otherwise unchanged hydration shell.

This local structural change provides a microscopic interpretation of the rank dependence. Higher spatial derivatives of the electrostatic potential place progressively greater weight on nearby and anisotropically distributed charges, so changes in the position and orientation of the selected nitrate and of the surrounding first-shell waters can produce pronounced rank-2 and rank-3 responses even when the resultant first-rank field magnitude changes very little. The increasing water–nitrate cancellation with electrostatic rank further indicates that the solvent environment responds collectively to nitrate approach. The present analysis does not, however, define a unique water-orientational order parameter, and the tensor response should therefore be interpreted as a collective local-geometry signature rather than assigned to one specific water-rotation mode. A schematic molecular representation of this structural progression is shown in Figure 3.

**Figure 3:**
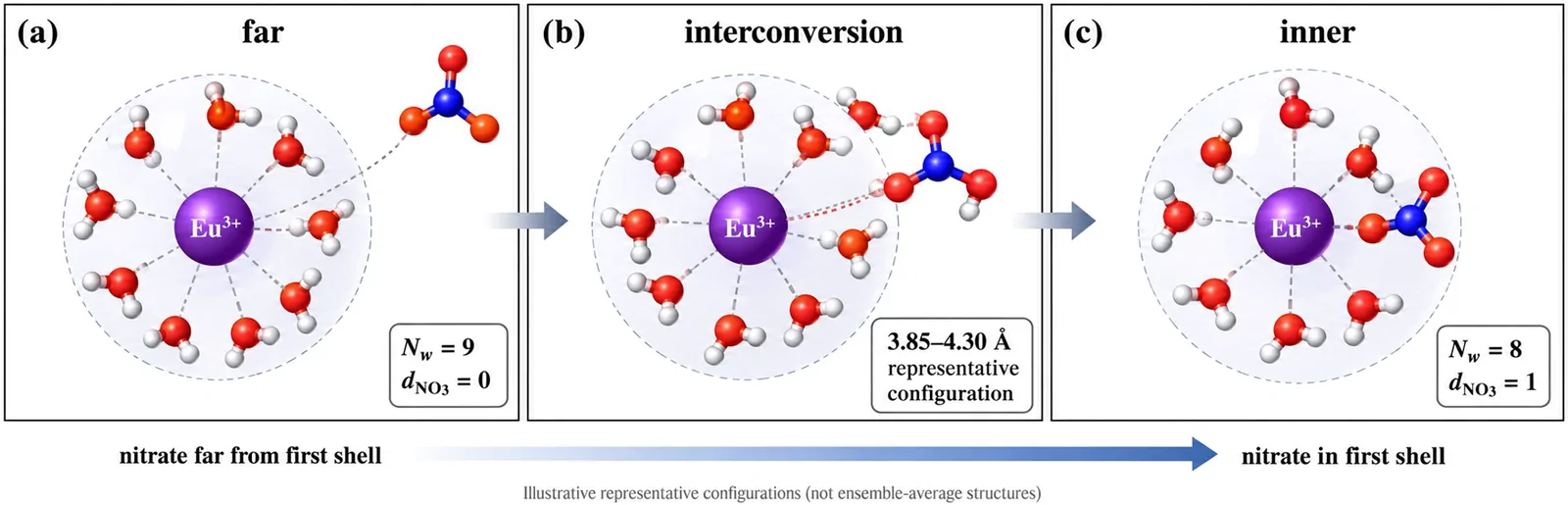
Schematic molecular representation of the local structural reorganization accompanying nitrate approach to hydrated Eu^3+^. (a) Far-window configuration: the selected nitrate lies outside the first coordination shell, while Eu^3+^ retains nine coordinated water molecules (*N*_w_ = 9, *d*_NO_3__ = 0). (b) Interconversion-region representation (3.85 ≤ *r*_Eu–N_ ≤ 4.30 Å), illustrating the progressive reorganization of the local hydration environment as nitrate approaches the first shell. (c) Inner-sphere configuration: one nitrate oxygen is directly coordinated to Eu^3+^ and the first hydration shell contains eight water molecules (*N*_w_ = 8, *d*_NO_3__ = 1). The drawings are schematic illustrations of the structural criteria defining the conditional ensembles and are not simulation snapshots or ensemble-average molecular structures.

### Odd-Rank Angular Composition Distinguishes Coordination Environments

A scalar tensor norm is invariant under rotation and is therefore useful for measuring the overall strength of an electrostatic perturbation. However, precisely because of this invariance, it discards information about how the perturbation is distributed among angular channels. This distinction becomes particularly important for the odd-rank *A* ^(3)^ tensor. We therefore transformed *A* ^(3)^ into a molecular reference frame whose *z* axis is defined by the Eu–N direction of the selected nitrate and decomposed the tensor into real tesseral rank-3 components. The squared norm was grouped into four rotationally well-defined angular-power fractions,

78 $$p_{0}+p_{1}+p_{2}+p_{3}=1$$

where *p*_|*q*|_ denotes the fraction of the total rank-3 power associated with the corresponding |*q*| sector. The ensemble-averaged distributions are
79

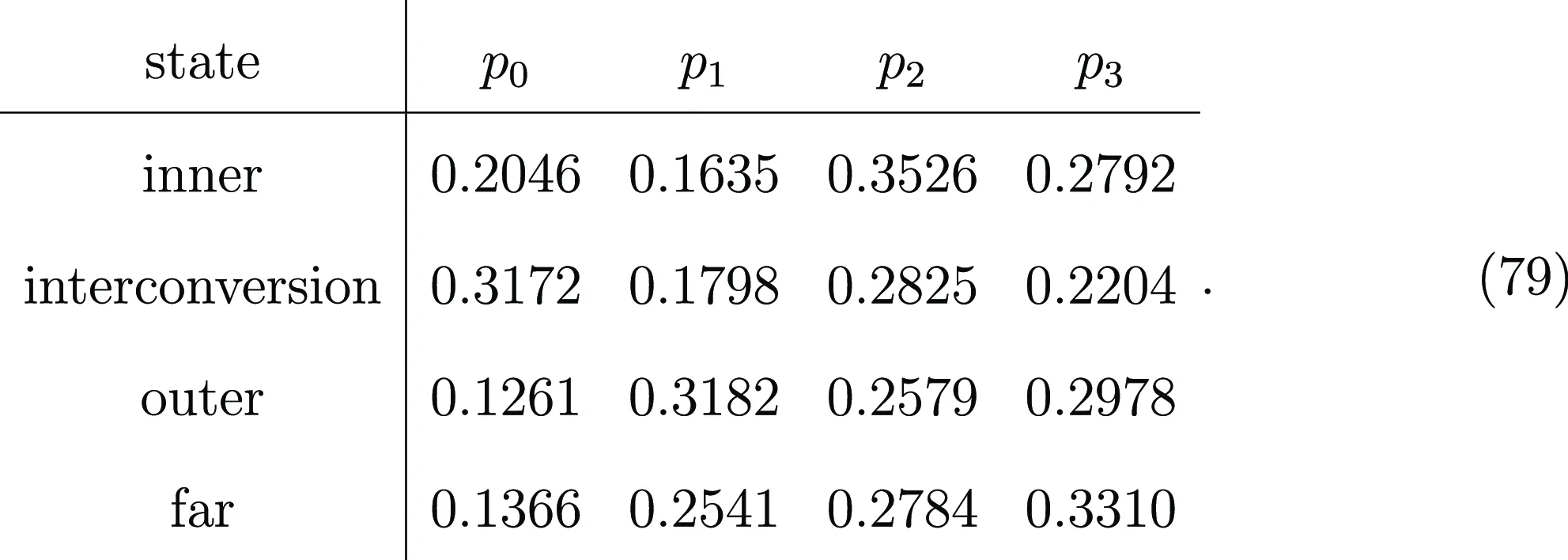




The redistribution of the grouped angular-power fractions across the four conditional environments is shown in Figure 2d. Unlike the total rank-3 norm, the angular composition changes systematically between coordination environments. For the inner-minus-far comparison,

80 $$\begin{array}{l} \Delta p_{0}=+0.0681\\ \Delta p_{1}=-0.0906\\ \Delta p_{2}=+0.0742\\ \Delta p_{3}=-0.0517 \end{array}$$

The corresponding source-cluster 95% confidence intervals are

81 $$\begin{array}{l} \Delta p_{0}:\left[+0.0195,\;+0.1175\right]\\ \Delta p_{1}:\left[-0.1404,\;-0.0418\right]\\ \Delta p_{2}:\left[+0.0274,\;+0.1167\right]\\ \Delta p_{3}:\left[-0.0941,\;-0.0067\right] \end{array}$$

Thus, all four grouped angular channels are measurably redistributed upon formation of the inner-sphere configuration even though ∥*A* ^(3)^∥ itself remains unchanged. The broad interconversion-region conditional ensemble exhibits an even more distinctive angular signature. In particular,

82 $$\Delta p_{0}^{\mathrm{interconversion}-\mathrm{far}}=+0.1807$$

with a 95% cluster-bootstrap confidence interval of

83 [+0.0864,+0.2752]

and Cohen’s *d* = 0.833. At the same time,

84 $$\Delta p_{3}^{\mathrm{interconversion}-\mathrm{far}}=-0.1105$$

with 95% confidence interval [−0.1756, −0.0429] and *d* = −0.516. The *p*_2_ channel, by contrast, is essentially unchanged (Δ*p*_2_ = 0.0042, *d* = 0.018).

The interconversion region is therefore characterized not by a larger total odd-rank field, but by a redistribution toward a more pronounced *q* = 0 contribution aligned with the Eu–N molecular axis. In other words, this broad conditional region is *angularly* more distinctive than it is *electrostatically stronger*. Because the interval 3.85–4.30 Å straddles the one-dimensional PMF maximum rather than isolating a dividing surface, this contrast is not interpreted as a transition-state property.

This result establishes a robust coordination-dependent signature in the angular organization of the odd-rank electrostatic environment. Its possible spectroscopic significance follows from symmetry: odd-rank electrostatic terms possess the parity required to participate in the coupling of electronic configurations of opposite parities. Consequently, changes in the angular structure of the odd-rank local potential identify a plausible environmental channel through which coordination may influence intensity borrowing mechanisms involving the 4f^6^ manifold and opposite-parity configurations.

This connection remains a symmetry-based mechanistic hypothesis rather than a demonstrated causal relation. In particular, the present analysis does not establish that *A* ^(3)^ or any individual *p*_*q*_ component controls the ^5^ *D*_0_ → ^7^ *F*_2_ intensity, nor does it provide a quantitative mapping to a Judd–Ofelt intensity parameter. The angular signature also survives an explicit configurational stress test of the transferred Eu–nitrate *C*_4_ terms. At coherent scale factors λ = 0.8, 1.0, 1.2, the equal-source interconversion-minus-far contrasts are

85 $$\Delta p_{0}=0.2540,\;0.2744,\;0.1880$$

with equal-source temporal-block 95% intervals [0.1099, 0.4003], [0.1661, 0.3683], and [0.0312, 0.3514], respectively. The positive sign is retained in every leave-one-source-out analysis at all three Hamiltonians. The λ = 1.0 branch therefore reproduces the historical qualitative interconversion-region enrichment, and the two ± 20% extremes retain it. No monotonic dependence of Δ*p*_0_ on *C*_4_ is inferred; the result is instead a robustness statement about the persistence of the angular reorganization under substantial configurational perturbation. Because the interconversion samples contain fewer than the conservative target of 120 configurations, this test is reported as a targeted diagnostic rather than as an independent production estimate of the state distribution.

The principal inferential comparisons are summarized in Figure 4. The source-cluster bootstrap contrasts emphasize the central distinction of the present analysis: changes in the total scalar electrostatic invariants are small or negligible, whereas solvent–nitrate cancellation and selected rank-3 angular components exhibit substantially larger and well-resolved changes.

**Figure 4:**
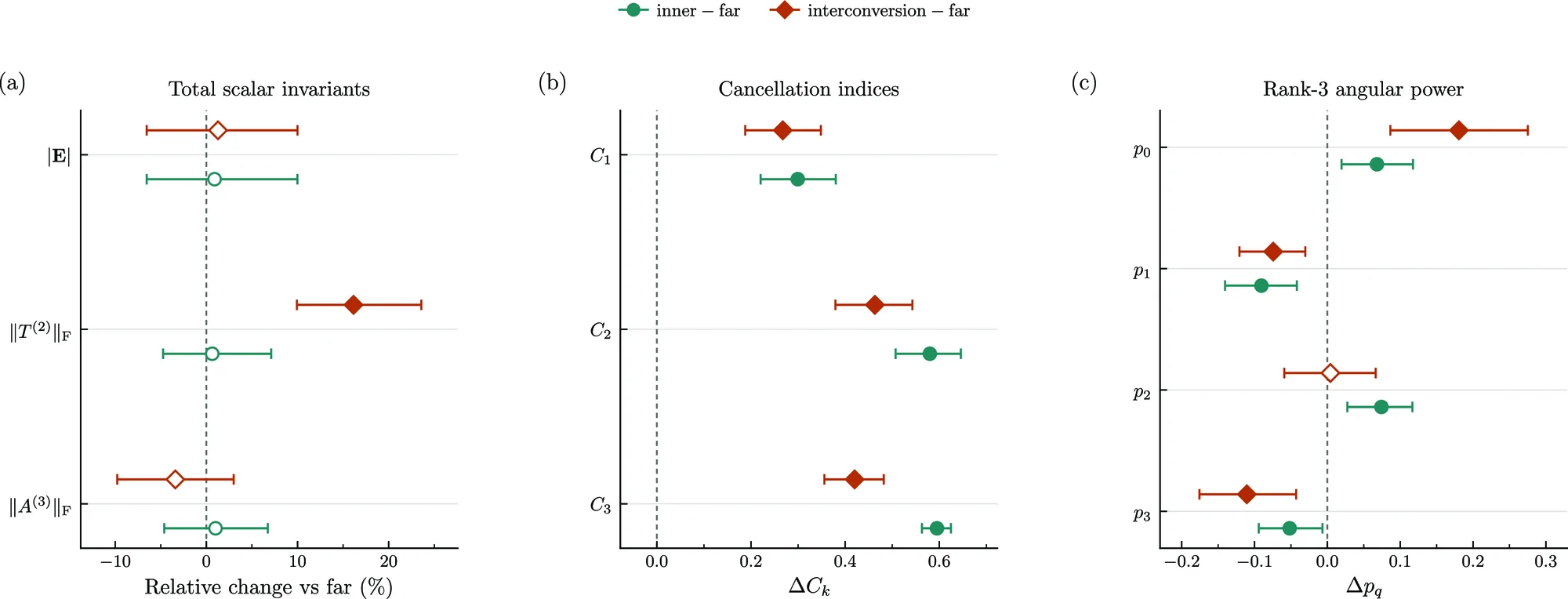
Statistical contrasts between the inner and interconversion conditional ensembles and the far-window reference. Error bars denote 95% confidence intervals obtained from source-level cluster bootstrap. Filled symbols denote contrasts whose 95% confidence intervals exclude zero, whereas open symbols denote intervals that include zero. (a) Relative changes in the total scalar electrostatic invariants. (b) Differences in rank-dependent cancellation indices. (c) Differences in the rank-3 angular-power fractions. The near invariance of the total scalar magnitudes for stable inner–far coordination contrasts stands in sharp contrast to the large changes in solvent–nitrate cancellation and to the selective redistribution of the odd-rank angular channels. The far ensemble is a conditional far-window reference and is not interpreted as an infinite-dilution bulk state.

### Quantum Electronic Structure: Scope and Limitations of the Explicit Intensity Mechanism

The classical electrostatic analysis establishes that coordination is encoded primarily in cancellation and angular structure rather than in the magnitude of the static field. We next examined how far this environmental information can be connected explicitly to the low-energy electronic structure of Eu^3+^. State-averaged CASSCF calculations using a CAS­(6,7) active space yield seven predominantly Eu-4f orbitals with six active electrons. Population analysis confirms that these orbitals retain overwhelmingly Eu-4f character, establishing the active space as a physically clean representation of the 4f^6^ configuration. Spin–orbit coupling among the seven septet states reproduces the expected ^7^ *F*_*J*_ manifold, with the *J* = 0,..., 6 groups occurring with the appropriate (2*J* + 1) multiplicities. Separate state-averaged quintet calculations identify the lowest ^5^ *D* manifold. Inclusion of strongly contracted NEVPT2 dynamic correlation lowers the spin-free ^5^ *D*–^7^ *F* separation by approximately 2.0 × 10^3^ cm^–1^ relative to the CASSCF result .

**Figure 5:**
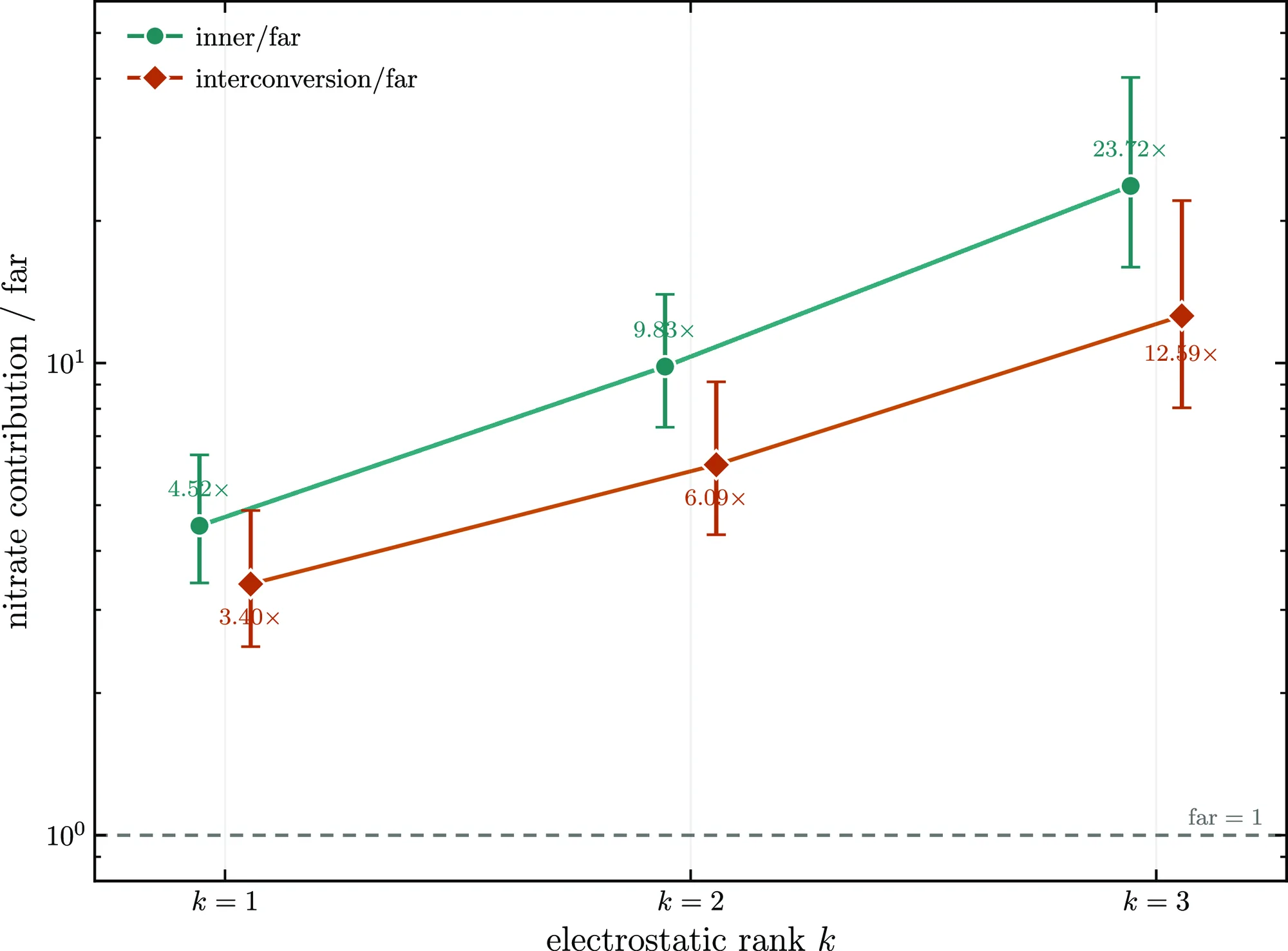
Rank-dependent amplification of the nitrate electrostatic contribution relative to the far conditional ensemble. Symbols denote ratios of ensemble-averaged nitrate contributions and error bars represent 95% source-cluster bootstrap confidence intervals. Results are shown for the inner-sphere and interconversion conditional ensembles across electrostatic ranks *k* = 1, 2, and 3, corresponding respectively to the electric-field magnitude |**E**_NO_3__|, the rank-2 STF field-gradient norm ∥*T*_NO_3__^(2)^∥, and the rank-3 odd-parity STF norm ∥*A*_NO_3__^(3)^∥. Relative to the far reference, the nitrate contribution in the inner ensemble increases from approximately 4.52× at *k* = 1 to 9.83× at *k* = 2 and 23.72× at *k* = 3. The interconversion ensemble exhibits the same rank hierarchy, with corresponding amplification factors of approximately 3.40×, 6.09×, and 12.59×. The dashed horizontal line marks the reference value of unity. Because quantities of different electrostatic rank have different physical dimensions, comparisons are made only through state-to-reference ratios within each rank. The far ensemble is used as a conditional reference and is not interpreted as an infinite-dilution bulk state.

Using the SC-NEVPT2 energies as the diagonal spin-free Hamiltonian in the subsequent RASSI spin–orbit treatment gives the lowest ^5^ *D*_0_-dominated state at approximately

86 $$2.09\times 10^{4}\;{\mathrm{cm}}^{-1}≃2.59\;\mathrm{eV}$$

Although dynamic correlation substantially improves the ^5^ *D*–^7^ *F* separation, the calculated energy remains too high for a quantitatively accurate description of the experimental Eu^3+5^ *D*_0_ emission region. We therefore use these calculations to establish the electronic-state topology rather than to perform an empirical spectral fit. The spin–orbit eigenvector associated with the nominal ^5^ *D*_0_ state contains approximately 94.7% quintet and 5.3% septet character. Thus, spin–orbit coupling already provides measurable spin borrowing within the 4f^6^ manifold. This result is relevant to the magnetic-dipole and spin-forbidden aspects of Eu^3+^ spectroscopy, but it must not be confused with the opposite-parity mixing required for a full description of forced electric-dipole intensity. Indeed, the CAS­(6,7) model contains only the seven Eu-4f orbitals and therefore cannot represent configurations such as

87 $$4f^{6}↔4f^{5}5d^{1}$$

which provide one important opposite-parity channel for electric-dipole intensity borrowing. An exploratory extension of the active space was consequently performed: five Eu-d-like orbitals were identified from improved virtual orbitals and introduced together with the seven 4f orbitals. A constrained GAS calculation enforcing the 4f^5^5d^1^ sector confirmed that this electronic subspace is numerically accessible; however, under low-state averaging, the d electron density collapsed almost entirely onto only two crystal-field components. The remaining nominal 5d orbitals became nearly unoccupied and rotated strongly into the secondary ligand space, preventing stable convergence of a balanced five-orbital d shell.

The rotation of several nominal Eu-5d orbitals toward ligand space also cautions against interpreting the relevant opposite-parity manifold as a purely metal-centered 4f^5^5d^1^ sector. In the embedded solution environment considered here, opposite-parity configurations may acquire mixed metal–ligand character and may involve ligand polarization, metal–ligand overlap, or ligand-involved charge-transfer-like contributions. The present exploratory GAS calculation does not provide a balanced, state-consistent description of these additional channels, and we therefore do not attempt to assign quantitative weights to them. A controlled treatment would require an active space capable of representing both the metal-centered and the relevant ligand-based orbitals on an equal footing.

Rather than introduce increasingly elaborate orbital constraints or state-selection procedures, we therefore regard the explicit 4f^6^–4f^5^5d^1^ intensity problem as beyond the quantitatively controlled scope of the present study. No transition intensity derived from the unconverged extended-active-space calculation is used in the analysis. This limitation is important for interpreting the mechanistic result. The present calculations demonstrate that

1. The low-energy 4f^6^ manifold is described consistently by the CASSCF/SC-NEVPT2/SOC treatment;
2. Nitrate speciation does not significantly change the total first-rank static-field magnitude;
3. Coordination strongly amplifies individual ligand contributions at progressively higher electrostatic rank;
4. This amplification is masked in scalar observables by increasing solvent–anion cancellation; and
5. The odd-rank angular composition nevertheless retains a clear and statistically robust signature of coordination.

What remains unresolved is the final quantitative electronic mapping

88 $$\left\{p_{q},\;A_{q}^{\left(3\right)},\;\mathrm{coordination}\right\}\to \left\{\mathrm{opposite}‐\mathrm{parity}\;\mathrm{admixture},\Omega_{2},A_{\mathrm{ED}}\right\}$$

which will require an electronic-structure treatment capable of describing both metal-centered and ligand-involved opposite-parity configurations in a balanced and state-consistent active space.

### Implications for the Static-Dipole Interpretation of Eu^3+^ Hypersensitivity

The combined molecular-dynamics and electronic-structure results therefore lead to a substantially different interpretation of the static-dipole mechanism from that obtained from a simple scalar-field model. If the hypersensitive response were governed primarily by the magnitude of the local static field, a progression from the far to the inner-sphere state should produce a systematic increase in |**E**| and, consequently, in any model assuming

89 $$\Omega_{2}\propto |E_{\mathrm{stat}}{|}^{2}$$

No such increase is observed. The inner and far conditional ensembles differ in |**E**| by less than 1%, and the corresponding effect size is essentially zero. The same near invariance holds for the inner–far contrasts of the total rank-2 and rank-3 electrostatic tensor norms. At the same time, it would be incorrect to conclude that nitrate coordination produces only a weak electrostatic perturbation. The nitrate contribution itself grows by factors of approximately 4.5, 9.8, and 23.7 from rank 1 to rank 3, respectively. What suppresses the scalar response is an increasingly efficient cancellation against the surrounding hydration environment.

The appropriate microscopic distinction is therefore not

90 weakfield→strongfield

but rather
91

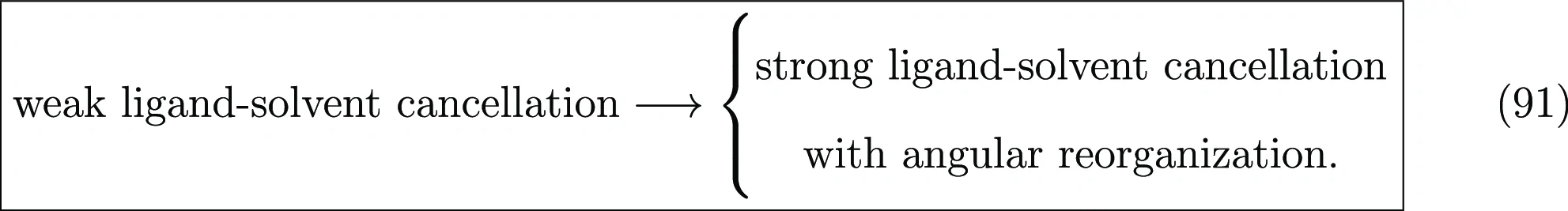




This distinction reconciles two observations that may otherwise appear contradictory: the local ligand environment changes dramatically as nitrate approaches Eu^3+^, whereas the net static electric field at the metal changes very little. For Eu^3+^ hypersensitive transitions, this result suggests that a scalar descriptor such as |**E**|^2^ is intrinsically incomplete. A physically richer description must retain at least part of the angular and multipolar structures of the local electrostatic potential. In the present system, the odd-rank *k* = 3 decomposition is particularly informative because its total norm is nearly invariant, while its angular composition changes strongly and selectively with speciation.

The pronounced enhancement of the *q* = 0 channel within the broad inner/outer-sphere interconversion-region conditional ensemble is especially noteworthy. Because odd-rank electrostatic terms can participate in opposite-parity mixing, this angular redistribution offers a plausible environmental route by which changes in nitrate coordination could contribute to opposite-parity mixing without any substantial increase in the net electric-field magnitude. The present calculations do not establish a direct quantitative mapping from *p*_0_ to Ω_2_ or to an emission intensity.

Accordingly, the present results do not support using Ω_2_ ∝ |**E**_stat_|^2^ as a universal molecular descriptor of coordination in hydrated Eu^3+^–nitrate complexes. Instead, these results indicate that environmental information potentially relevant to a microscopic description of hypersensitivity is retained in a hierarchy of tensorial electrostatic descriptors, solvent–anion cancellation, and odd-rank angular structure.

This interpretation is compatible with quantum extensions of Judd–Ofelt-type models in which static electrostatic perturbations enter through symmetry-resolved couplings rather than through a single scalar field magnitude.^13^ The present results provide a molecular-level basis for such an extension: coordination strongly modifies the symmetry and multipolar content of the local field while leaving its net first-rank magnitude almost unchanged (Table 3).

**Table 3:** Key Statistical Contrasts between the Inner or Interconversion Conditional Ensembles and the Far-Window Reference^a^

Observable	Contrast	Δ	95% CI	Cohen *d*	Interpretation
∥**E**∥	inner–far	+0.00216	[−0.01593, +0.02363]	+0.020	negligible
∥*T* ^(2)^∥	inner–far	+0.00294	[−0.02209, +0.03092]	+0.019	negligible
∥*A* ^(3)^∥	inner–far	+0.00266	[−0.01255, +0.01725]	+0.043	negligible
∥*T* ^(2)^∥	interconversion–far	+0.07326	[+0.04646, +0.10304]	+0.454	small–moderate
*C*_1_	inner–far	+0.29927	[+0.22055, +0.38007]	+2.052	very large
*C*_2_	inner–far	+0.57991	[+0.50714, +0.64592]	+4.622	very large
*C*_3_	inner–far	+0.59501	[+0.56305, +0.62469]	+8.689	very large
*p*_0_	interconversion–far	+0.18069	[+0.08643, +0.27522]	+0.833	large
*p*_3_	interconversion–far	–0.11052	[−0.17564, −0.04286]	–0.516	moderate

a Confidence intervals are source-cluster bootstrap 95% intervals. The far state is a conditional reference and not an infinite-dilution bulk state. The *p*_0_ and *p*_3_ intervals use the final source-cluster robustness analysis of the broad 3.85–4.30 Å interconversion ensemble.

## Conclusions

In this work, the electrostatic consequences of nitrate association with hydrated Eu^3+^ were examined beyond a scalar static field description by combining molecular dynamics, free energy sampling, periodic multipolar analysis, and multireference electronic structure calculations. The resulting picture differs substantially from that in which nitrate coordination is expected simply to increase the magnitude of the electric field at the lanthanide center.

A 12–6–4 description of Eu^3+^ in TIP4P-Ew water produces a strongly hydrated ion with an approximately nine-coordinate first solvation shell. Along the Eu–N association coordinate, the locally converged potential of mean force indicates that outer-sphere nitrate coordination is favored over the inner-sphere state by approximately 2.6 kcal mol^–1^. The one-dimensional free-energy rise from the outer-sphere basin to the barrier-like maximum is approximately 5.2 kcal mol^–1^, whereas the corresponding rise from the inner-sphere basin is approximately 2.7 kcal mol^–1^. These results emphasize that nitrate association occurs against a substantial hydration background and that proximity of the anion cannot be interpreted independently of solvent reorganization.

The central mechanistic result is that this structural reorganization is almost invisible in the magnitude of the resultant periodic electric field. The inner- and far-window conditional ensembles differ in |**E**| by only 0.0022 V Å^–1^, corresponding to a negligible standardized effect (*d* = 0.02). The scalar norms of the symmetric traceless rank-2 field-gradient tensor and of the odd-rank rank-3 electrostatic tensor are similarly insensitive to stable inner-sphere association. Thus, neither |**E**| nor a simple replacement of it by progressively higher scalar tensor norms provides a sufficiently discriminating descriptor of Eu^3+^–nitrate speciation.

The apparent invariance of these total quantities does not imply an electrostatically invariant coordination environment. On the contrary, the nitrate contribution increases dramatically as increasingly local spatial derivatives of the potential are considered. Relative to the far-window ensemble, inner-sphere association amplifies the nitrate contribution by factors of approximately 4.5, 9.8, and 23.7 for ranks *k* = 1, 2, and 3, respectively. This increasing local perturbation is compensated by an equally striking reorganization of the hydration environment. The corresponding water–nitrate cancellation indices increase strongly upon inner-sphere association, with inner–far contrasts of approximately 0.30, 0.58, and 0.60 for ranks 1, 2, and 3. The nearly invariant total tensors therefore arise from *cancellation through correlated molecular reorganization*, rather than from the absence of a strong nitrate perturbation.

The rank-3 analysis further demonstrates that important coordination information survives even when its scalar norm is buffered. Resolving the odd-rank tensor in the instantaneous Eu–N molecular frame reveals a significant redistribution of angular power among its tesseral components. Most notably, the broad interconversion-region conditional ensemble exhibits a pronounced enhancement of the axial *q* = 0 contribution relative to the far-window reference,

92 $$\Delta p_{0}≃0.181,,d≃0.83$$

with a final source-cluster 95% interval of [0.086, 0.275]. The positive interconversion-minus-far signature persists in matched explicit-MD stress tests in which the transferred Eu–nitrate *C*_4_ coefficients are coherently scaled by ±20%; source-balanced temporal-block intervals remain positive at all three Hamiltonians. Together with the separate PMF stress test, this shows that neither the local basin ordering nor the existence of the *q* = 0 angular enrichment depends on fine-tuning of the transferred nitrate induction terms within the tested range. This is a robustness statement, not a claim of monotonic *p*_0_ dependence on *C*_4_.

The redistribution is accompanied by compensating changes in the nonaxial components. The coordination coordinate is therefore encoded not simply in the *strength* of the odd-parity electrostatic perturbation but also in its *angular organization*. This distinction is particularly relevant for microscopic interpretations of forced electric dipole transitions, for which odd-parity components of the environmental potential provide the symmetry required for coupling to opposite-parity electronic configurations.

The electronic-structure calculations independently establish a consistent Eu^3+^ 4f^6^ backbone. State-averaged CAS­(6,7) wave functions, SC-NEVPT2 dynamic-correlation corrections, and spin–orbit state interaction recover the expected low-lying ^7^ *F*_*J*_ and ^5^ *D*_*J*_ manifolds and demonstrate non-negligible spin–orbit mixing of the nominal ^5^ *D*_0_ state. The calculated ^5^ *D*_0_ excitation remains higher than the typical aqueous Eu^3+^ spectroscopic region, however, indicating that quantitative excitation energies require electronic degrees of freedom beyond the compact 4f^6^ active space. Moreover, a balanced explicit 4f^5^5d^1^ opposite-parity active space could not be stabilized for the present embedded solution structure. The current calculations therefore support the symmetry framework of the electrostatic analysis but do not constitute a direct calculation of the forced electric-dipole intensity or of Ω_2_.

Taken together, these results argue against interpreting the hypersensitive response of hydrated Eu^3+^ through a relation of the form

93 $$\Omega_{2}\propto |E_{\mathrm{stat}}{|}^{2}$$

as a general molecular descriptor. The net field magnitude can remain nearly unchanged while the microscopic ligand perturbation, solvent compensation, and odd-rank angular structure change substantially. Consequently, the relevant molecular information resides in the *decomposition and tensorial organization* of the local electrostatic potential rather than in the magnitude of its resultant lowest-order field alone.

More broadly, the present results suggest a hierarchy of descriptors for connecting liquid-state coordination dynamics with lanthanide photophysics: scalar local fields provide the coarsest description; component-resolved higher-rank tensors expose hidden ligand–solvent compensation; and molecular-frame odd-rank angular components retain symmetry information that is directly lost upon scalar contraction. Establishing a quantitative connection between these descriptors and Eu^3+^ transition intensities will ultimately require an electronic structure treatment capable of describing the relevant opposite-parity manifolds and ligand-to-metal configurations on an equal footing. The framework developed here provides the molecular and electrostatic basis for such calculations while avoiding the assumption that stronger hypersensitive luminescence must originate from a stronger net static electric field.

## Supplementary Material



## Notes

Numerical validation of the rank-2 and rank-3 electrostatic tensors; complete molecular-frame rank-3 tesseral decomposition; periodic-to-cluster electrostatic-embedding diagnostics; additional statistical robustness analyses; complementary multireference electronic-structure results (PDF)
