# Supplementary material for: Beyond |E|2: Tensorial Electrostatic Reorganization in Hydrated Eu3+–Nitrate Complexes and Its Implications for Hypersensitive Luminescence
Source: J Phys Chem B. 2026 Oct 13;130(42):11268–85. doi: 10.1021/acs.jpcb.6c05370 (PMC13700006; doi:10.1021/acs.jpcb.6c05370)
Supplement: Supplementary file 1 [file jp6c05370_si_001.pdf]

Supporting Information

for

Beyond $|\mathbf{E}|^2$: Tensorial Electrostatic Reorganization in Hydrated Eu^{3+}–Nitrate Complexes and Its Implications for Hypersensitive Luminescence

Murilo Sodré Marques

Programa de Pós Graduação em Ciência dos Materiais (PROMAT)
Centro das Ciências Exatas e das Tecnologias,
Universidade Federal do Oeste da Bahia (UFOB), Barreiras, BA, Brazil

murilo@ufob.edu.br

Contents of the Supporting Information. This document provides numerical validation of the rank-2 and rank-3 electrostatic tensors, the complete molecular-frame rank-3 tesseral decomposition, periodic-to-cluster electrostatic-embedding diagnostics, additional statistical robustness analyses, and complementary multireference electronic-structure results.

Contents

S1 Scope, nomenclature, and conditional ensembles **S3**

S2 Numerical validation of the rank-2 electrostatic tensor **S3**

S2.1 Finite-difference definition and convergence S3

S2.2 Symmetry, trace, and component-additivity diagnostics S5

S3 Numerical validation of the rank-3 odd-parity tensor **S7**

S3.1 Finite-difference construction and STF projection S7

S3.2 Permutation symmetry, harmonic trace, STF projection, and additivity S7

S4 Molecular-frame decomposition of the rank-3 tensor **S11**

S4.1 Local molecular frame and transverse gauge S11

S4.2 Orthonormal real tesseral rank-3 basis S11

S4.3 Grouped angular-power fractions and gauge invariance S12

S4.4 State-resolved angular redistribution S12

S5 Periodic-to-cluster electrostatic embedding validation **S15**

S5.1 Finite electrostatic embeddings and periodic reference S15

S5.2 Physical STF rank-2 correction and spatial variation S17

S5.3 Periodic preservation of the odd-parity rank-3 tensor S17

S5.4 Neutral 26-site auxiliary shell S20

S5.5 FULL+AUX numerical audit and interpretive boundary S21

S6 Additional statistical robustness analyses **S23**

S6.1 Source-aware inference and configurational genealogy S23

S6.2 Robustness of the rank-3 angular signature to source and spectator-nitrate controls . S23

S6.3 Thermodynamic stress test of the transferred Eu–nitrate C_4 terms S25

S6.4 Matched-MD configurational stress test of the angular signature S27

S7 Complementary multireference electronic-structure results **S29**

S7.1 Benchmark cluster and multireference protocol S29

S7.2 Spin-free CASSCF and state-specific SC-NEVPT2 energies S30

S7.3 State-specific versus quasi-degenerate SC-NEVPT2 . . . . . S31

S7.4 Spin–orbit manifold and character of the $^5D_0$-dominated state S31

S7.5 Exploratory opposite-parity active space and scope limitation S36

S1 Scope, nomenclature, and conditional ensembles

The Supporting Information documents numerical and methodological checks underlying the electrostatic-tensor analysis reported in the main article. No additional molecular-dynamics production sampling is introduced here. Unless stated otherwise, the numerical analyses use the same frozen configurations and structural definitions as the main text.

The historical label *transition*, retained in some analysis-file names and raw data tables for provenance, corresponds throughout this Supporting Information to the broad interconversion-region conditional ensemble ($3.85 \leq r_{\mathrm{Eu-N}} \leq 4.30$ Å). It is not interpreted as a dynamical transition state or as a transition-state ensemble. The four conditional ensembles used in the electrostatic analysis are summarized in Table S1. The far ensemble is an operational conditional reference window. In particular, the 7.50–9.00 Å interval should not be interpreted as an infinite-dilution bulk state, a rigorously converged asymptotic plateau, or a standard-state association reference.

Table S1: Geometrical definitions of the conditional ensembles used in the electrostatic analysis. N_{w} is the water coordination number and $d_{\mathrm{NO_3}}$ is the denticity of the selected nitrate.

State	$r_{\mathrm{Eu-N}}$ (Å)	N_{w}	$d_{\mathrm{NO_3}}$
inner	3.55–3.80	8	1
interconversion	3.85–4.30	unrestricted	unrestricted
outer	5.35–5.80	9	0
far	7.50–9.00	9	0

S2 Numerical validation of the rank-2 electrostatic tensor

S2.1 Finite-difference definition and convergence

The Cartesian electric-field gradient was evaluated as

$$G_{ij} = \frac{\partial E_i}{\partial x_j}, \tag{S1}$$

using central finite differences,

$$G_{ij} \simeq \frac{E_i(\mathbf{r}_{\mathrm{Eu}} + \delta \mathbf{e}_j) - E_i(\mathbf{r}_{\mathrm{Eu}} - \delta \mathbf{e}_j)}{2\delta}. \tag{S2}$$

Displacements of $\delta = 0.01$, 0.02, and 0.04 Å were evaluated, and $\delta = 0.02$ Å was adopted for the production analysis. The physical rank-2 descriptor is the symmetric-traceless (STF) part of the raw gradient,

$$\mathbf{T}^{(2)} = \frac{1}{2}\left(\mathbf{G} + \mathbf{G}^{\mathsf{T}}\right) - \frac{\mathrm{Tr}\left[(\mathbf{G} + \mathbf{G}^{\mathsf{T}})/2\right]}{3}\mathbf{I}. \tag{S3}$$

Figure S1 shows the snapshot-level relative Frobenius differences among tensors obtained with the three finite-difference displacements. The distributions are highly converged: the pooled median relative difference is 5.78×10^{-4} for $\delta = 0.01$ versus 0.02 Å and 7.69×10^{-4} for $\delta = 0.02$ versus 0.04 Å. The corresponding 95th percentiles are 1.46×10^{-3} and 1.81×10^{-3}, respectively (Table S2). These values are approximately two orders of magnitude below the historical aggregate convergence screening reference of 0.10.

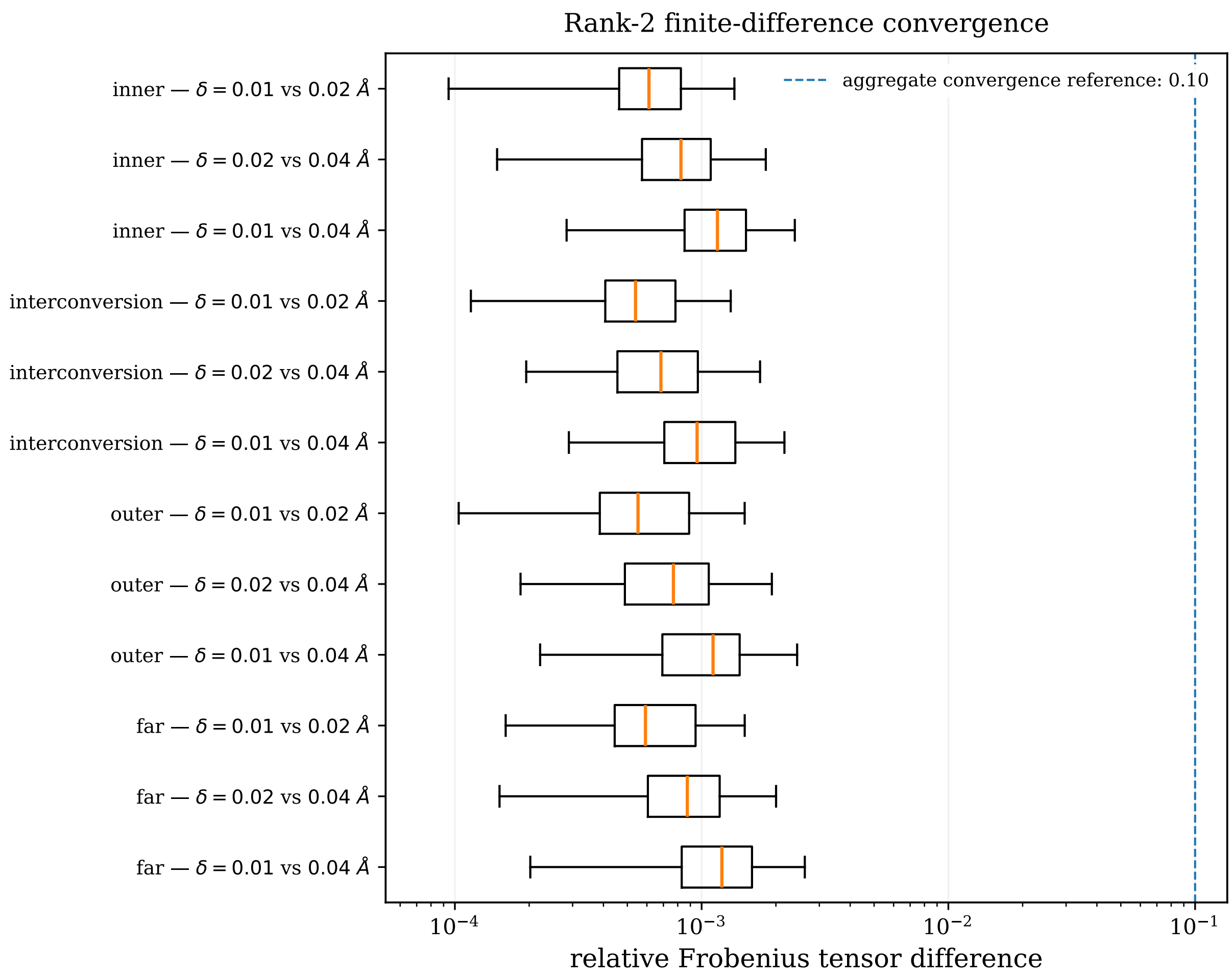

Figure S1: Finite-difference convergence of the rank-2 STF electrostatic tensor. Box plots show snapshot-level relative Frobenius differences between tensors obtained with the indicated displacement pairs for the four conditional ensembles. The historical `transition` label is displayed as *interconversion*. The dashed line marks the aggregate convergence screening reference of 0.10 used in the original numerical audit; it is not a per-snapshot rejection threshold. The logarithmic abscissa emphasizes that the observed differences are concentrated near 10^{-3} or below.

S2.2 Symmetry, trace, and component-additivity diagnostics

Three independent diagnostics were evaluated at the production displacement $\delta = 0.02$ Å. The antisymmetric residual,

$$\epsilon^{(2)}_{\mathrm{sym}} = \frac{\|\mathbf{G} - \mathbf{G}^{\mathsf{T}}\|_F}{\|\mathbf{G}\|_F}, \tag{S4}$$

tests the conservative-field symmetry expected for the electrostatic gradient. The raw Laplace-trace diagnostic was evaluated as

$$\epsilon^{(2)}_{\mathrm{tr}} = \frac{|\mathrm{Tr}\,\mathbf{G}|}{\|\mathrm{sym}(\mathbf{G})\|_F}, \tag{S5}$$

and linear component additivity was tested by comparing the directly evaluated water tensor with the difference between the total and nitrate tensors,

$$\epsilon^{(2)}_{\mathrm{add}} = \frac{\left\|\mathbf{T}^{(2)}_{\mathrm{w,direct}} - \left(\mathbf{T}^{(2)}_{\mathrm{tot}} - \mathbf{T}^{(2)}_{\mathrm{NO_3}}\right)\right\|_F}{\|\mathbf{T}^{(2)}_{\mathrm{w,direct}}\|_F}. \tag{S6}$$

As shown in Figure S2, the symmetry and additivity diagnostics are very small. Their pooled medians are 7.01×10^{-4} and 2.51×10^{-4}, respectively, with 95th percentiles of 1.64×10^{-3} and 7.42×10^{-4}. The raw trace diagnostic is larger, with median 0.0760 and 95th percentile 0.149, and therefore exceeds the conservative aggregate value of 0.05 used in the historical screening audit. This raw-trace caution does not arise from poor finite-difference convergence, loss of electrostatic symmetry, or failure of water–nitrate additivity, all of which remain well controlled. Moreover, the rank-2 quantity interpreted in the main text is explicitly the STF tensor of Eq. S3; the isotropic trace component of the raw Cartesian gradient is therefore removed by construction. The rank-2 results should accordingly be interpreted as properties of the anisotropic STF component, while the finite raw-gradient trace residual is retained here as a transparent numerical limitation of the underlying PPPM finite-difference gradient.

Table S2: Pooled numerical-validation statistics for the rank-2 analysis. The convergence quantities contain 480 snapshot comparisons; production diagnostics contain 480 production snapshots.

Diagnostic	Median	95th percentile	Aggregate reference
$\delta = 0.01$ vs 0.02 Å	5.78×10^{-4}	1.46×10^{-3}	0.10
$\delta = 0.02$ vs 0.04 Å	7.69×10^{-4}	1.81×10^{-3}	0.10
$\delta = 0.01$ vs 0.04 Å	1.10×10^{-3}	2.63×10^{-3}	—
Symmetry residual	7.01×10^{-4}	1.64×10^{-3}	0.05
Raw trace residual	7.60×10^{-2}	1.49×10^{-1}	0.05
Water additivity error	2.51×10^{-4}	7.42×10^{-4}	0.02

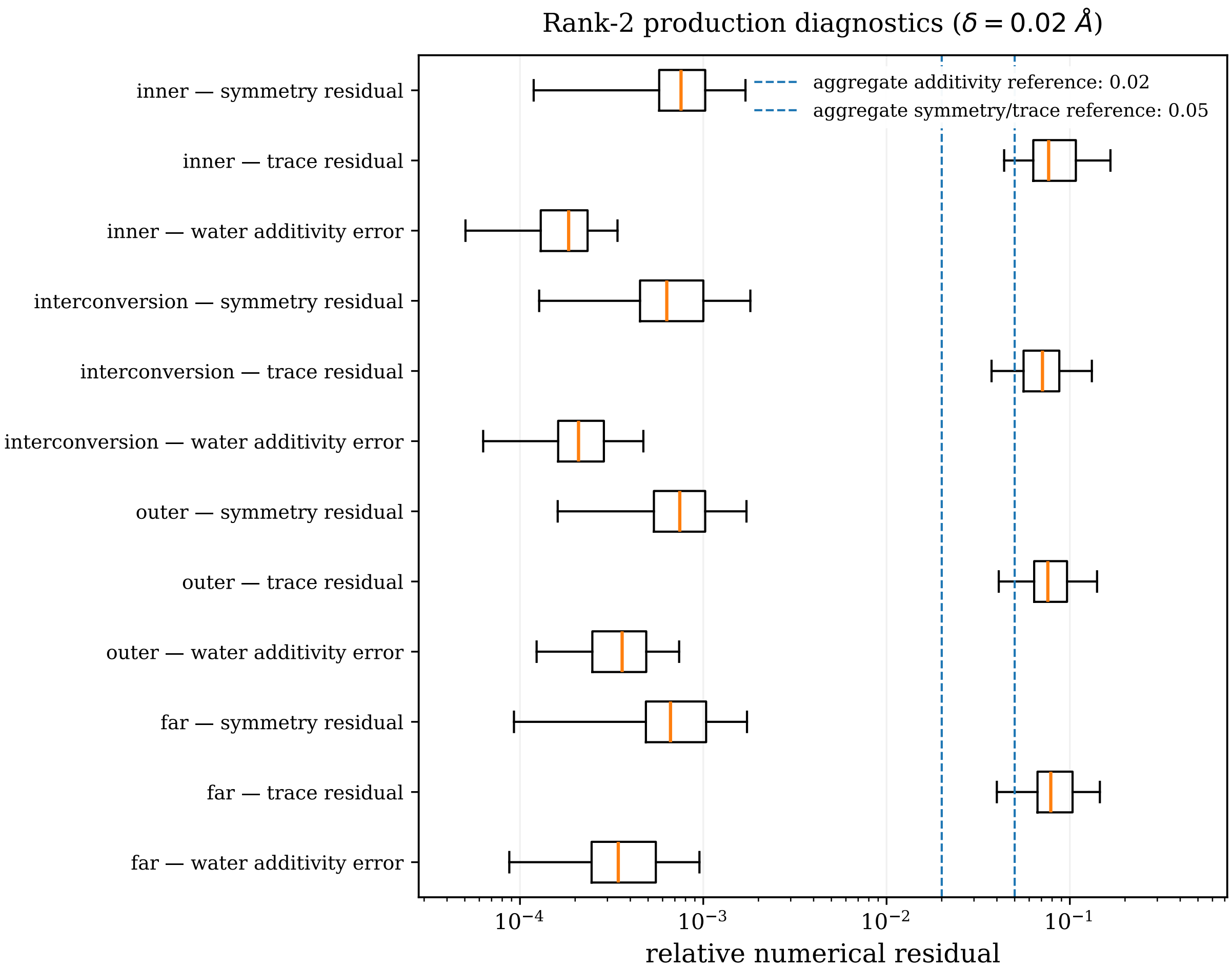

Figure S2: Numerical diagnostics for the production rank-2 calculation at $\delta = 0.02$ Å. Shown are the raw-gradient symmetry residual, raw Laplace-trace residual, and water-component additivity error for each conditional ensemble. Dashed lines reproduce the historical aggregate screening references used in the numerical audit. These references were applied to aggregate summary statistics, particularly medians, and are not per-snapshot pass/fail thresholds. The trace diagnostic is the sole rank-2 quantity exceeding its conservative aggregate reference; symmetry, additivity, and finite-difference convergence remain substantially smaller.

S3 Numerical validation of the rank-3 odd-parity tensor

S3.1 Finite-difference construction and STF projection

The second spatial derivative of the electric field was evaluated as

$$H_{ijk} = \frac{\partial^2 E_i}{\partial x_j \partial x_k} = -\frac{\partial^3 V}{\partial x_i \partial x_j \partial x_k}. \tag{S7}$$

Repeated derivative directions were obtained from

$$H_{ijj} \simeq \frac{E_i(+h\mathbf{e}_j) - 2E_i(0) + E_i(-h\mathbf{e}_j)}{h^2}, \tag{S8}$$

while mixed derivatives were evaluated through the corresponding four-point central stencil,

$$\begin{aligned} H_{ijk} \simeq \frac{1}{4h^2}\Big[&E_i(+h\mathbf{e}_j, +h\mathbf{e}_k) - E_i(+h\mathbf{e}_j, -h\mathbf{e}_k) \\ &- E_i(-h\mathbf{e}_j, +h\mathbf{e}_k) + E_i(-h\mathbf{e}_j, -h\mathbf{e}_k)\Big], \qquad j \neq k. \end{aligned} \tag{S9}$$

The raw tensor was fully symmetrized over Cartesian index permutations to give $S^{(3)}$. With

$$v_k = \sum_i S^{(3)}_{iik}, \tag{S10}$$

the symmetric-traceless rank-3 tensor was constructed as

$$O^{(3)}_{ijk} = S^{(3)}_{ijk} - \frac{1}{5}\left(\delta_{ij} v_k + \delta_{ik} v_j + \delta_{jk} v_i\right), \tag{S11}$$

and the cubic coefficient used in the potential expansion was

$$A^{(3)}_{ijk} = -\frac{1}{6} O^{(3)}_{ijk}. \tag{S12}$$

This is an odd-rank electrostatic coefficient of the local potential and must not be identified with a Judd–Ofelt intensity parameter. The production step was $h = 0.04$ Å. Finite-difference convergence was tested at $h = 0.03$ and 0.06 Å for a validation subset containing 96 snapshot comparisons across the four conditional ensembles. Figure S3 shows excellent convergence. The pooled median relative differences are 1.39×10^{-3} for $h = 0.03$ versus 0.04 Å and 2.53×10^{-3} for $h = 0.04$ versus 0.06 Å; their 95th percentiles remain below 5×10^{-3}.

S3.2 Permutation symmetry, harmonic trace, STF projection, and additivity

The production rank-3 tensors were subjected to four independent numerical diagnostics: (i) the residual between the raw tensor and its fully permutation-symmetrized form; (ii) the magnitude of

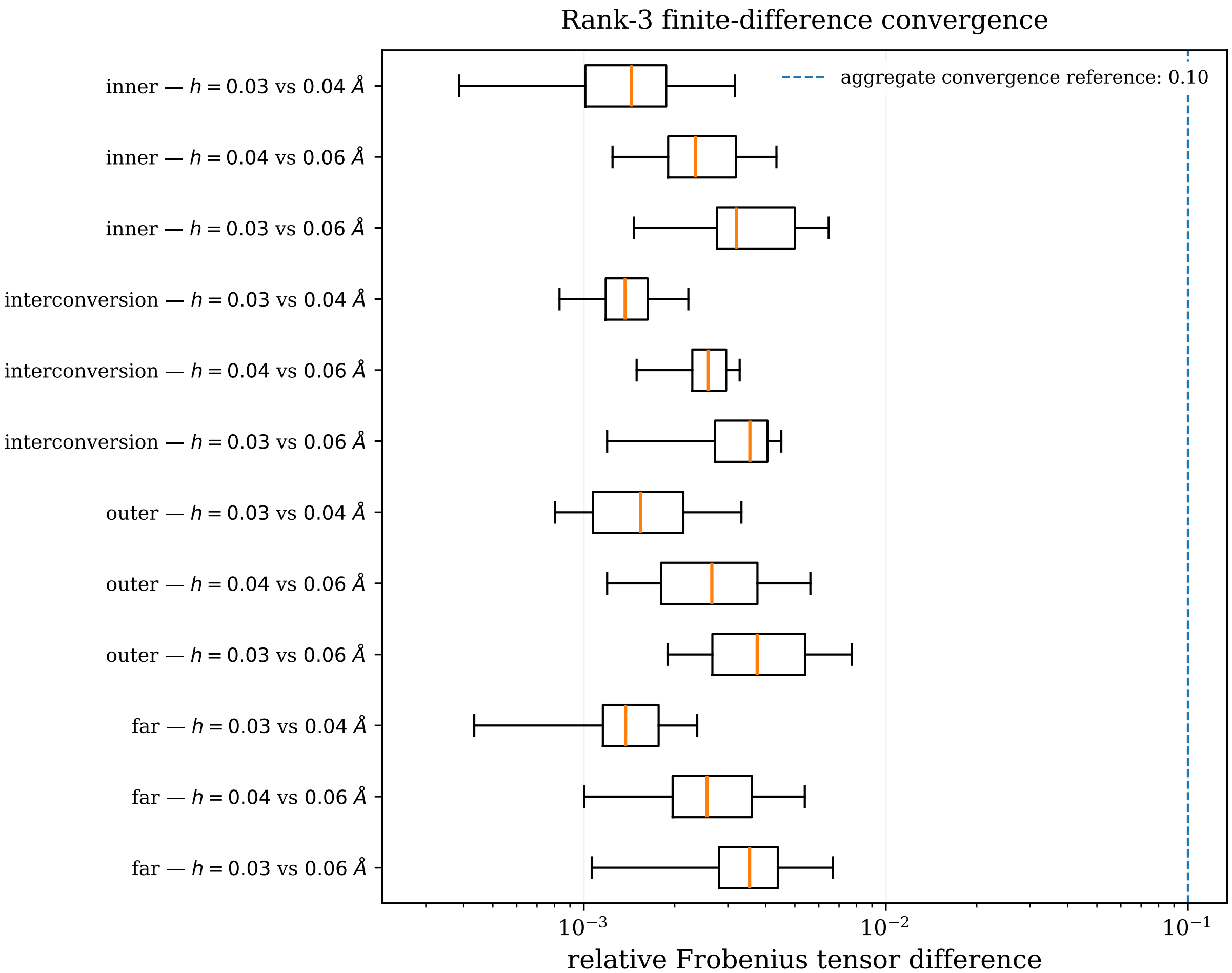

Figure S3: Finite-difference convergence of the rank-3 STF tensor. Box plots show snapshot-level relative Frobenius differences between $O^{(3)}$ tensors obtained with the indicated finite-difference steps. The $h = 0.04$ Å calculation is the production definition, whereas $h = 0.03$ and 0.06 Å were evaluated on the validation subset. The dashed line is the historical aggregate convergence screening reference of 0.10, not a per-snapshot rejection threshold.

the raw harmonic trace relative to the symmetrized tensor; (iii) the fraction of the symmetrized tensor removed by the STF projection; and (iv) the relative water-component additivity error obtained by comparing the directly evaluated water tensor with the total-minus-nitrate reconstruction.

All four quantities are small (Figure S4). The pooled medians of the permutation-symmetry residual, raw harmonic trace residual, STF-projection removed fraction, and water additivity error are 2.29×10^{-3}, 2.36×10^{-3}, 1.83×10^{-3}, and 5.55×10^{-4}, respectively. All four 95th percentiles remain below 5×10^{-3}, with the water-additivity diagnostic smaller still. Thus, the production $h = 0.04$ Å rank-3 STF tensor satisfies all predeclared numerical screening checks with substantial margin.

Table S3: Pooled numerical-validation statistics for the rank-3 analysis. Finite-difference convergence was evaluated for 96 validation comparisons; production diagnostics contain 480 snapshots.

Diagnostic	Median	95th percentile	Aggregate reference
$h = 0.03$ vs 0.04 Å	1.39×10^{-3}	2.61×10^{-3}	0.10
$h = 0.04$ vs 0.06 Å	2.53×10^{-3}	4.81×10^{-3}	0.10
$h = 0.03$ vs 0.06 Å	3.51×10^{-3}	6.56×10^{-3}	—
Permutation-symmetry residual	2.29×10^{-3}	4.29×10^{-3}	0.02
Raw harmonic trace residual	2.36×10^{-3}	4.91×10^{-3}	0.15
STF projection removed fraction	1.83×10^{-3}	3.81×10^{-3}	0.12
Water additivity error	5.55×10^{-4}	2.03×10^{-3}	0.02

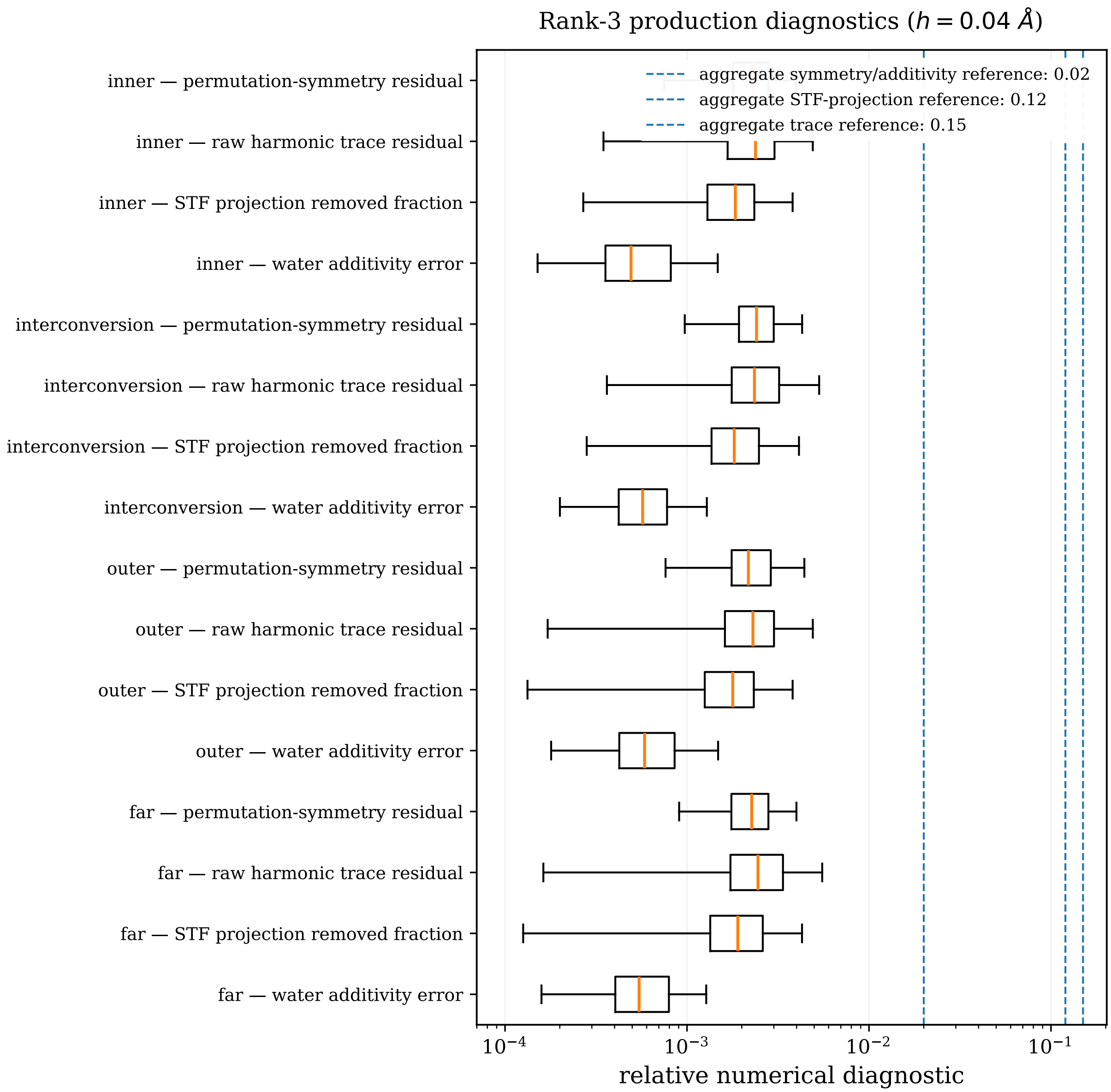

Figure S4: Numerical diagnostics for the production rank-3 analysis at $h = 0.04$ Å. Shown are the permutation-symmetry residual, raw harmonic trace residual, fraction removed by the STF projection, and water-component additivity error. Dashed lines reproduce the historical aggregate screening references used in the original audit; these were applied to aggregate statistics rather than as per-snapshot rejection thresholds. All four distributions remain well below their corresponding screening values.

S4 Molecular-frame decomposition of the rank-3 tensor

S4.1 Local molecular frame and transverse gauge

The Frobenius norm of $A^{(3)}$ measures the total magnitude of the odd-rank cubic electrostatic coefficient but does not specify how that magnitude is distributed among angular channels. To resolve this information, each Cartesian STF rank-3 tensor was transformed to an instantaneous molecular frame whose physically fixed axis is

$$\hat{\mathbf{z}} = \hat{\mathbf{r}}_{\mathrm{Eu}\to\mathrm{N}}, \tag{S13}$$

where N is the nitrogen atom of the selected nitrate. The transverse $\hat{\mathbf{x}}$ and $\hat{\mathbf{y}}$ directions form an arbitrary right-handed orthonormal gauge around this Eu–N axis. In the numerical implementation, a deterministic global reference direction least parallel to $\hat{\mathbf{z}}$ was projected into the transverse plane and then orthonormalized; the physical observables reported below do not depend on this auxiliary choice.

If R is the rotation matrix whose rows are the local basis vectors expressed in the laboratory frame, the local components are

$$A^{(3),\mathrm{loc}}_{abc} = R_{ai}R_{bj}R_{ck}A^{(3)}_{ijk}. \tag{S14}$$

S4.2 Orthonormal real tesseral rank-3 basis

The local STF tensor was projected onto a seven-dimensional real tesseral basis corresponding to $q = 0$, $|q| = 1$, $|q| = 2$, and $|q| = 3$. For complete reproducibility, the basis used in the analysis is given explicitly below. For a symmetric rank-3 tensor B_{ijk}, define its associated homogeneous cubic polynomial as

$$\mathcal{P}_B(x, y, z) = \sum_{ijk} B_{ijk}x_ix_jx_k. \tag{S15}$$

The seven tensors were normalized to unit Cartesian Frobenius norm, $B_\alpha : B_\alpha = 1$, and are represented by the following polynomials:

$$\mathcal{P}_0 = \frac{1}{\sqrt{10}}\left(2z^3 - 3zx^2 - 3zy^2\right), \tag{S16}$$

$$\mathcal{P}_{1c} = \sqrt{\frac{3}{20}}\,x\left(4z^2 - x^2 - y^2\right), \qquad \mathcal{P}_{1s} = \sqrt{\frac{3}{20}}\,y\left(4z^2 - x^2 - y^2\right), \tag{S17}$$

$$\mathcal{P}_{2c} = \sqrt{\frac{3}{2}}\,z\left(x^2 - y^2\right), \qquad \mathcal{P}_{2s} = \sqrt{\frac{3}{2}}\,(2xyz), \tag{S18}$$

$$\mathcal{P}_{3c} = \frac{1}{2}\,x\left(x^2 - 3y^2\right), \qquad \mathcal{P}_{3s} = \frac{1}{2}\,y\left(3x^2 - y^2\right). \tag{S19}$$

These normalizations refer to the Frobenius inner product of the corresponding Cartesian STF tensors, rather than to an independent normalization convention for scalar spherical harmonics. The basis is orthonormal by construction,

$$B_\alpha : B_\beta = \delta_{\alpha\beta}. \tag{S20}$$

The seven tesseral coefficients are therefore

$$q_\alpha = A^{(3),\mathrm{loc}} : B_\alpha, \qquad \alpha \in \{0, 1c, 1s, 2c, 2s, 3c, 3s\}, \tag{S21}$$

and Parseval completeness gives

$$\left\| A^{(3)} \right\|_F^2 = q_0^2 + q_{1c}^2 + q_{1s}^2 + q_{2c}^2 + q_{2s}^2 + q_{3c}^2 + q_{3s}^2. \tag{S22}$$

S4.3 Grouped angular-power fractions and gauge invariance

Because a rotation of the transverse (x, y) axes about $\hat{\mathbf{z}}$ mixes the cosine and sine members within a given $|q|$ pair, individual q_{mc} and q_{ms} coefficients depend on the transverse gauge. The physically robust descriptors used in the manuscript are instead the grouped powers

$$p_0 = \frac{q_0^2}{\|A^{(3)}\|_F^2}, \tag{S23}$$

$$p_1 = \frac{q_{1c}^2 + q_{1s}^2}{\|A^{(3)}\|_F^2}, \tag{S24}$$

$$p_2 = \frac{q_{2c}^2 + q_{2s}^2}{\|A^{(3)}\|_F^2}, \tag{S25}$$

$$p_3 = \frac{q_{3c}^2 + q_{3s}^2}{\|A^{(3)}\|_F^2}, \tag{S26}$$

which satisfy

$$p_0 + p_1 + p_2 + p_3 = 1. \tag{S27}$$

The sum of squares within each sine/cosine pair is invariant under the two-dimensional rotation induced by changing the transverse gauge. This invariance was also checked explicitly by repeating the complete decomposition after a 37° rotation of the local x/y axes while keeping $\hat{\mathbf{z}}$ fixed. The numerical audit is effectively at machine precision. Across all stored B5.6 components and configurations, the maximum relative Parseval residual is 5.50×10^{-16}, the maximum Parseval residual after the 37° transverse rotation is also 5.50×10^{-16}, and the maximum absolute change in any grouped $p_{|q|}$ power is 4.44×10^{-16}. Figure S5 shows the snapshot-level distributions for the total $A^{(3)}$ tensor.

S4.4 State-resolved angular redistribution

The total rank-3 magnitude alone conceals substantial changes in angular composition. Figure S6 shows the complete descriptive snapshot distributions of p_0, p_1, p_2, and p_3 for the four conditional

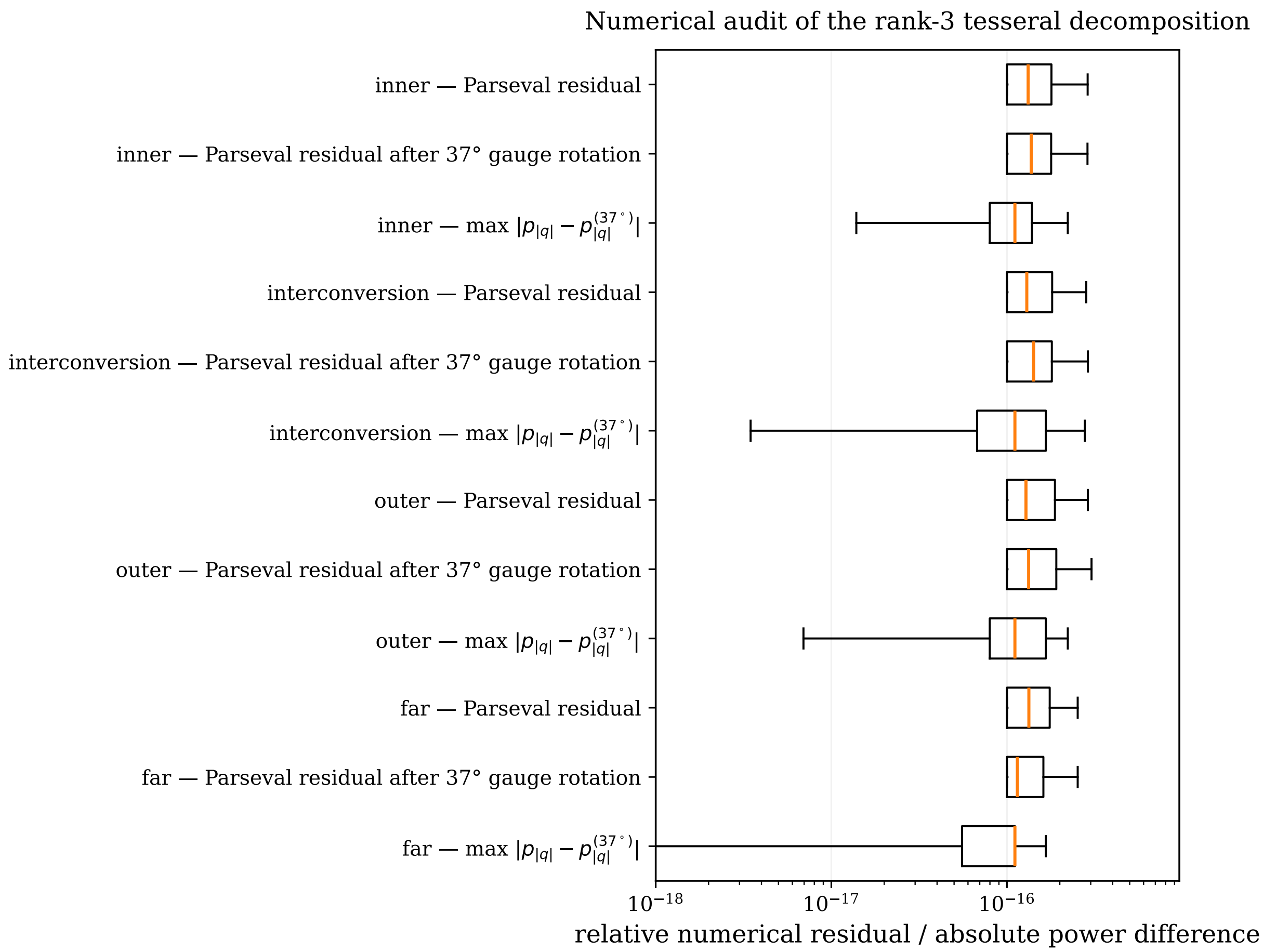

Figure S5: Numerical audit of the molecular-frame rank-3 tesseral decomposition for the total $A^{(3)}$ tensor. Shown are the Parseval residual before and after a 37° rotation of the arbitrary transverse x/y gauge and the maximum absolute change in the grouped powers $p_{|q|}$ under that rotation. All deviations are of order 10^{-16}, demonstrating numerical orthonormality/completeness and gauge invariance to machine precision.

ensembles. Boxes summarize the per-snapshot distributions and diamonds denote ensemble means; these graphical distributions are descriptive and should not be interpreted as independent replicate-based uncertainty intervals.

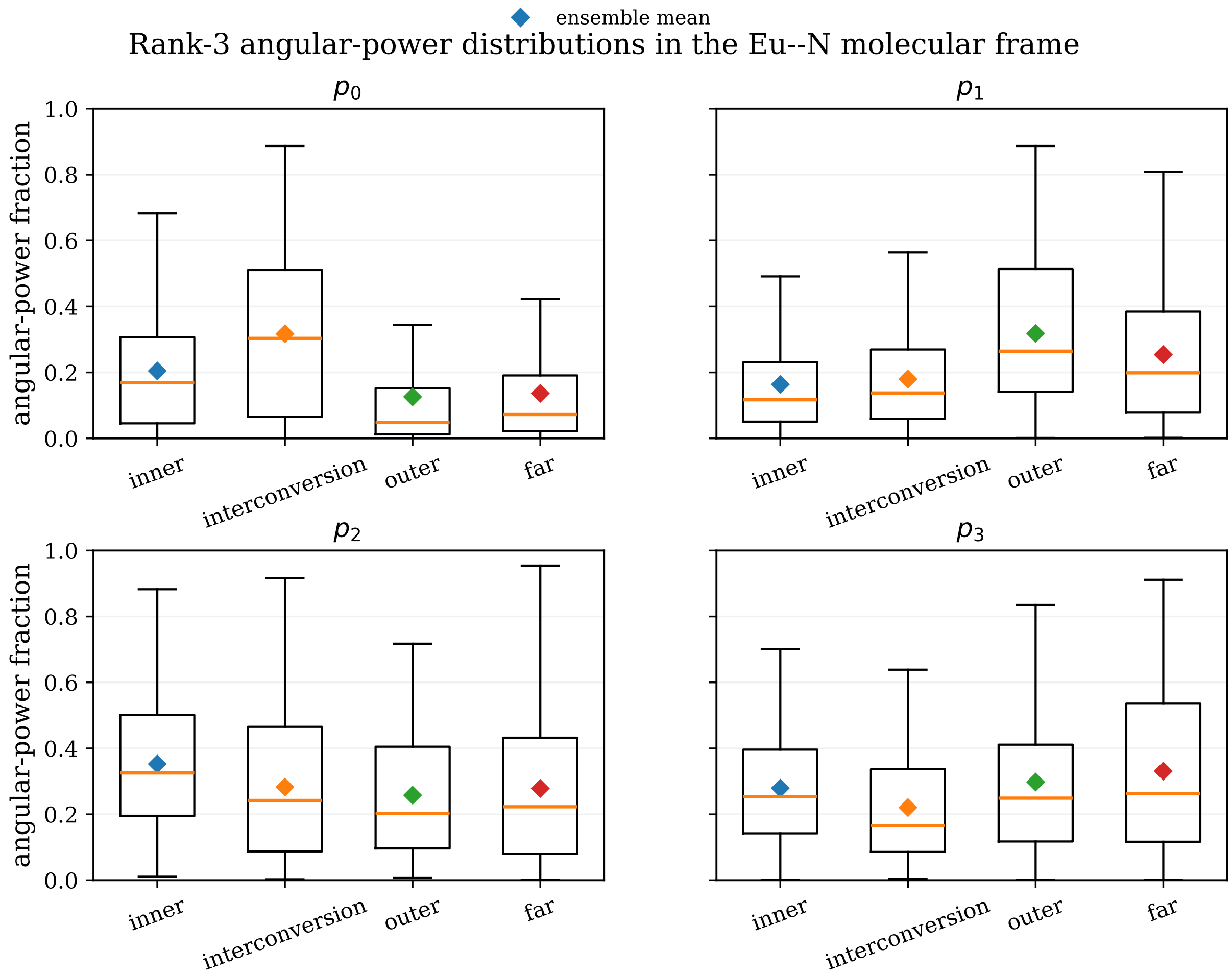

Figure S6: State-resolved distributions of the grouped rank-3 angular-power fractions in the Eu–N molecular frame. The four panels show p_0, p_1, p_2, and p_3 for the inner, interconversion, outer, and far conditional ensembles. Boxes summarize the snapshot distributions and diamonds mark ensemble means. The distributions are descriptive; source-aware inferential contrasts are reported separately. The broad interconversion-region ensemble is not interpreted as a transition-state ensemble, and the far state is an operational conditional reference rather than an infinite-dilution bulk state.

The state means are listed in Table S4. The inner and far ensembles have very similar total rank-3 magnitudes in the main analysis but differ in their angular composition. The broad interconversion ensemble is particularly distinctive, with enhanced p_0 and reduced p_3 relative to the far reference.

For the final source-cluster robustness analysis, the principal interconversion-minus-far contrasts are

$$\Delta p_0^{\text{interconversion}-\text{far}} = +0.180691, \qquad 95\% \text{ CI} = [+0.086434, +0.275221], \tag{S28}$$

with Cohen's $d = +0.833$, and

Table S4: State-resolved ensemble means of the grouped angular-power fractions for the total $A^{(3)}$ tensor. N_{src} denotes the number of distinct source trajectories represented in each conditional ensemble. The outer ensemble contains only two sources and therefore has intrinsically less robust source-aware uncertainty than the other ensembles.

State	N	N_{src}	p_0	p_1	p_2	p_3
inner	120	8	0.2046	0.1635	0.3526	0.2792
interconversion	120	12	0.3172	0.1798	0.2825	0.2204
outer	120	2	0.1261	0.3182	0.2579	0.2978
far	120	7	0.1366	0.2541	0.2784	0.3310

$$\Delta p_3^{\text{interconversion}-\text{far}} = -0.110520, \qquad 95\%\ \text{CI} = [-0.175643, -0.042859], \tag{S29}$$

with $d = -0.516$. By contrast, the corresponding change in p_2 is only $+0.0042$ with $d = 0.018$. Thus, the broad interconversion-region conditional ensemble is characterized by a selective redistribution of the odd-rank angular power rather than by a uniform increase of all angular channels. These descriptors characterize the symmetry-resolved structure of the classical local electrostatic potential. Odd-rank electrostatic terms have the parity required to participate in opposite-parity mixing, but the present $p_{|q|}$ values are not Judd–Ofelt parameters, oscillator strengths, or direct measures of emission intensity. In particular, no quantitative mapping from p_0 (or any other grouped power) to Ω_2 is assumed.

S5 Periodic-to-cluster electrostatic embedding validation

S5.1 Finite electrostatic embeddings and periodic reference

The electronic-structure calculations require a finite electrostatic representation of the condensed-phase environment, whereas the molecular dynamics and electrostatic-tensor analyses use periodic PPPM electrostatics. The finite embedding was therefore validated explicitly against the periodic environment before being used for multireference calculations. The QM region contains Eu^{3+}, the selected nitrate, and the prescribed first-shell waters. The external point-charge embedding always contains the two non-selected nitrates, while external TIP4P-Ew waters were included according to an O_w–Eu radial cutoff of 6, 8, 10, or 12 Å. The corresponding embeddings are denoted R6, R8, R10, and R12. The *FULL* embedding contains all external waters in the central minimum-image simulation cell. These finite embeddings are compact Eu-centered cluster models; they are not assumed a priori to be identical to the periodic PPPM/Ewald environment.

For the 32 composition-controlled configurations used to define the quantum design set, the periodic environment field at Eu was evaluated after electrostatically removing exactly the charges belonging to the QM region, while retaining Eu^{3+} as the field probe. The reference field is denoted

$$\mathbf{E}_{\text{env}}^{\text{PPPM}}. \tag{S30}$$

Each finite embedding field $\mathbf{E}_{\text{emb}}$ was then compared with the same periodic reference using the

absolute vector residual

$$\Delta E = \left\| \mathbf{E}_{\text{emb}} - \mathbf{E}_{\text{env}}^{\text{PPPM}} \right\|. \tag{S31}$$

The practical screening criteria adopted before analysis were a median $\Delta E \leq 0.03$ V Å^{-1} and a median directional cosine of at least 0.95. Figure S7 shows that increasing the explicit environment strongly reduces the rank-1 periodic residual from R6 to the largest finite models, but neither R12 nor FULL satisfies the absolute 0.03 V Å^{-1} criterion. R12 and FULL have median residuals of 0.1048 and 0.1064 V Å^{-1}, respectively, while their median directional cosines are 0.9853 and 0.9841. Thus, the large finite embeddings reproduce the direction of the periodic field well but retain a material missing long-range contribution in its vector magnitude.

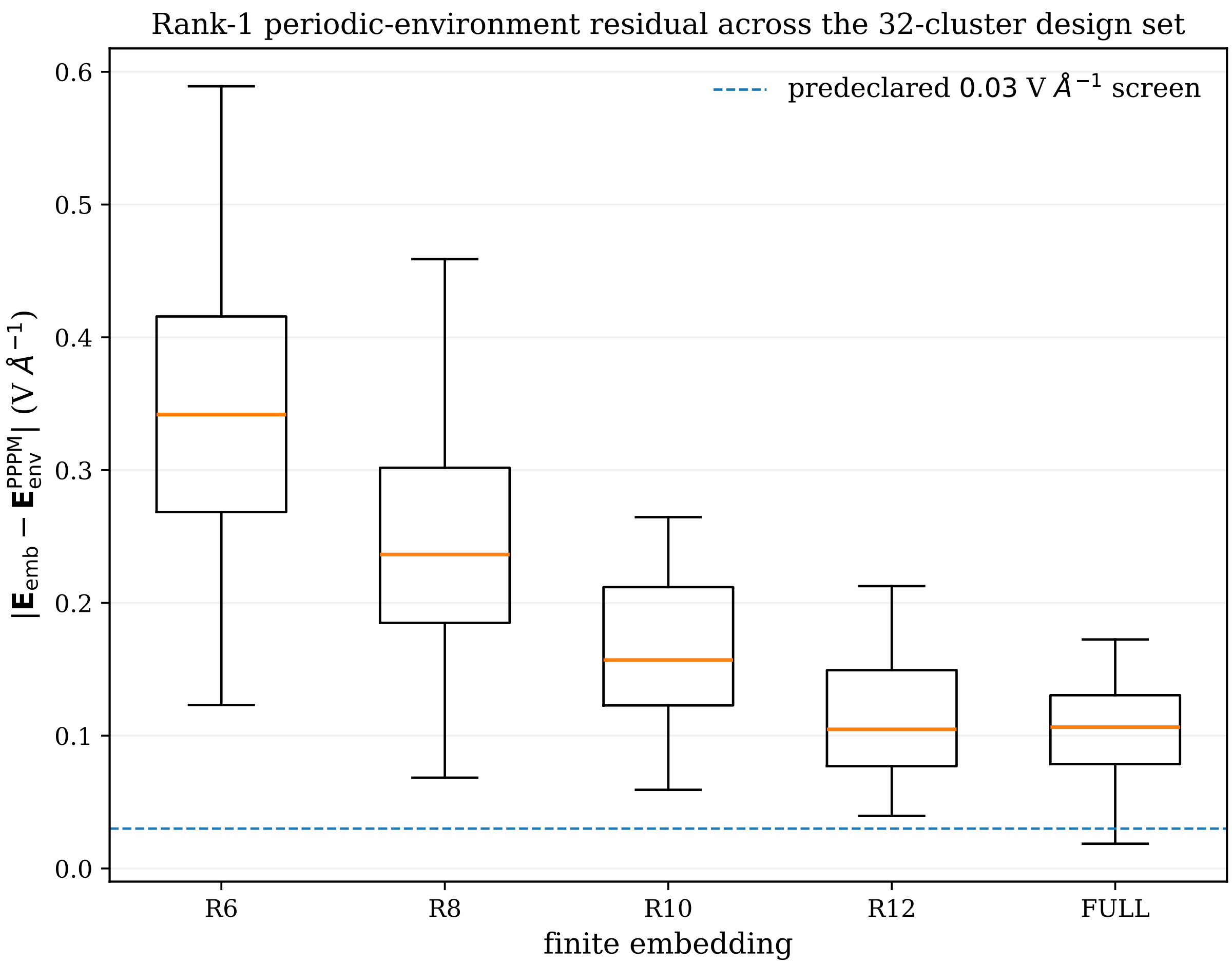

Figure S7: Rank-1 periodic-environment residual for the finite electrostatic embeddings over the 32-configuration quantum design set. R6–R12 contain external waters within the indicated Eu-centered radial cutoff, whereas FULL contains all central-cell external waters; the two non-selected nitrate ions are retained in every embedding. The dashed line marks the redeclared practical median screening value of 0.03 V Å^{-1} and is not a per-configuration rejection threshold. The non-monotonic R12/FULL medians should not be interpreted as evidence that R12 is a more complete electrostatic model: FULL is the complete central-cell embedding and also has the smaller 95th-percentile residual.

Table S5: Global rank-1 comparison of the finite point-charge embeddings with the periodic PPPM environment over the 32-configuration design set.

Embedding	Median ΔE (V Å^{-1})	$p_{95}(\Delta E)$ (V Å^{-1})	Median relative residual	Median $\cos\theta$	Median angle (deg)
R6	0.3418	0.5735	0.7071	0.8194	34.96
R8	0.2364	0.3892	0.4684	0.9164	23.60
R10	0.1570	0.2495	0.2939	0.9735	13.21
R12	0.1048	0.1990	0.2353	0.9853	9.80
FULL	0.1064	0.1572	0.2242	0.9841	10.24

S5.2 Physical STF rank-2 correction and spatial variation

A homogeneous correction of the rank-1 field would be sufficient only if the missing periodic contribution were essentially spatially uniform over the chemically relevant Eu-centered region. This question was tested on an eight-configuration mechanistic subset containing one central and one structurally diverse configuration from each conditional ensemble. Because the raw finite-difference PPPM gradient contains a non-negligible isotropic trace residual, the physical comparison was performed using the same symmetric-traceless rank-2 projection adopted throughout the main analysis,

$$T^{(2)} = \mathrm{sym}(G) - \frac{\mathrm{Tr}[\mathrm{sym}(G)]}{3} I. \tag{S32}$$

The missing periodic rank-2 contribution is therefore

$$\Delta T^{(2)} = T^{(2),\mathrm{PPPM}}_{\mathrm{env}} - T^{(2)}_{\mathrm{emb}}. \tag{S33}$$

A first-order estimate of the field variation generated by this missing STF term over a Eu-centered radius R is

$$\delta E_R \lesssim R\,\|\Delta T^{(2)}\|_2. \tag{S34}$$

The predeclared first-shell-scale diagnostic used $R = 3$ Å and the same 0.03 V Å^{-1} practical tolerance used for rank 1. For FULL, the physical STF relative residual is 0.0786 with median tensor cosine 0.9978. The estimated median variation is 0.0136 V Å^{-1} at 1 Å, but increases to 0.0407 V Å^{-1} over 3 Å, with a 95th percentile of 0.0632 V Å^{-1}. Thus, a homogeneous field alone is an adequate Eu-local approximation on approximately a 1-Å scale, but it does not satisfy the predeclared criterion over the broader first-shell scale. This result motivates an explicit rank-2 correction rather than a field-only adjustment.

S5.3 Periodic preservation of the odd-parity rank-3 tensor

The same eight-configuration subset was used to determine whether the finite embedding also required an explicit correction to the odd-parity rank-3 tensor. The periodic and finite-embedding

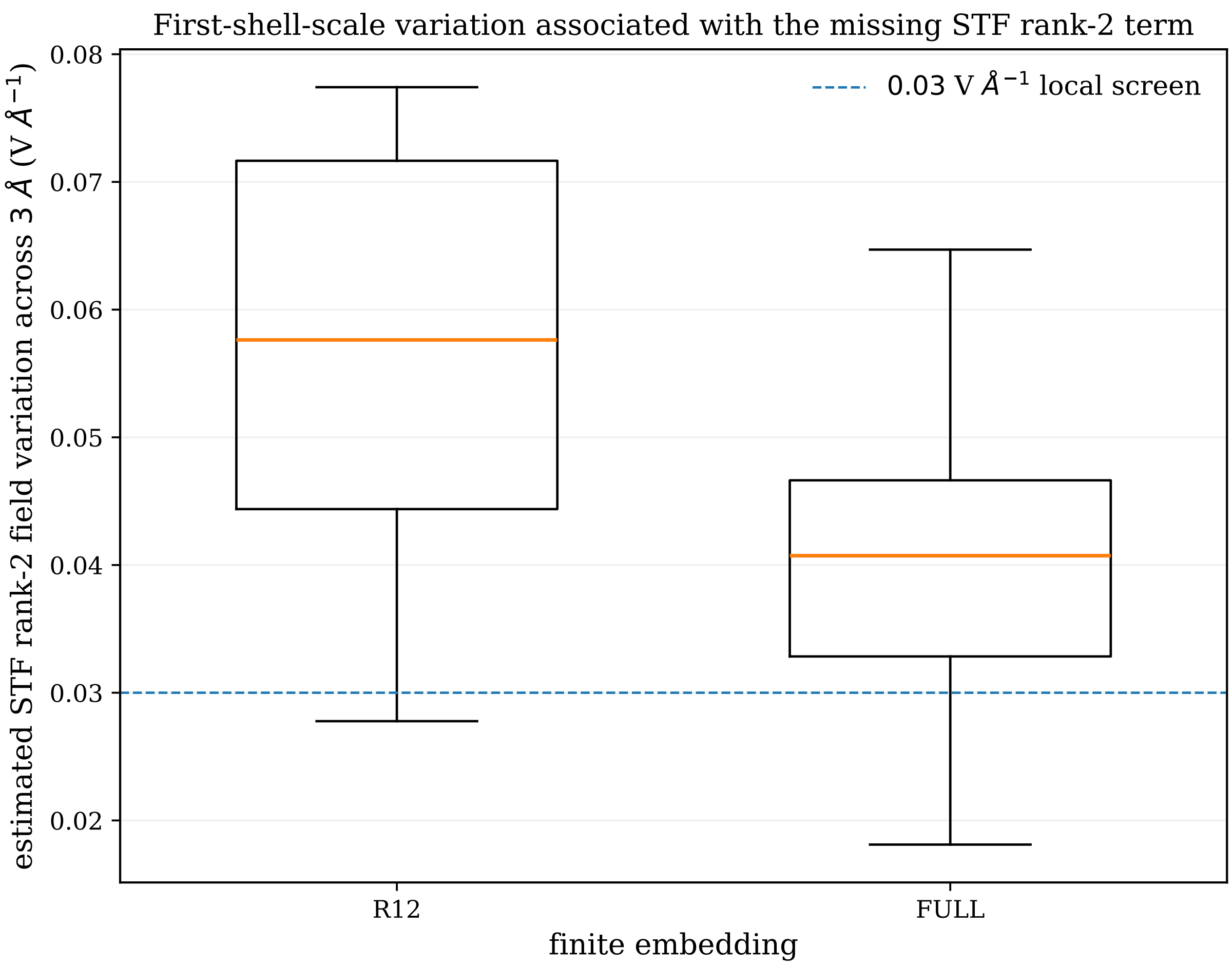

Figure S8: First-shell-scale field variation associated with the missing periodic STF rank-2 contribution for the R12 and FULL embeddings in the eight-configuration mechanistic subset. The ordinate is the first-order estimate $3\,\|\Delta T^{(2)}\|_2$. The dashed line marks the predeclared 0.03 V Å^{-1} local screening value. The physical STF projection is used here; the historical raw-gradient diagnostic is not used for the final rank-2 interpretation because it contains the isotropic PPPM trace artifact removed by Eq. S32.

tensors were compared using the relative Frobenius residual

$$\epsilon_3 = \frac{\left\| A^{(3)}_{\mathrm{emb}} - A^{(3)}_{\mathrm{PPPM}} \right\|_F}{\left\| A^{(3)}_{\mathrm{PPPM}} \right\|_F}, \tag{S35}$$

the tensor cosine, and the total-variation distance between the grouped angular powers p_0, p_1, p_2, p_3. The practical screening criteria were median $\epsilon_3 \leq 0.10$, median tensor cosine ≥ 0.995, and median angular total variation ≤ 0.05. FULL satisfies these criteria with substantial margin: the median relative rank-3 residual is 0.01836, its 95th percentile is 0.02794, the median tensor cosine is 0.999903, and the median angular total variation is 0.00907. The corresponding R12 values are also well within the same screening criteria. These results show that the complete central-cell embedding already preserves the periodic odd-parity rank-3 structure much more faithfully than it reproduces ranks 1 and 2.

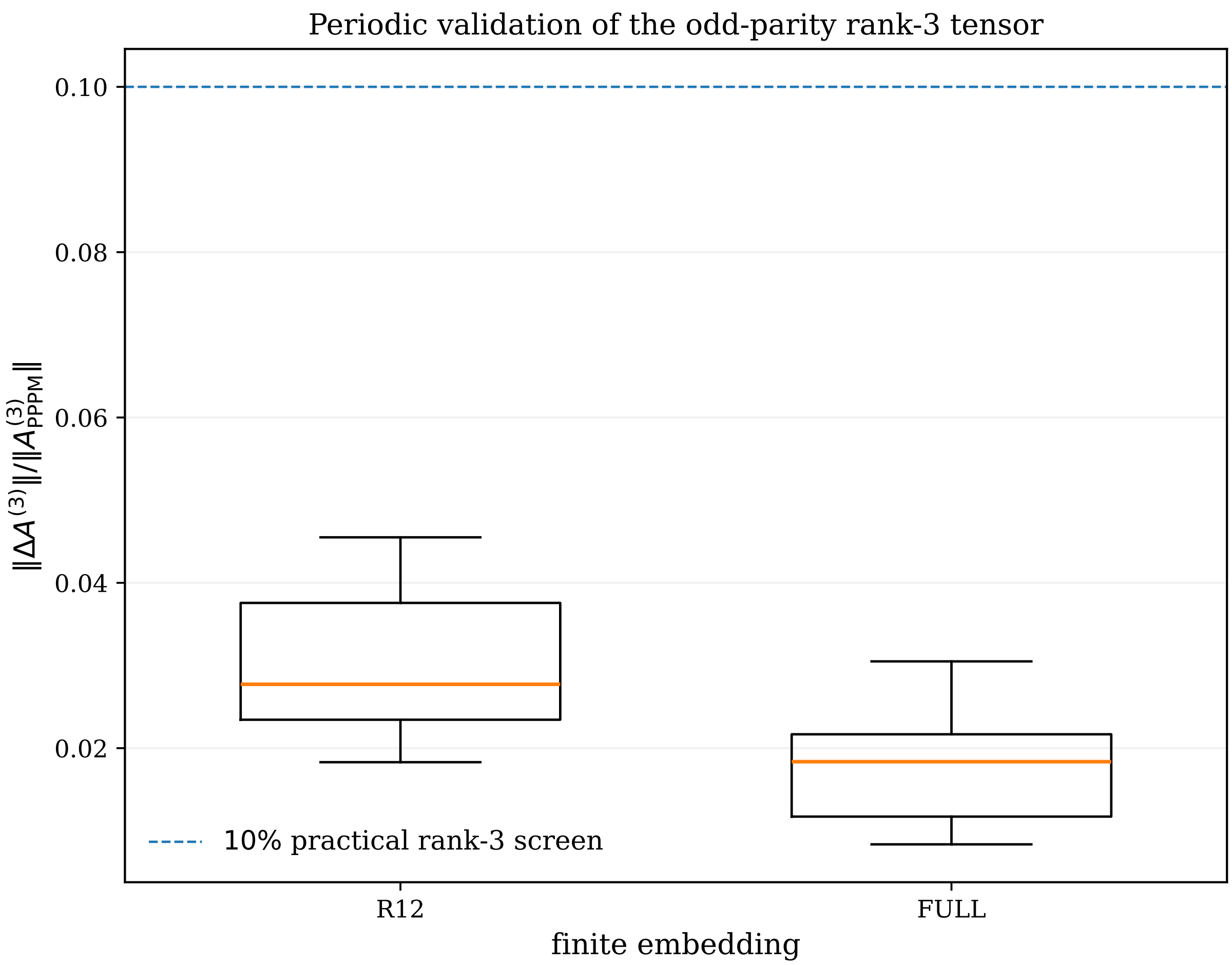

Figure S9: Periodic validation of the odd-parity rank-3 electrostatic tensor on the eight-configuration mechanistic subset. Box plots show the relative Frobenius residual of R12 and FULL with respect to the explicit periodic PPPM tensor. The dashed line marks the 10% practical rank-3 screening criterion. FULL gives a median relative residual of only 1.84%, with near-unity tensor cosine and small angular-power total variation.

Table S6: Rank-2 STF and rank-3 periodic validation on the eight-configuration mechanistic subset. The 1-Å rank-2 variation is supplementary; the predeclared first-shell-scale criterion is the 3-Å quantity.

Rank	Embedding	Relative residual	Tensor cosine	Secondary diagnostic	p_{95} secondary
2	R12	0.09156	0.99635	$\delta E_3 = 0.05763$	0.07578
2	FULL	0.07860	0.99777	$\delta E_3 = 0.04073$	0.06320
3	R12	0.02773	0.99974	$\mathrm{TV} = 0.01149$	0.02266
3	FULL	0.01836	0.99990	$\mathrm{TV} = 0.00907$	0.01899

S5.4 Neutral 26-site auxiliary shell

The preceding hierarchy motivates a deliberately low-rank periodic correction: rank 1 and STF rank 2 must be restored, whereas the already accurate FULL rank-3 tensor should remain unchanged. For each of the 32 configurations, 26 auxiliary sites were placed on a sphere of radius 15 Å centered on Eu. The fixed directions form a high-symmetry set consisting of

1. six Cartesian-axis directions, $\pm\hat{x}$, $\pm\hat{y}$, and $\pm\hat{z}$;
2. eight cube-corner directions, $(\pm 1, \pm 1, \pm 1)/\sqrt{3}$; and
3. twelve edge-center directions having one zero Cartesian component and the other two equal to $\pm 1/\sqrt{2}$.

For a given configuration the 26 charges q_a were determined from the linear constraints

$$\sum_{a=1}^{26} q_a = 0, \tag{S36}$$

$$\mathbf{E}_{\mathrm{aux}} = \mathbf{E}_{\mathrm{env}}^{\mathrm{PPPM}} - \mathbf{E}_{\mathrm{FULL}}, \tag{S37}$$

$$T_{\mathrm{aux}}^{(2)} = T_{\mathrm{env}}^{(2),\mathrm{PPPM}} - T_{\mathrm{FULL}}^{(2)}, \tag{S38}$$

$$A_{\mathrm{aux}}^{(3)} = 0. \tag{S39}$$

The independent constraint count is therefore $1 + 3 + 5 + 7 = 16$ for 26 unknown charges. The resulting 16×26 feature matrix has full row rank. After scaling each constraint row by its Euclidean norm, the minimum-L_2 solution was selected,

$$\mathbf{q}_{\mathrm{aux}} = \arg\min_{\mathbf{q}} \|\mathbf{q}\|_2 \quad \text{subject to} \quad A_s\mathbf{q} = \mathbf{b}_s. \tag{S40}$$

The scaled matrix condition number is 1.1676. The shell radius was deliberately chosen outside the complete QM clusters. The largest QM radial extent among the 32 configurations is 9.909 Å, giving a minimum radial separation of 5.091 Å from the 15-Å auxiliary shell. The auxiliary sites therefore remain outside the chemically active cluster and act as a smooth numerical correction rather than as additional physical ions.

S5.5 FULL+AUX numerical audit and interpretive boundary

The auxiliary-shell solution is numerically well conditioned and satisfies its defining constraints essentially to machine precision. Across all 32 configurations, the maximum residual net auxiliary charge is $3.89 \times 10^{-16}e$, the largest rank-1 reconstruction error is 2.97×10^{-16} V Å^{-1}, and the largest rank-2 reconstruction error is 3.61×10^{-16} V Å^{-2}. The maximum induced auxiliary rank-3 magnitude is only 2.16×10^{-18} V Å^{-3}, and the maximum change in $A^{(3)}$ between FULL+AUX and FULL is 2.02×10^{-17} V Å^{-3}.

The auxiliary charges themselves remain moderate: the median RMS charge is $0.1666e$, the median configuration-wise maximum magnitude is $0.3620e$, and the largest individual auxiliary charge over the complete 32-configuration set is $0.5048e$. The exact auxiliary-shell field also remains very close to its intended linear low-rank representation: the median nonlinear residual relative to $\Delta\mathbf{E} + \Delta T^{(2)}\mathbf{r}$ is only 4×10^{-6} V Å^{-1} at 1 Å and 1.03×10^{-4} V Å^{-1} at 3 Å.

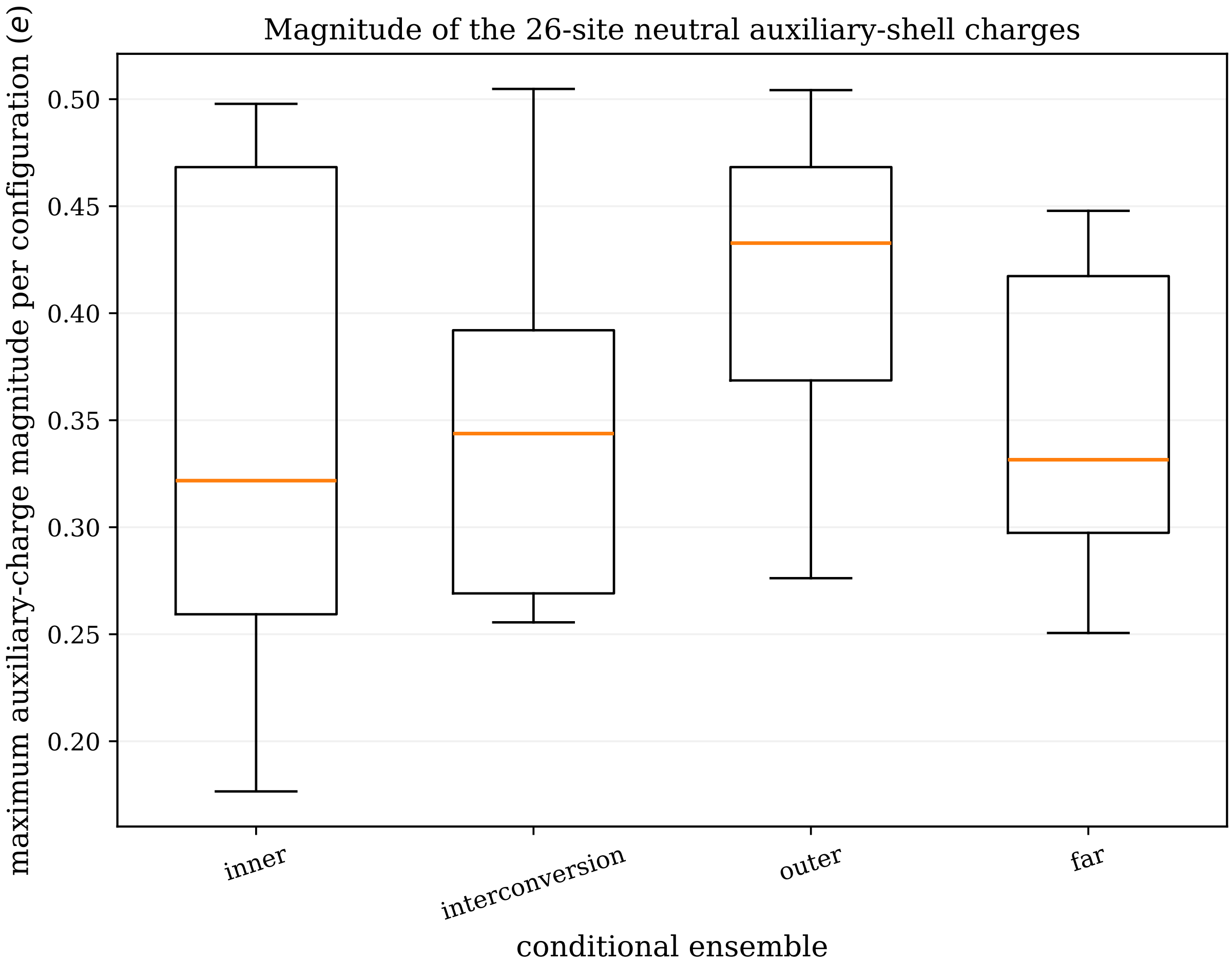

Figure S10: Distribution of the maximum absolute auxiliary-charge magnitude for each configuration, resolved by conditional ensemble. Each fitted configuration contains the same 26 fixed auxiliary sites on the 15-Å shell. The plot quantifies the strength of the numerical low-rank correction; the auxiliary sites are not physical atoms or ions.

The state-resolved target corrections are similarly moderate. The median $|\Delta\mathbf{E}|$ values are 0.0825, 0.0979, 0.1327, and 0.1121 V Å^{-1} for the inner, interconversion, outer, and far ensembles, respectively. The corresponding median $\|\Delta T^{(2)}\|_F$ values are 0.01536, 0.01506, 0.01644, and 0.01710 V Å^{-2}.

Table S7: Global numerical audit of the 26-site auxiliary-shell correction over the 32-configuration design set.

Quantity	Value
Shell radius	15.000 Å
Auxiliary sites per configuration	26
Scaled fit-matrix condition number	1.167592
Maximum $\lvert \sum q_{\mathrm{aux}} \rvert$	$3.89 \times 10^{-16} e$
Median RMS(q_{aux})	$0.166585e$
Median max $\lvert q_{\mathrm{aux}} \rvert$	$0.362006e$
Global max $\lvert q_{\mathrm{aux}} \rvert$	$0.504791e$
Maximum rank-1 fit error	2.97×10^{-16} V Å^{-1}
Maximum rank-2 fit error	3.61×10^{-16} V Å^{-2}
Maximum $\lVert A^{(3)}_{\mathrm{aux}} \rVert_F$	2.16×10^{-18} V Å^{-3}
Maximum FULL+AUX rank-3 change vs FULL	2.02×10^{-17} V Å^{-3}
Median nonlinear residual at 1 Å	4×10^{-6} V Å^{-1}
Median nonlinear residual at 3 Å	1.03×10^{-4} V Å^{-1}
Maximum QM radial extent	9.908823 Å
Minimum shell clearance	5.091177 Å

The outer ensemble consequently requires the largest median configuration-wise auxiliary charge magnitude ($0.4327e$), but remains within the overall range reported above. Two boundaries are essential for interpreting this construction. First, FULL+AUX reproduces the periodic rank-1 field and STF rank-2 tensor at Eu *by construction*; these machine-precision residuals are therefore fit-validation quantities, not independent tests of physical transferability. Second, explicit periodic rank-3 validation was performed only on the eight-configuration B6.7 mechanistic subset. The condition $A^{(3)}_{\mathrm{aux}} = 0$ is imposed analytically for all 32 fitted configurations so that the correction leaves the independently validated FULL rank-3 tensor unchanged. The auxiliary shell should thus be understood as a numerical low-rank boundary correction, not as a literal finite representation of the infinite periodic medium.

S6 Additional statistical robustness analyses

S6.1 Source-aware inference and configurational genealogy

The conditional electrostatic ensembles contain multiple snapshots from the same underlying trajectory segments. Treating all snapshots as independent replicates would therefore overstate the effective amount of independent sampling. The principal uncertainty analysis used the source identifier as the clustering unit. For each bootstrap realization, complete source trajectory segments were resampled with replacement, and the corresponding snapshot data were carried with the selected source. This procedure preserves the within-source dependence while propagating the observed between-source heterogeneity.

The final electrostatic data set contains 120 snapshots in each conditional ensemble. The inner, interconversion, outer, and far ensembles originate from 8, 12, 2, and 7 source identifiers, respectively (Table S8). The relatively small number of outer-state sources is therefore an intrinsic limitation of source-aware inference for that state and is not hidden by the larger snapshot count.

Table S8: Source composition of the final conditional electrostatic ensembles. The source identifier, rather than the individual snapshot, is the clustering unit used in the source-cluster bootstrap. The historical "transition" label in intermediate analysis files corresponds to the broad interconversion-region ensemble reported here.

Conditional ensemble	Snapshots	Source identifiers
inner	120	8
interconversion	120	12
outer	120	2
far	120	7

The source-cluster bootstrap addresses heterogeneity among the available source segments, but it should not be interpreted as a complete independence correction. In particular, it does *not* independently resample temporal structure within a source, and it cannot remove shared configurational genealogy between umbrella branches that descend from a common parent configuration. Separate parent-only versus child/reseeded sensitivity checks were used for the one-dimensional PMF topology; those tests are conceptually distinct from the source-cluster bootstrap. The far ensemble is likewise retained only as the operational 7.50–9.00 Å conditional reference used throughout the analysis. It is not interpreted as an infinite-dilution, asymptotic-bulk, or standard-state reference.

S6.2 Robustness of the rank-3 angular signature to source and spectator-nitrate controls

The principal interconversion-minus-far angular contrasts are

$$\Delta p_0 = +0.180691, \qquad 95\%\ \mathrm{CI} = [+0.086434, +0.275221], \tag{S41}$$

$$\Delta p_3 = -0.110520, \qquad 95\%\ \mathrm{CI} = [-0.175643, -0.042859]. \tag{S42}$$

The corresponding snapshot-level standardized effects are $d = +0.833$ and $d = -0.516$, respectively.

The total rank-3 norm, by contrast, changes only weakly between these two conditional ensembles, so the resolved signal is primarily an angular redistribution rather than an increase of the overall odd-rank magnitude. The leave-one-source-out calculation provides a direct sign-robustness diagnostic. Omitting each interconversion or far source in turn gives

$$\Delta p_0^{\mathrm{LOO}} \in [+0.158839, +0.202997], \qquad \Delta p_3^{\mathrm{LOO}} \in [-0.125844, -0.101689]. \tag{S43}$$

Thus, no single source identifier controls the signs of the two headline angular contrasts. Because two additional nitrate ions are present in the simulation cell, spectator-nitrate geometry was also treated explicitly as a sensitivity analysis. "Clean" configurations exclude a spectator nitrate N within 6 Å of Eu, whereas "selected-nearest" requires the tagged nitrate to be the nitrate whose N atom is closest to Eu. These filters are controls on conditioning; they are not replacements for the full conditional ensembles, and a selected-nearest far subset must not be described as a "corrected" far state.

Figure S11 and Table S9 show that the positive p_0 enrichment is retained under all of these controls. In particular, the clean, selected-nearest, and simultaneous clean+selected-nearest subsets give $\Delta p_0 = +0.1494$, $+0.1656$, and $+0.1471$, respectively, with source-cluster 95% intervals that remain entirely positive. The p_3 contrast also retains its negative point-estimate sign under every displayed control. Its interval becomes marginally unresolved in the clean subset alone, but remains resolved for the selected-nearest and clean+selected-nearest subsets. The deliberately crowded subset preserves both signs with larger effect magnitudes.

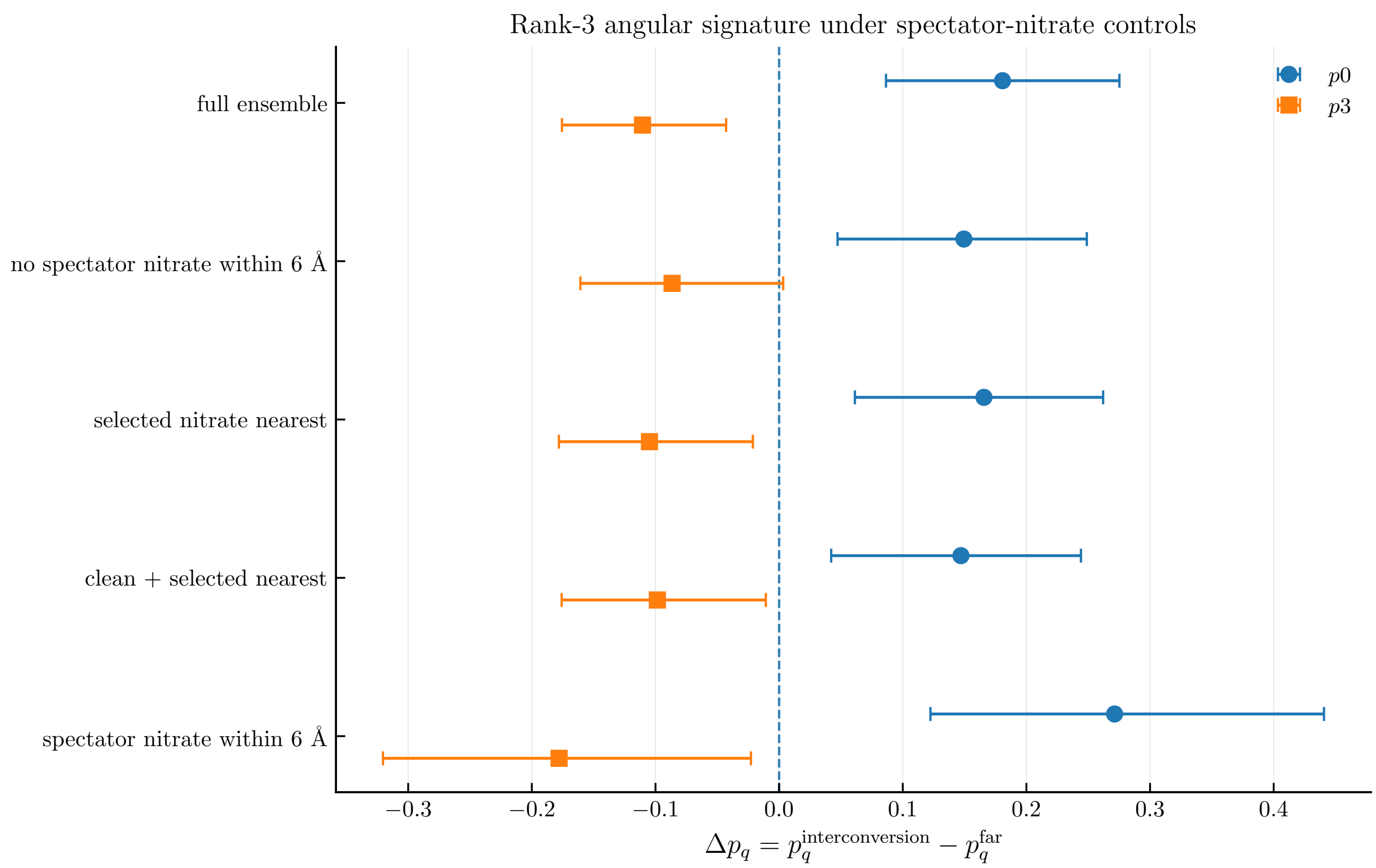

Figure S11: Sensitivity of the interconversion-minus-far rank-3 angular contrasts to spectator-nitrate and nitrate-identity controls. Symbols show $\Delta p_q = p_q^{\mathrm{interconversion}} - p_q^{\mathrm{far}}$ and error bars are source-cluster bootstrap 95% confidence intervals. "Clean" excludes configurations with a spectator nitrate N within 6 Å of Eu;"selected nitrate nearest" requires the tagged nitrate N to be the closest nitrate N to Eu. These restricted subsets are sensitivity analyses, not alternative definitions of the production conditional ensembles.

Table S9: Sensitivity of the principal interconversion-minus-far angular contrasts to spectator-nitrate and nitrate-identity controls. Intervals are source-cluster bootstrap 95% confidence intervals.

Subset	Δp_0 [95% CI]	Δp_3 [95% CI]
Full ensemble	$+0.1807$ $[+0.0864, +0.2752]$	-0.1105 $[-0.1756, -0.0429]$
No spectator nitrate within 6 Å	$+0.1494$ $[+0.0473, +0.2488]$	-0.0866 $[-0.1607, +0.0033]$
Selected nitrate nearest	$+0.1656$ $[+0.0613, +0.2621]$	-0.1048 $[-0.1781, -0.0212]$
Clean + selected nearest	$+0.1471$ $[+0.0421, +0.2441]$	-0.0985 $[-0.1758, -0.0107]$
Spectator nitrate within 6 Å	$+0.2713$ $[+0.1224, +0.4407]$	-0.1780 $[-0.3204, -0.0228]$

A complementary within-state fixed-effect analysis further separates the angular observables from the direct electrostatic contribution of the spectator nitrate. Within the interconversion ensemble, binary spectator crowding gives fixed-effect estimates $\beta_{p_0} = +0.0473$ with 95% interval $[-0.0630, +0.1623]$ and $\beta_{p_3} = -0.0109$ with interval $[-0.1028, +0.0762]$. Using continuous spectator approach instead gives $\beta_{p_0} = +0.00885$ $[-0.01629, +0.03620]$ and $\beta_{p_3} = -0.00599$ $[-0.02756, +0.01163]$. In contrast, the spectator-nitrate rank-3 contribution itself increases strongly as the spectator approaches Eu. Thus, spectator nitrate position modulates the local electrostatic decomposition, but the headline interconversion-region p_0/p_3 signature is not explained by a simple within-source spectator-distance effect.

S6.3 Thermodynamic stress test of the transferred Eu–nitrate C_4 terms

The Eu–nitrate C_4 coefficients were transferred rather than directly fitted for the present Eu^{3+}/TIP4P-Ew/nitrate model. Their impact on the local PMF topology was therefore tested by paired reweighting at fixed sampled geometries. The published Eu–water C_4 coefficient was held fixed. Coherent factors

$$\lambda = 0.8,\ 0.9,\ 1.0,\ 1.1,\ 1.2 \tag{S44}$$

were applied simultaneously to the transferred Eu–N(NO_3) and Eu–O(NO_3) C_4 terms. Additional ±20% one-at-a-time perturbations were used only to decompose the local sensitivity between the two transferred terms. The sparse configurations used for the paired Hamiltonian response were not treated as independent production PMFs. Instead, the differential free-energy response was anchored to the dense production PMF. Temporal uncertainty in the sparse reweighting used 1000 bootstrap realizations with 10-ps blocks. The dense production baseline remained the official B2.9 PMF. The propagated intervals reported for this stress test additionally combine the differential response with the pre-existing dense-PMF uncertainty. Because the dense and sparse bootstrap draws were not synchronized, their covariance is not modeled; the propagated intervals are therefore a conservative diagnostic rather than a new production-PMF uncertainty analysis. The coherent perturbations give

$$\Delta W_{\mathrm{inner-out}} = 3.517,\ 3.046,\ 2.575,\ 2.104,\ 1.635\ \mathrm{kcal\,mol^{-1}} \tag{S45}$$

for increasing λ from 0.8 to 1.2. All propagated 95% intervals remain wholly above zero (Figure S12 and Table S10), including the most strongly attractive coherent perturbation,

$$\Delta W_{\mathrm{inner-out}}(\lambda = 1.2) = 1.634760 \quad [1.278146, 1.992867]\ \mathrm{kcal\,mol^{-1}}. \tag{S46}$$

The predeclared closure condition—a positive 95% lower bound for every coherent ±20% stress-test Hamiltonian—is therefore satisfied.

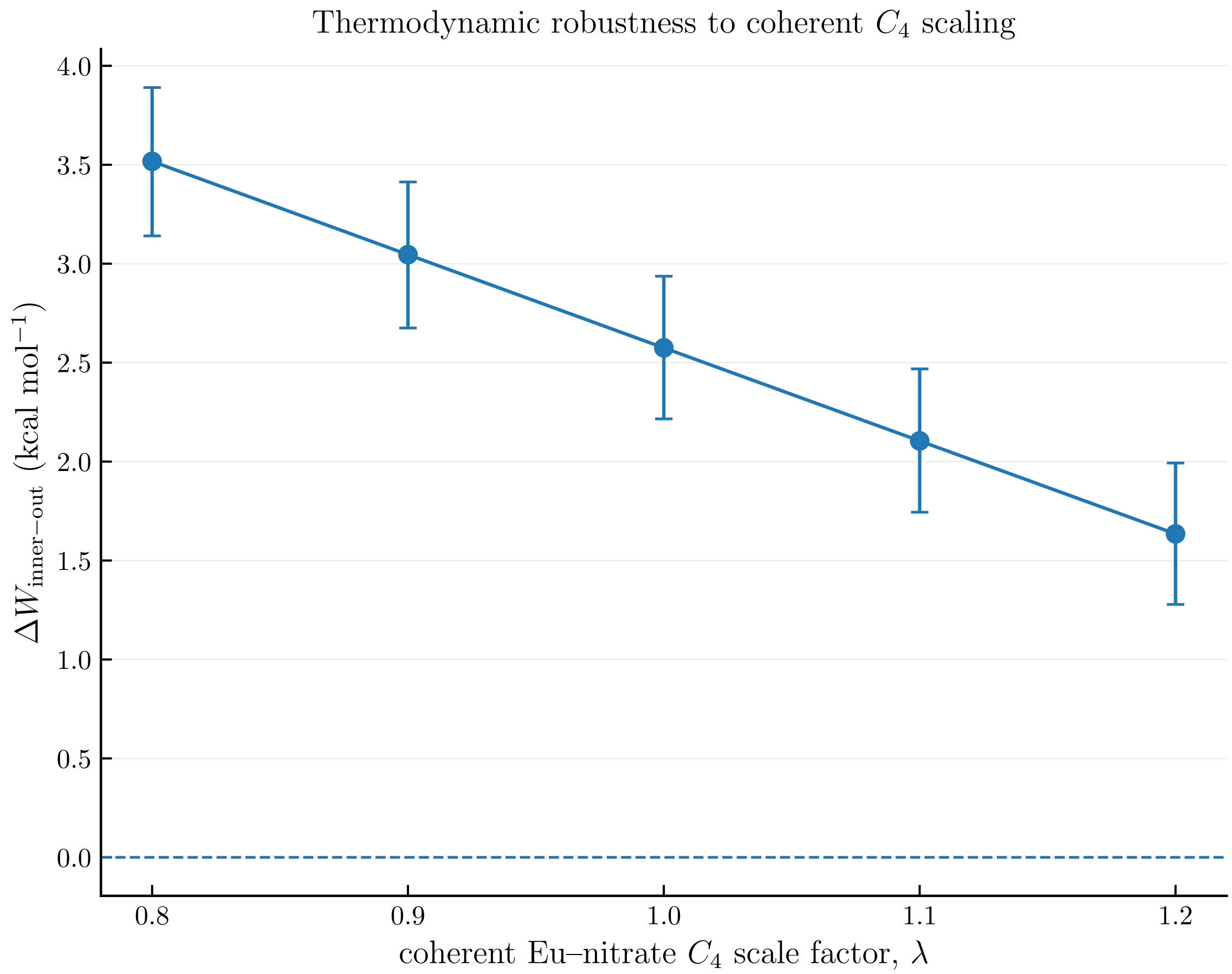

Figure S12: Thermodynamic stress test of the transferred Eu–nitrate C_4 terms. Both transferred nitrate C_4 coefficients are multiplied by the same coherent scale factor λ, while the published Eu–water coefficient is unchanged. Symbols show the dense-PMF-anchored $\Delta W_{\mathrm{inner-out}}$ values and error bars show the propagated 95% intervals. The dashed horizontal line marks zero. The ±20% range is a robustness stress test, not a statistically calibrated uncertainty distribution for the force-field parameters.

Across the coherent stress test, the symmetric local sensitivity is

$$\frac{d\Delta W_{\mathrm{inner-out}}}{d\lambda} = -4.697187 \quad [-4.863185, -4.469184]\ \mathrm{kcal\,mol^{-1}}. \tag{S47}$$

The one-at-a-time decomposition gives mean sensitivities of $-3.641982\ \mathrm{kcal\,mol^{-1}}$ for Eu–O(NO_3) and $-1.055034\ \mathrm{kcal\,mol^{-1}}$ for Eu–N(NO_3), with 95% intervals $[-3.754012, -3.486668]$ and $[-1.114218, -0.978291]$, respectively. On an absolute-sensitivity basis, the Eu–O(NO_3) term accounts for 77.54% on average (95% interval 77.06–78.16%), leaving approximately 22.46% for the Eu–N term. The magnitude of the local free-energy separation is therefore sensitive to the transferred induction terms, but its sign and local basin ordering are robust throughout the predeclared ±20% diagnostic range.

Table S10: Dense-PMF-anchored inner-minus-outer free-energy contrast under coherent scaling of the two transferred Eu–nitrate C_4 coefficients.

λ	$\Delta W_{\mathrm{inner-out}}$ (kcal mol^{-1})	Propagated 95% interval (kcal mol^{-1})	$P(\Delta W > 0)$
0.8	3.517243	[3.140521, 3.890169]	1.000
0.9	3.045741	[2.675092, 3.412980]	1.000
1.0	2.574812	[2.215742, 2.936717]	1.000
1.1	2.104486	[1.744224, 2.468368]	1.000
1.2	1.634760	[1.278146, 1.992867]	1.000

S6.4 Matched-MD configurational stress test of the angular signature

The fixed-geometry B2.10 analysis tests the thermodynamic sensitivity of the PMF but cannot determine whether changing C_4 materially alters the configurations that carry the rank-3 angular signature. A complementary matched-MD diagnostic was therefore carried out at coherent $\lambda = 0.8$, 1.0, and 1.2. For a given source configuration, the same newly randomized velocity seed was used at all three λ values, matching the branches across Hamiltonians while retaining the shared configurational ancestry of the parent umbrella source. Each branch was propagated for 200 ps at 298.15 K. The first 50 ps were discarded and the final 150 ps were used for conditional selection. The broad historical interconversion selector,

$$3.85 \leq r_{\mathrm{Eu-N}} \leq 4.30 \ , \tag{S48}$$

was applied without water-coordination or denticity restrictions. It yields 70, 58, and 40 interconversion configurations at $\lambda = 0.8$, 1.0, and 1.2, respectively. The corresponding far conditional ensembles were source-balanced to 119 configurations at each Hamiltonian. For these frozen geometries, the total electrostatic rank-3 tensor was re-evaluated with the established $h = 0.04$ Å stencil and PPPM/TIP4P accuracy of 10^{-7}. The frozen-geometry electrostatic rerun contains no Lennard–Jones, C_4, umbrella, or dynamics term. Consequently, C_4 can affect the rank-3 angular powers in this test only *indirectly*, through the configurations generated by the matched MD, and not through the electrostatic tensor evaluation at fixed geometry.

Because the numbers of interconversion candidates are strongly unequal among source branches, the primary B2.11 uncertainty estimate differs from the source-cluster procedure used for the main production electrostatic contrasts. It uses equal source weights combined with a hierarchical source-resampling and 10-ps temporal-block resampling within source, with 20 000 bootstrap realizations. A B5-compatible source/snapshot bootstrap and leave-one-source-out calculations were retained as complementary diagnostics. The $\lambda = 1.0$ branch was the predeclared methodological control; interpretation of the $\lambda = 0.8$ and 1.2 extremes was gated by successful reproduction of the positive Δp_0 signature at $\lambda = 1.0$.

The resulting equal-source contrasts are

$$\Delta p_0(\lambda = 0.8) = +0.254033, \qquad 95\%\ \mathrm{CI} = [+0.109943, +0.400298], \tag{S49}$$
$$\Delta p_0(\lambda = 1.0) = +0.274435, \qquad 95\%\ \mathrm{CI} = [+0.166127, +0.368318], \tag{S50}$$
$$\Delta p_0(\lambda = 1.2) = +0.187969, \qquad 95\%\ \mathrm{CI} = [+0.031202, +0.351427]. \tag{S51}$$

All three intervals remain positive, and every equal-source leave-one-source-out calculation also retains the positive sign. The LOO ranges are $[+0.219169, +0.302737]$, $[+0.256013, +0.310639]$, and $[+0.133940, +0.234985]$ for $\lambda = 0.8$, 1.0, and 1.2, respectively.

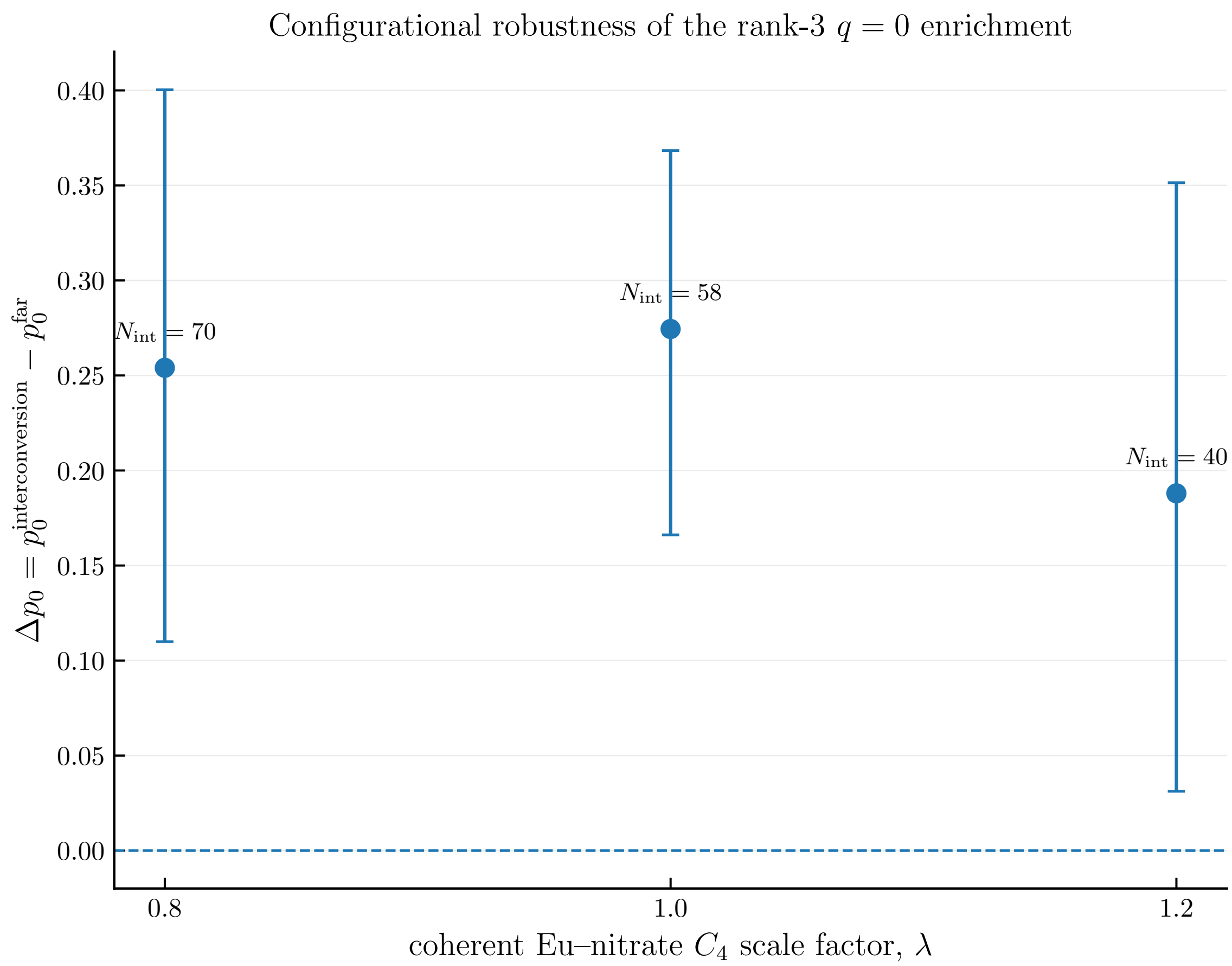

Figure S13: Matched-MD configurational stress test of the interconversion-minus-far rank-3 $q = 0$ angular-power contrast. Symbols show equal-source Δp_0 values and error bars show the primary equal-source hierarchical source + 10-ps temporal-block 95% intervals. Labels report the number of retained interconversion configurations; each far ensemble contains 119 configurations. The dashed line marks zero. The calculation is a targeted robustness diagnostic, not an independent production characterization of the conditional-state distributions.

No monotonic dependence of Δp_0 on the C_4 scale factor is inferred from these three Hamiltonians. The result is instead a robustness statement: the qualitative interconversion-region enrichment of the $q = 0$ rank-3 channel survives substantial configurational perturbation of the transferred Eu–nitrate induction terms. Because none of the interconversion samples reaches the conservative 120-configuration target, B2.11 is retained as a targeted configurational-sensitivity diagnostic and not as a new production estimate of the state distribution. Taken together, the source/spectator controls, B2.10 thermodynamic stress test, and B2.11 matched-MD diagnostic support the same

Table S11: Matched-MD configurational stress test of the interconversion-minus-far p_0 contrast. Confidence intervals use the primary equal-source hierarchical source + 10-ps temporal-block bootstrap.

λ	N_{int}	N_{far}	Δp_0	95% interval	LOO range
0.8	70	119	+0.254033	[+0.109943,+0.400298]	[+0.219169,+0.302737]
1.0	58	119	+0.274435	[+0.166127,+0.368318]	[+0.256013,+0.310639]
1.2	40	119	+0.187969	[+0.031202,+0.351427]	[+0.133940,+0.234985]

limited conclusion: the central mechanistic findings do not depend on a single source segment, on a simple spectator-nitrate selection artifact, or on fine tuning of the transferred Eu–nitrate C_4 coefficients within the tested ±20% range. These analyses do not remove the shared genealogy of the underlying umbrella branches, and they do not convert the conditional ensembles into equilibrium populations.

S7 Complementary multireference electronic-structure results

This section documents the multireference electronic-structure calculations used only as a complementary electronic benchmark for the classical electrostatic analysis. The purpose is to establish the topology and spin–orbit organization of the low-lying Eu^{3+} $4f^6$ manifold and to test the sensitivity of the spin-free energies to quasi-degenerate dynamic correlation. These calculations are not used as an empirical fit to the experimental Eu^{3+} emission spectrum, and the classical odd-rank electrostatic descriptors are not mapped quantitatively onto Judd–Ofelt parameters or oscillator strengths.

S7.1 Benchmark cluster and multireference protocol

All production multireference calculations were performed with OpenMolcas 26.06. The electronic benchmark uses the representative inner-state central configuration (snapshot 103). The quantum region contains Eu^{3+}, the selected nitrate, and eight complete first-shell water molecules, for a total of 29 atoms, formal charge +2, and 172 electrons. The nuclear coordinates were taken directly from the molecular-dynamics configuration and were not geometry optimized. The finite external electrostatic environment is the validated FULL+AUX embedding described in Section S5. ANO-RCC-VDZP basis sets were used for H, N, and O, together with ANO-RCC-VTZP for Eu. Scalar relativistic effects were treated with the Douglas–Kroll–Hess Hamiltonian, two-electron integrals were represented by Cholesky decomposition, and spatial symmetry was disabled (C_1). The multireference active space was CAS(6,7), containing the six Eu $4f$ electrons in seven predominantly Eu-$4f$ orbitals; the remaining 166 electrons occupy 83 inactive orbitals.

Equal-weight state-averaged CASSCF calculations were performed separately for seven septet roots and five quintet roots. These roots span the spin-free parent spaces used to construct the ^7F_J and lowest ^5D_J spin–orbit manifolds. State-specific strongly contracted NEVPT2 (SC-NEVPT2) calculations were then carried out separately for all 12 spin-free roots. A complementary quasi-degenerate SC-NEVPT2 (QD-SC-NEVPT2) calculation was performed independently within each multiplicity manifold. Finally, the seven septet and five quintet spin-free states were coupled by RASSI-SO with AMFI spin–orbit integrals, giving

$$7(2S_{\mathrm{septet}}+1)+5(2S_{\mathrm{quintet}}+1)=7\times 7+5\times 5=74 \tag{S52}$$

spin–orbit states.

To retain differential dynamic correlation in the spin–orbit treatment, the diagonal spin-free energies entering the RASSI Hamiltonian were replaced by the corresponding state-specific SC-NEVPT2 energies. We refer to this construction throughout as *diagonal-energy-corrected SC-NEVPT2/RASSI-SO*. It should not be interpreted as a fully correlated spin–orbit wavefunction optimization.

S7.2 Spin-free CASSCF and state-specific SC-NEVPT2 energies

Table S12 reports the complete set of seven septet and five quintet spin-free roots. Relative energies are referenced to the lowest septet root separately at the CASSCF and SC-NEVPT2 levels, so that the intermanifold separation is visible directly in the same table.

Table S12: Spin-free CASSCF reference and state-specific SC-NEVPT2 energies for the CAS(6,7) benchmark. Relative energies are measured from the lowest septet root at the corresponding electronic-structure level.

Manifold	Root	E_{CASSCF} (E_h)	$\Delta E_{\mathrm{CASSCF}}$ (cm^{-1})	E_{SC} (E_h)	ΔE_{SC} (cm^{-1})
septet	1	-11723.60978477	0.000	-11726.59158592	0.000
septet	2	-11723.60958748	43.301	-11726.59143137	33.919
septet	3	-11723.60933652	98.379	-11726.59105852	115.751
septet	4	-11723.60921332	125.418	-11726.59094733	140.155
septet	5	-11723.60875734	225.494	-11726.59043627	252.320
septet	6	-11723.60828828	328.440	-11726.58981835	387.936
septet	7	-11723.60816170	356.223	-11726.58965018	424.846
quintet	1	-11723.49930464	24247.586	-11726.49025260	22240.094
quintet	2	-11723.49926611	24256.042	-11726.49022426	22246.312
quintet	3	-11723.49920154	24270.212	-11726.49015608	22261.276
quintet	4	-11723.49915137	24281.223	-11726.49010506	22272.474
quintet	5	-11723.49910068	24292.350	-11726.49005877	22282.633

For the lowest roots of the two multiplicities, the spin-free separation is

$$\Delta E^{\mathrm{CASSCF}}_{^5D-^7F}=24247.586\ \mathrm{cm}^{-1}, \tag{S53}$$

$$\Delta E^{\mathrm{SC-NEVPT2}}_{^5D-^7F}=22240.095\ \mathrm{cm}^{-1}=2.757420\ \mathrm{eV}. \tag{S54}$$

Dynamic correlation therefore lowers this spin-free separation by

$$24247.586-22240.095=2007.491\ \mathrm{cm}^{-1}, \tag{S55}$$

which is the origin of the approximately $2.0\times 10^3\ \mathrm{cm}^{-1}$ reduction quoted in the main text. The result is substantial on the scale of the intermanifold separation, whereas the splittings among roots within a given spin multiplicity remain much smaller.

S7.3 State-specific versus quasi-degenerate SC-NEVPT2

The quasi-degenerate calculation was used as a direct sensitivity test of the state-specific SC-NEVPT2 root ordering and intramanifold splittings. For each multiplicity, define

$$\delta_i^{\mathrm{QD-SC}} = \left(E_i^{\mathrm{QD}} - E_1^{\mathrm{QD}}\right) - \left(E_i^{\mathrm{SC}} - E_1^{\mathrm{SC}}\right). \tag{S56}$$

The complete comparison is given in Table S13 and Figure S14.

Table S13: Comparison of state-specific SC-NEVPT2 and QD-SC-NEVPT2 relative spin-free splittings. Within each multiplicity the lowest root is set to zero. The last column is the QD correction defined in Eq. S56.

Manifold	Root	ΔE_{SC} (cm^{-1})	ΔE_{QD} (cm^{-1})	$\delta_i^{\mathrm{QD-SC}}$ (cm^{-1})
septet	1	0.000	0.000	+0.000
septet	2	33.920	45.133	+11.213
septet	3	115.751	122.043	+6.292
septet	4	140.154	148.593	+8.439
septet	5	252.319	260.172	+7.853
septet	6	387.937	395.118	+7.181
septet	7	424.846	431.909	+7.063
quintet	1	0.000	0.000	+0.000
quintet	2	6.218	6.349	+0.132
quintet	3	21.181	21.254	+0.072
quintet	4	32.379	32.612	+0.233
quintet	5	42.539	42.855	+0.316

The QD correction is therefore numerically small compared with both the $\sim 2.22 \times 10^4$ cm^{-1} correlated spin-free intermanifold gap and the approximately 2.01×10^3 cm^{-1} change introduced by dynamic correlation itself. The state-specific SC-NEVPT2 description used to construct the spin–orbit Hamiltonian is consequently not contingent on a large quasi-degenerate reordering of the selected roots.

S7.4 Spin–orbit manifold and character of the $^5D_0$-dominated state

The 74 diagonal-energy-corrected SC-NEVPT2/RASSI-SO eigenstates organize into the expected Eu^{3+} $4f^6$ term manifolds. The first 49 states form groups with multiplicities $1, 3, 5, 7, 9, 11, 13$, exactly matching $2J + 1$ for ^7F_J with $J = 0, \ldots, 6$. The remaining 25 states form groups with multiplicities $1, 3, 5, 7, 9$, matching ^5D_J with $J = 0, \ldots, 4$. Table S14 summarizes the group positions and crystal-field spreads, while Table S15 gives all 74 individual spin–orbit energies.

The lowest member of the ^5D block is SO state 50 at

$$E_{^5D_0\mathrm{-dominated}} = 20906.338\ \mathrm{cm}^{-1} = 2.592056\ \mathrm{eV}. \tag{S57}$$

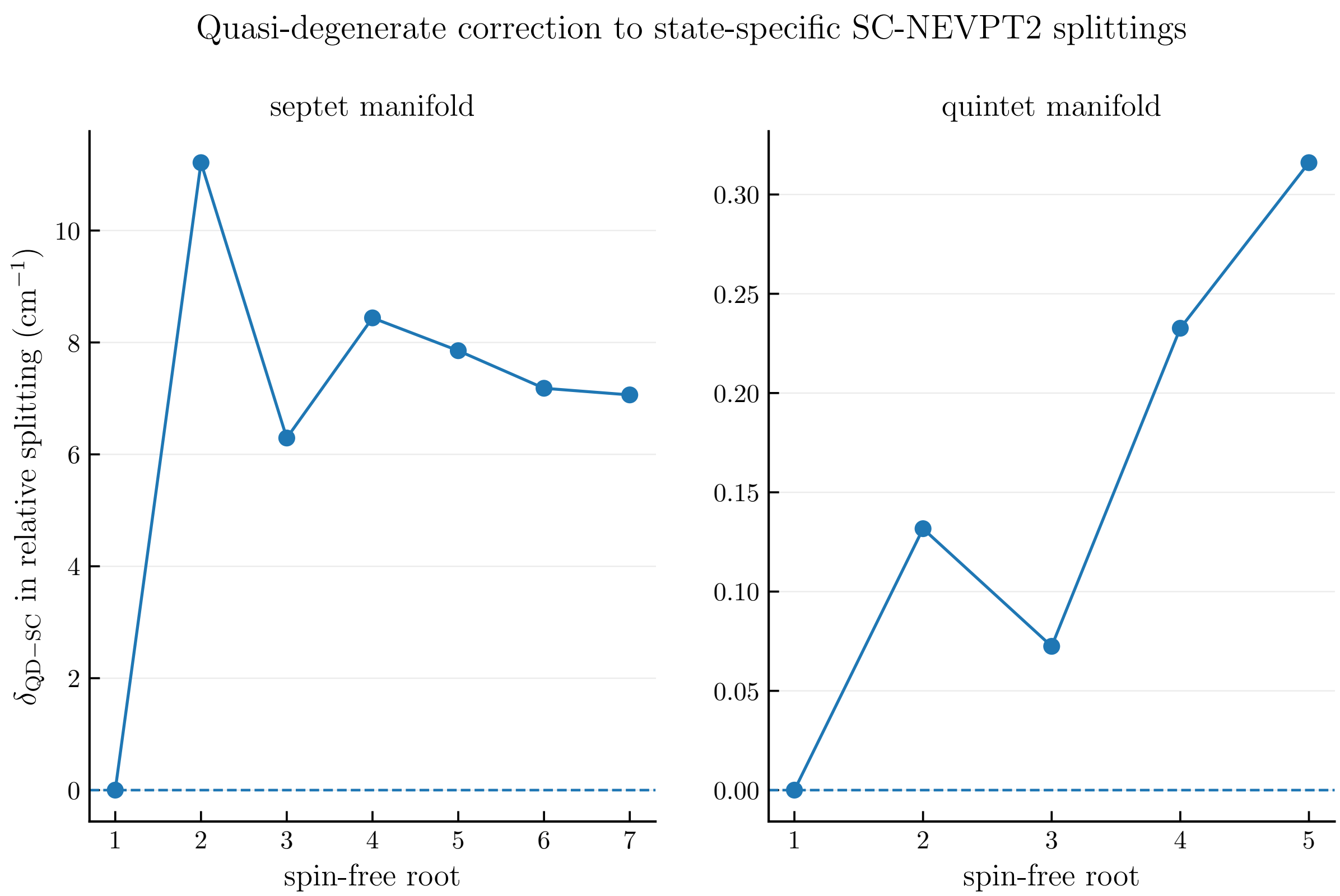

Figure S14: Quasi-degenerate correction to the relative spin-free splittings obtained from state-specific SC-NEVPT2. The ordinate is δ_i^{QD-SC} from Eq. S56. The largest absolute rearrangement is 11.213 cm^{-1} in the septet manifold and only 0.316 cm^{-1} in the quintet manifold. Thus the quasi-degenerate treatment produces only minor intramanifold changes on the energy scales relevant to the ^7F–^5D separation.

Table S14: Organization of the 74 diagonal-energy-corrected SC-NEVPT2/RASSI-SO states. The group center is the arithmetic mean of the individual SO energies within the indicated J manifold; "min" and "max" give its resolved crystal-field range.

Assignment	SO states	n	Min. (cm^{-1})	Center (cm^{-1})	Max. (cm^{-1})
$^7F_0$	1–1	1	0.000	0.000	0.000
$^7F_1$	2–4	3	339.864	393.933	447.979
$^7F_2$	5–9	5	1086.198	1133.384	1181.883
$^7F_3$	10–16	7	2104.247	2141.795	2178.623
$^7F_4$	17–25	9	3228.279	3346.298	3494.629
$^7F_5$	26–36	11	4589.528	4685.208	4803.463
$^7F_6$	37–49	13	5991.260	6110.303	6192.592
$^5D_0$	50–50	1	20906.338	20906.338	20906.338
$^5D_1$	51–53	3	21784.211	21791.495	21799.277
$^5D_2$	54–58	5	23594.852	23607.398	23618.257
$^5D_3$	59–65	7	26413.564	26429.599	26441.618
$^5D_4$	66–74	9	30324.335	30338.126	30354.579

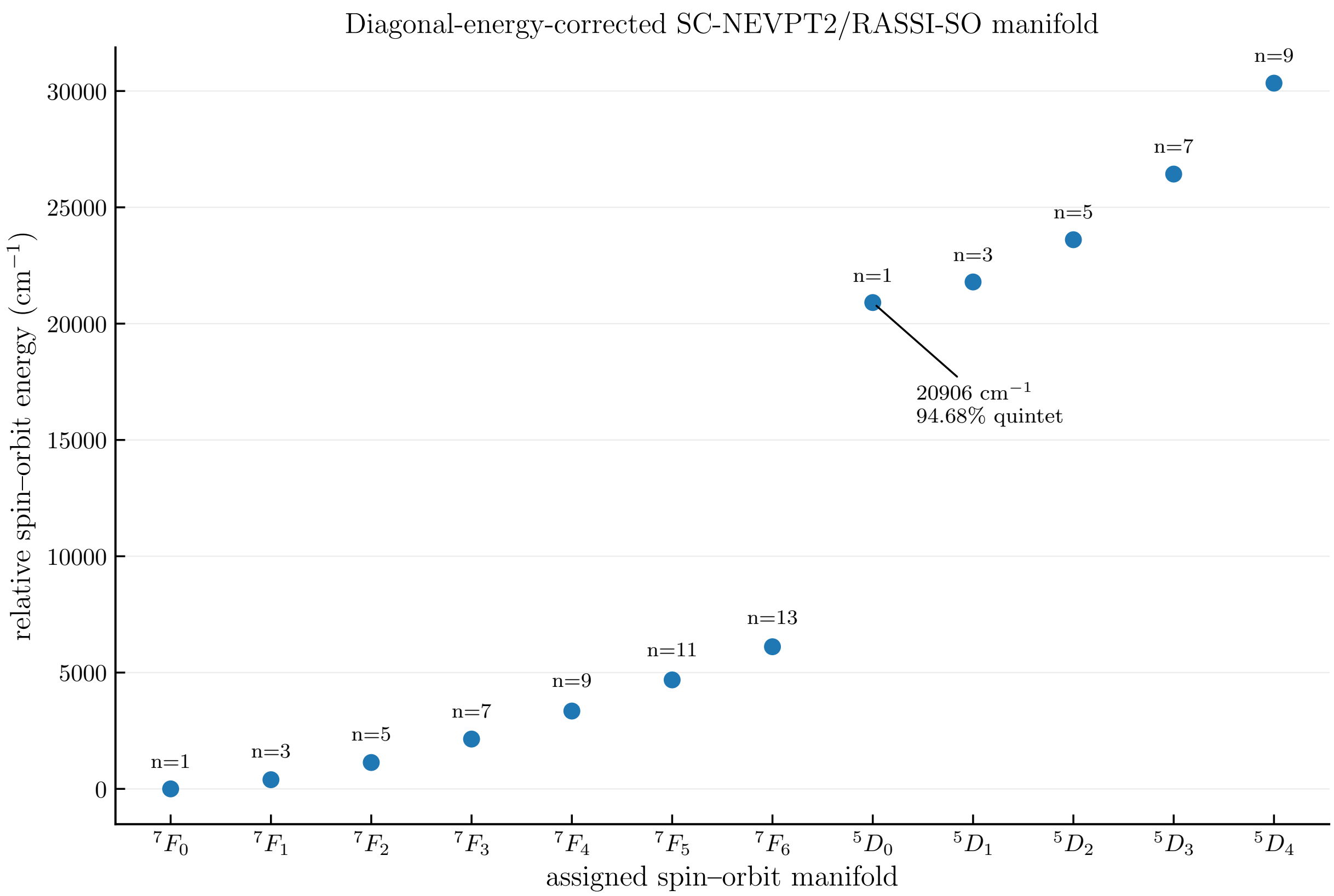

Figure S15: Organization of the 74 diagonal-energy-corrected SC-NEVPT2/RASSI-SO states into the ^7F_J and ^5D_J manifolds. Symbols mark the group centers and vertical bars span the minimum-to-maximum energy within each J group; labels give the corresponding multiplicity $n = 2J + 1$. The $^5D_0$-dominated SO state occurs at 20906.338 cm^{-1} (2.592056 eV) relative to the lowest SO state and is 94.68% quintet by summed spin-free-state weight.

The five quintet spin-free parents included in the RASSI basis carry weights 0.1911, 0.1906, 0.1902, 0.1879, and 0.1870 in this SO eigenstate. Their sum is

$$w_{\text{quintet}} = 0.9468, \qquad w_{\text{septet}} = 1 - w_{\text{quintet}} = 0.0532, \tag{S58}$$

or 94.68% quintet and 5.32% septet. The computed $^5D_0$-dominated energy remains higher than required for a quantitatively accurate reproduction of the experimental Eu^{3+} $^5D_0$ emission region. Accordingly, the present calculation is used to establish electronic-state topology, multiplicities, and mixing rather than as an empirical spectral fit.

Table S15: Complete diagonal-energy-corrected SC-NEVPT2/RASSI-SO state list. Energies are relative to SO state 1. Assignments denote the J group identified from the contiguous $2J + 1$ multiplicity structure.

SO state	Assignment	Relative energy (cm^{-1})
1	$^7F_0$	0.000
2	$^7F_1$	339.864
3	$^7F_1$	393.954
4	$^7F_1$	447.979
5	$^7F_2$	1086.198
6	$^7F_2$	1104.062
7	$^7F_2$	1143.303
8	$^7F_2$	1151.474
9	$^7F_2$	1181.883
10	$^7F_3$	2104.247
11	$^7F_3$	2107.000
12	$^7F_3$	2125.553
13	$^7F_3$	2151.981
14	$^7F_3$	2154.843
15	$^7F_3$	2170.317
16	$^7F_3$	2178.623
17	$^7F_4$	3228.279
18	$^7F_4$	3275.099
19	$^7F_4$	3286.912
20	$^7F_4$	3318.727
21	$^7F_4$	3333.988
22	$^7F_4$	3371.236
23	$^7F_4$	3397.485
24	$^7F_4$	3410.329
25	$^7F_4$	3494.629
26	$^7F_5$	4589.528
27	$^7F_5$	4596.467
28	$^7F_5$	4623.010

Continued on next page

Table S15 continued

SO state	Assignment	Relative energy (cm^{-1})
29	$^7F_5$	4626.542
30	$^7F_5$	4651.944
31	$^7F_5$	4684.740
32	$^7F_5$	4703.833
33	$^7F_5$	4731.161
34	$^7F_5$	4756.724
35	$^7F_5$	4769.874
36	$^7F_5$	4803.463
37	$^7F_6$	5991.260
38	$^7F_6$	5991.607
39	$^7F_6$	6059.964
40	$^7F_6$	6064.447
41	$^7F_6$	6091.241
42	$^7F_6$	6108.088
43	$^7F_6$	6117.404
44	$^7F_6$	6144.098
45	$^7F_6$	6152.503
46	$^7F_6$	6162.827
47	$^7F_6$	6165.847
48	$^7F_6$	6192.064
49	$^7F_6$	6192.592
50	$^5D_0$	20906.338
51	$^5D_1$	21784.211
52	$^5D_1$	21790.997
53	$^5D_1$	21799.277
54	$^5D_2$	23594.852
55	$^5D_2$	23602.702
56	$^5D_2$	23609.407
57	$^5D_2$	23611.770
58	$^5D_2$	23618.257
59	$^5D_3$	26413.564
60	$^5D_3$	26419.843
61	$^5D_3$	26425.641
62	$^5D_3$	26433.400
63	$^5D_3$	26434.517
64	$^5D_3$	26438.611
65	$^5D_3$	26441.618
66	$^5D_4$	30324.335

Continued on next page

Table S15 continued

SO state	Assignment	Relative energy (cm^{-1})
67	$^5D_4$	30325.018
68	$^5D_4$	30329.115
69	$^5D_4$	30332.171
70	$^5D_4$	30335.441
71	$^5D_4$	30344.204
72	$^5D_4$	30345.709
73	$^5D_4$	30352.558
74	$^5D_4$	30354.579

S7.5 Exploratory opposite-parity active space and scope limitation

CAS(6,7) intentionally describes the Eu $4f^6$ manifold and does not contain explicit $4f^6 \leftrightarrow 4f^5 5d^1$ opposite-parity configurations. An exploratory generalized-active-space calculation was therefore carried out using the same benchmark geometry with seven predominantly Eu-$4f$ orbitals and five Eu-d-like orbitals identified from improved virtual orbitals. A constrained GAS occupation allowing the $4f^5 5d^1$ sector confirmed that this configuration space is numerically accessible. The completed exploratory calculation retained the expected 172-electron, +2-charge system and generated natural-orbital output for 20 roots. The extended active space did not, however, provide a balanced five-orbital Eu-$5d$ shell under the required low-state averaging. Orbital inspection showed that the d-electron density concentrated predominantly in only two crystal-field components, whereas the remaining nominal $5d$ orbitals became nearly unoccupied and rotated strongly toward secondary ligand space. This instability prevents the resulting roots from being used as a quantitatively controlled opposite-parity manifold.

For clarity, Mulliken $4f/5d$ *spin populations* printed in the exploratory GASSCF output are not interpreted as natural-orbital occupations. Likewise, velocity-form transition strengths printed automatically by RASSI-SO for the CAS(6,7) calculation are deliberately excluded from the quantitative analysis. Without a balanced explicit opposite-parity active space, those values do not constitute a controlled calculation of forced-electric-dipole intensity borrowing. Consequently, the exploratory $4f^5 5d^1$ calculation is not used to report transition energies, oscillator strengths, Judd–Ofelt Ω_2 parameters, or any other quantitative intensity observable. The connection between odd-rank classical electrostatic descriptors and opposite-parity intensity borrowing is therefore retained only as a symmetry-constrained mechanistic hypothesis. Odd-parity environmental components are symmetry-relevant for such mixing, but their presence alone is not sufficient to determine a luminescence intensity.

The machine-readable files generated by the final post-processing audit include the full spin-free SC-NEVPT2 table, the complete state-specific versus QD-SC-NEVPT2 comparison, the grouped and state-resolved RASSI-SO tables, and the plotting data used for Figures S14 and S15. No new electronic-structure calculation was performed in producing these SI summaries.
